# NERNST: a genetically-encoded ratiometric non-destructive sensing tool to estimate NADP(H) redox status in bacterial, plant and animal systems

**DOI:** 10.1038/s41467-023-38739-4

**Published:** 2023-06-06

**Authors:** Pamela E. Molinari, Adriana R. Krapp, Andrea Weiner, Hannes M. Beyer, Arun Kumar Kondadi, Tim Blomeier, Melina López, Pilar Bustos-Sanmamed, Evelyn Tevere, Wilfried Weber, Andreas S. Reichert, Nora B. Calcaterra, Mathias Beller, Nestor Carrillo, Matias D. Zurbriggen

**Affiliations:** 1 Instituto de Biología Molecular y Celular de Rosario (IBR-UNR/CONICET), Facultad de Ciencias Bioquímicas y Farmacéuticas, Universidad Nacional de Rosario (UNR), 2000 Rosario, Argentina; 2 Institute of Synthetic Biology, University of Düsseldorf, Düsseldorf, Germany; 3 Institute of Biochemistry and Molecular Biology I, Medical Faculty and University Hospital Düsseldorf, Heinrich-Heine-University Düsseldorf, Düsseldorf, Germany; 4 Faculty of Biology and Signalling Research Centres BIOSS and CIBSS, University of Freiburg, Freiburg, Germany; 5 Institute of Mathematical Modeling of Biological Systems, University of Düsseldorf, Düsseldorf, Germany; 6 CEPLAS – Cluster of Excellence on Plant Sciences, Düsseldorf, Germany; 7 Present Address: INM - Leibniz Institute for New Materials and Department of Materials Sciences and Engineering, Saarland University, Saarbrücken, Germany

**Keywords:** Fluorescent proteins, Enzymes, Synthetic biology, Small molecules

## Abstract

NADP(H) is a central metabolic hub providing reducing equivalents to multiple biosynthetic, regulatory and antioxidative pathways in all living organisms. While biosensors are available to determine NADP^+^ or NADPH levels in vivo, no probe exists to estimate the NADP(H) redox status, a determinant of the cell energy availability. We describe herein the design and characterization of a genetically-encoded ratiometric biosensor, termed NERNST, able to interact with NADP(H) and estimate *E*_NADP(H)_. NERNST consists of a redox-sensitive green fluorescent protein (roGFP2) fused to an NADPH-thioredoxin reductase C module which selectively monitors NADP(H) redox states via oxido-reduction of the roGFP2 moiety. NERNST is functional in bacterial, plant and animal cells, and organelles such as chloroplasts and mitochondria. Using NERNST, we monitor NADP(H) dynamics during bacterial growth, environmental stresses in plants, metabolic challenges to mammalian cells, and wounding in zebrafish. NERNST estimates the NADP(H) redox poise in living organisms, with various potential applications in biochemical, biotechnological and biomedical research.

## Abstract (web summary)

NADP(H) is a crucial cofactor, acting as a reducing agent in numerous pathways in living organisms. Here the authors report a ratiometric biosensor named NERNST, which can be used to estimate the NADP(H) redox status in bacterial, plant and animal cells and organelles.

## Introduction

NADPH provides the reducing power for most cellular biosynthetic reactions, contributes to antioxidative defense mechanisms and plays key roles in signaling pathways, integrating developmental and environmental stimuli in eukaryotes, bacteria and archaea^1–6^. Enzymes catalyzing NADP(H)-associated reactions are distributed in different cellular compartments. Therefore, spatiotemporally-resolved detection of NADP(H) levels and redox states in living cells and subcellular compartments (as defined by the NADPH/NADP^+^ ratio or the redox potential *E*_NADP(H)_) is crucial for understanding the role of this cofactor in central and secondary metabolism, regulation and defense, and to monitor the energy status of the cell. Conventional methods for NADP(H) determination usually involve tissue destruction, are prone to sample oxidation during processing, display limited specificity and do not discriminate between cellular compartments^7–10^. Thus, non-invasive quantitative molecular tools are much needed.

Genetically-encoded fluorescent sensors could overcome these limitations by detecting defined targets in specific tissues, cells and subcellular compartments. SoNar biosensors have been designed for both NADH^11,12^ and NAD^+^ (ref. ^13^), and can in combination estimate NADH/NAD^+^ ratios in vivo^14,15^. They are based on binding of the dinucleotides to the redox-sensing repressor Rex, which regulates transcription of respiratory genes in bacteria in response to the intracellular NADH/NAD^+^ redox poise. This ligand-binding domain was fused to two circularly-permuted yellow fluorescent proteins (cpYFP), thereby coupling the conformational changes caused by NAD(H) docking to changes in fluorescence^11–15^. SoNar has been further engineered to switch its nucleotide specificity, generating the iNap series of NADPH probes^16^. NADP^+^ levels, in turn, could be determined by fluorescence resonance energy transfer (FRET) using Apollo-NADP^+^, a biosensor based on NADP^+^-dependent homodimerization of glucose-6-phosphate dehydrogenase (G6PDH) fused to different fluorescent proteins^17^. However, no sensor is currently available to directly report on cellular NADP(H) redox status. Smith et al.^18^ have recently identified this limitation as a major pending issue, and indicated that sensors able to determine NADPH:NADP^+^ ratios and NADP(H) redox potentials (*E*_NADP(H)_) are badly needed to properly estimate energy availability in living organisms.

Ratiometric probes are particularly well suited for in vivo imaging. By using two excitation wavelengths they generate an internally normalized signal that is independent of biosensor expression levels^18,19^. Redox-sensitive ratiometric sensors based on green/red/yellow fluorescent proteins (for instance, roGFP2 and rxRFP1) have been prepared by introducing vicinal cysteines in GFP/cpRFP that can equilibrate with cellular thiols, most conspicuously glutathione (GSH)^20–23^, but also thioredoxin (Trx)^24^. Fusion with various redox partners allowed changes in specificity and fast equilibration rates^25,26^.

We target here the development of a biosensor suitable to determine the cellular NADP(H) redox status. For this, we combine two molecular modules: a rice NADPH-dependent thioredoxin reductase C (NTRC) and roGFP2 as NADPH sensing and output components, respectively. We thus exploit the ability of NTRC to mediate disulfide reduction specifically by NADPH^27^ to generate a probe that can monitor the NADP(H) redox status through oxido-reduction of vicinal dithiols/disulfides in roGFP2, which can be determined by live imaging or fluorescence spectroscopy. NTRC is fused in-frame to the N-terminal end of roGFP2 to yield NERNST (**N**ADP(H)-**e**stimating **r**atiometric **n**on-destructive **s**ensing **t**ool). We show that NERNST maintains the ratiometric properties of roGFP2, and productively interacts with NADP(H) in vitro and in vivo. The fluorescent biosensor can be easily customized for use in mid-throughput experimental set-ups for pharmacological and genetic studies and in combination with advanced confocal microscopy approaches. We illustrate the potential applicability of NERNST in fundamental research and biotechnological practice through various proof-of-concept experiments in bacterial, plant and animal cells, in subcellular compartments including chloroplasts and mitochondria, and in whole plants and zebrafish.

## Results

### Design and in vitro characterization of NERNST

**Fig. 1:**
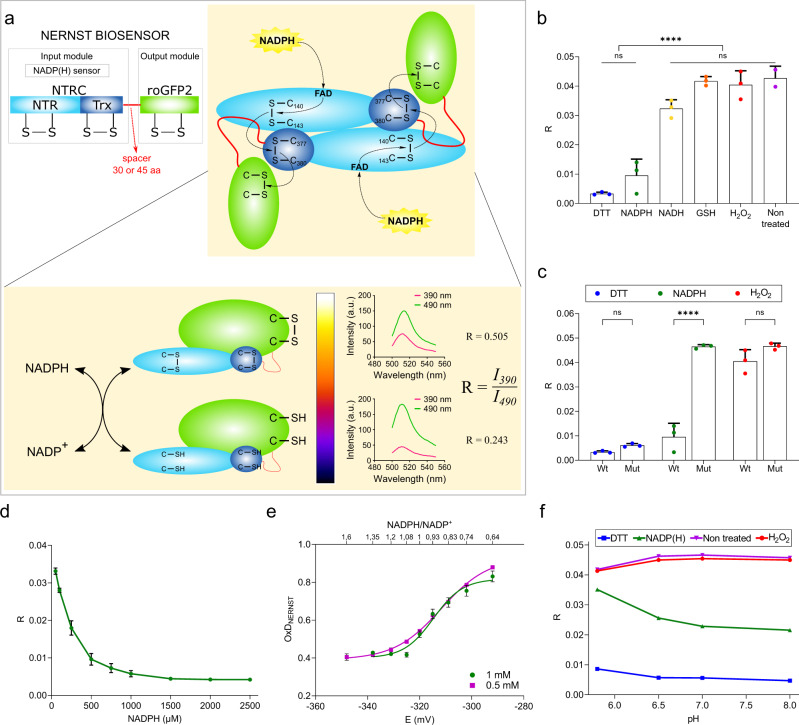
Design, construction and in vitro characterization of NERNST. **a** Proposed mechanism of NERNST interaction and electron exchange with NADP(H). Molecular organization of the input (NTRC with NTR and Trx domains) and output (roGFP2) modules of the biosensor, separated by the spacer arm (top, left), and the intra-complex flow of electrons from NADPH to the vicinal dithiols of a second NERNST subunit, as proposed in ref. ^27^ (top, right). Numbering of active site cysteines corresponds to that of NTRC^27^. Changes resulting from the NADPH-dependent turnover of NERNST and emission spectra of the fully oxidized (above) and reduced (below) biosensor are shown for comparison (bottom). The color bar reflects the oxidation state (R values) in pseudocolors. This applies to all figures. **b** Redox state, as estimated by the R values, of NERNST equilibrated with various oxidants and reductants in 100 mM K_3_PO_4_ pH 7, 150 mM NaCl. DTT and H_2_O_2_ were used at 10 mM; NADPH, NADH and GSH at 0.5 mM. **c** The Cys-to-Ser mutant of NERNST (Mut) is not reduced by NADPH. Conditions were those of panel (**b**). Wt, wild-type biosensor. **d** Reduction of NERNST by different concentrations of NADPH. **e** Plot of *OxD*_NERNST_ (as determined from the *R* values) against the redox potential *E*_NADP(H)_ (**E**). The biosensor was equilibrated with different NADPH/NADP^+^ ratios at 0.5 mM or 1 mM total NADP(H) concentration. The upper abscissa represents the ratio of NADPH/NADP^+^. **f** Effect of pH on the ratiometric behavior of purified NERNST. The biosensor was incubated with 10 mM DTT, 10 mM H_2_O_2_ or an equimolar NADPH/NADP^+^ mixture of 1 mM NADP(H) in a buffer containing 0.2 M NaH_2_PO_4_ and 0.2 M Na_2_HPO_4_ adjusted to the indicated pH. SD values were within the size of the data points. Data shown in (**b**–**f**) are means ± SD of 3 independent determinations. ****P ≤ 0.0001; ns, non-significant. **b** One-way ANOVA followed by Tukey’s multiple comparisons test, **c** Two-way ANOVA followed by Bonferroni’s multiple comparisons test. Source data, including exact *P* values, are provided as a Source Data file.

To generate a genetically-encoded molecular probe capable of transducing reducing equivalents between the universe of pyridine nucleotides to that of dithiols/disulfides, we fused rice NTRC, the NADP(H) sensor, to the N-terminus of roGFP2, the output module (Fig. 1a; Supplementary Tables 1-3). The two components were connected by spacers of 30 or 45 amino acids (aa) to probe different geometries through a flexible interaction between the linked modules (Fig. 1a; Supplementary Table 2; see Methods). NTRC has a built-in Trx domain at the C-terminal end of a canonical NADPH-Trx reductase (NTR) domain^28^. Inactive NERNST versions were generated for control purposes by replacing active-site cysteines by serines in both the NTR and Trx domains using site-directed mutagenesis (Supplementary Fig. 1a).

NTRC has been proposed to act as a homodimer with head-to-tail orientation, so that reducing equivalents are conveyed from the NTR domain of one subunit to the Trx domain of the other^27^. Accordingly, we propose the reaction mechanism depicted in Fig. 1a for NERNST, and predict that electron exchange between the sensor and the NADP(H) pool should lead to changes in the fluorescence of the roGFP2 moiety (Fig. 1a, see below). To characterize the optical properties and redox performance of NERNST, wild-type (Wt) and mutant versions of the biosensor were cloned in the plasmid pET-TEV under control of the T7 promoter (Supplementary Fig. 1a) and expressed in chaperone-rich *Escherichia coli* strains (Supplementary Fig. 1b). Histidine-tagged recombinant products were purified by affinity chromatography (Supplementary Fig. 1c–e). Dimers were the most abundant form, although variable amounts of monomer were also recovered from Ni-resins as revealed by native PAGE (Supplementary Fig. 1f). Dimeric forms were separated from the monomers by size-exclusion chromatography (Supplementary Fig. 1d, f), and used as purified protein samples for in vitro characterization.

Absorption spectra of purified NERNST displayed maxima at 387 nm and 488 nm (Supplementary Fig. 2). The overall shape of the spectrum suggests that the probe was largely oxidized during purification by reaction with oxygen and indeed, little or no spectral changes were observed upon addition of 10 mM H_2_O_2_ (Fig. 1b; Supplementary Fig. 2). Chemical reduction with excess dithiothreitol (DTT), instead, led to virtual disappearance of the 387-nm peak and a moderate increase at the 488-nm region (Supplementary Fig. 2). Extinction coefficients (ε) of 13.0 mM^−1^ cm^−1^ and 21.8 mM^−1^ cm^−1^ at 488 nm were determined for the oxidized and reduced forms, respectively, whereas the 387-nm peak of oxidized NERNST had ε_387_ = 17.7 mM^−1^ cm^−1^ (Supplementary Table 4).

The fluorescence excitation spectrum of purified NERNST displayed features similar to the absorption spectrum, with excitation maxima at 390 nm and 490 nm (Supplementary Fig. 3a), and a single emission peak around 510 nm (Supplementary Fig. 3b). As expected, DTT reduction obliterated most of the 390-nm excitation peak, accompanied by a fluorescence increase at the 490-nm region, with the anticipated impact on the emission spectra (Supplementary Fig. 3c, d; Fig. 1b). Quantum yields increased from 0.66 to 0.91 upon reduction, with a concomitant enhancement in NERNST brightness (Supplementary Table 4). Spectral characteristics were thus similar to those displayed by other roGFP-based biosensors^21^, indicating that the optical properties of the fluorescent protein were not significantly affected by fusion with NTRC.

The ratio R of the fluorescence emission intensities after excitation at the two maxima (390 nm and 490 nm; *R* = *I*_390_/*I*_490_) reflects the oxidation state of the probe (*OxD*_NERNST_; see Methods), with *R* values increasing with *OxD*_NERNST_ as exemplified in Fig. 1a. The actual excitation wavelengths used in the calculations will depend on the equipment configuration (see below). On the other hand, the difference in the *R* values of the fully oxidized and reduced forms of the probe (*R*_ox_–*R*_red_), obtained by chemical reaction with strong oxidants and reductants, defines the dynamic range (DR) of measurements for each system^25,26^.

Incubation of NERNST dimers with 500 μM NADPH led to rapid reduction of the probe, as shown by the decline in the *R* value (Fig. 1b). Dimer variants with flexible arms of 30 or 45 aa yielded essentially the same results (Supplementary Fig. 4). NERNST reduction was complete in about 3 min (Supplementary Fig. 5a). Under the same conditions, the biosensor was not significantly reduced by NADH or GSH (Fig. 1b). Cys-to-Ser mutations in NTRC completely abolished the NADPH effect, indicating that the NTRC moiety mediates roGFP2 reduction (Fig. 1c). As with other roGFP2-based bioprobes^19–21,24,25^, the mutant NERNST versions can still react chemically with DTT or H_2_O_2_, rendering the totally reduced and oxidized forms of the roGFP2 moiety, respectively (Fig. 1c). Reduction by NADPH was concentration-dependent, and virtually complete at 1 mM under the conditions employed (Fig. 1d). Unlike the dimeric forms, monomeric NERNST did not react with NADPH (Supplementary Fig. 5b, c). This observation agrees with the catalytic mechanism proposed for NTRC^27^, and lends support to the model of Fig. 1a.

As shown in Supplementary Fig. 5, NERNST could be fully reduced by a brief incubation with DTT. After DTT removal by gel filtration, the reduced biosensor was slowly oxidized by dissolved oxygen, but reacted several-fold faster with NADP^+^ (Supplementary Fig. 6a, b), even though interaction with NADP(H) would favor reduction, assuming a standard redox potential (${{E}^{0{\prime} }}_{{{{{{{\rm{NERNST}}}}}}}}$) of about −280 mV for this roGFP2-derived probe^25,26,29,30^. Successive additions of NADPH and NADP^+^ led to the expected changes in NERNST redox status (Supplementary Fig. 6c), confirming that the reaction was fully reversible. When oxidized NERNST was incubated with different NADPH/NADP^+^ mixtures at two NADP(H) concentrations, the degree of sensor oxidation (*OxD*_NERNST_) increased with the fraction of NADP^+^ in the mix, and a plot of *OxD*_NERNST_ versus *E*_NADP(H)_, calculated from the corresponding NADPH/NADP^+^ ratios using the Nernst equation (see Methods), resulted in a typical titration curve for both 0.5 mM and 1 mM NADP(H) (Fig. 1e). The *R* values obtained at a 1:1 NADPH/NADP^+^ ratio did not vary significantly between pH 6.5 and 8.0, but showed a moderate increase below pH 6.5 (Fig. 1f). Fluorescence emissions of the purified, fully oxidized and fully reduced sensors were largely insensitive to pH changes in the range assayed (Fig. 1f).

Rates of NERNST oxidation increased with NADP^+^ concentration (Supplementary Fig. 6b–d). Upon completion of the reaction, R values were similar to those of oxidized NERNST, although the initial R values were higher than the ones measured for the fully reduced biosensor (Supplementary Fig. 6d). Most likely, these differences resulted from slow oxidation of the probe by dissolved O_2_ during the manipulations that preceded fluorescent measurements. Unlike NADP^+^, reaction of NERNST with plant or bacterial thioredoxins was very slow, not appreciably different from spontaneous oxidation (Supplementary Fig. 7), suggesting that thioredoxins are not significant redox partners for the biosensor.

The kinetics of NERNST reduction by NADPH was largely unaffected by the presence of other redox-active metabolites such as GSH, GSSG, NADH, NAD^+^ or cysteine (Supplementary Fig. 8). Many developmental and environmental stimuli trigger cellular responses involving changes in H_2_O_2_ concentrations, as a key signaling molecule in multiple physiological processes^31–33^. It was therefore interesting to determine if NERNST output could be affected by H_2_O_2_. While the reduced biosensor did react with H_2_O_2_ in the presence of NADPH, oxidation only became apparent at 6 mM H_2_O_2_ (Supplementary Fig. 9). Cellular levels of this oxidant have been reported at the low nM range in bacteria^34^, plants^35^ and mammals^36,37^, suggesting that this interference should be negligible in vivo.

If the fluorescence intensities of the fully reduced and oxidized forms of the biosensor can be determined experimentally, the resulting data can be employed to calculate *OxD*_NERNST_^25,26^ (see Eq. (2) in Methods), and the NADP(H) redox potential *E*_NADP(H)_ estimated from *OxD*_NERNST_ using the Nernst equation:

$${E}_{{{{{{{\rm{NADP}}}}}}}\left(H\right)}={{E}^{0{\prime} }}_{{{{{{{\rm{NADP}}}}}}}\left(H\right)}-\frac{{RT}}{{nF}}{{{{\mathrm{ln}}}}}\frac{\left[{{{{{{\rm{NADPH}}}}}}}\right]}{\left[{{{{{{{\rm{NADP}}}}}}}}^{+}\right]}={{E}^{0{\prime} }}_{{{{{{{\rm{NERNST}}}}}}}}-\frac{{RT}}{{nF}}{{{{\mathrm{ln}}}}}\frac{\left({1-{OxD}}_{{{{{{{\rm{NERNST}}}}}}}}\right)}{\left({{OxD}}_{{{{{{{\rm{NERNST}}}}}}}}\right)}={E}_{{{{{{{\rm{NERNST}}}}}}}}$$

Living organisms are open systems, and cellular redox pools are normally not in equilibrium with each other^38^, although redox pairs might remain in steady-state if the overall physiological conditions are stable (Supplementary note). This does not preclude the use of redox-responsive probes to estimate redox potentials, which can provide a measure of the oxido-reduction status at a defined condition, as it is extensively documented and discussed in the literature^25,29,30,39–46^ (see also the Supplementary note). Chloroplast *E*_GSH_, for instance, could be reported by roGFP2 even by whole-plant imaging^45^. We favor the use of *E*_NADP(H)_ over *R* values whenever possible, because redox potentials capture important features of redox systems that cannot be accounted for by *R* values, most remarkably, the key effect of the pH on the redox drive, which becomes particularly relevant when comparing compartments with pH differences, or biological processes involving pH shifts.

The results thus indicate that NERNST can provide estimations of the redox state of the NADP(H) pool and be used, in principle, to determine *E*_NADP(H)_ in living organisms. In the following, we illustrate the application of this probe to various biological systems. Both the 30-aa and 45-aa NERNST variants could be expressed in the cytosol of bacterial, plant and animal cells, but only the NERNST version with the shorter linker accumulated in chloroplasts and mitochondria (see below). Supplementary Table 2 shows the specific variant(s) used and reported in each system.

### Estimation of NADP(H) redox changes in bacterial cells

**Fig. 2:**
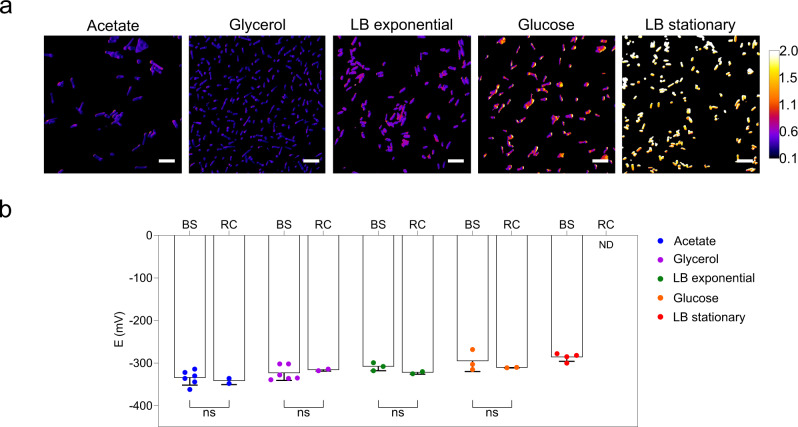
NADP(H) redox state in *E. coli* cells. **a** Bacteria expressing NERNST were grown in Luria-Bertani (LB) broth or fed with acetate, glycerol or glucose in M9 minimal medium. Cells were collected at mid-exponential phase, except for LB stationary. Representative images show the ratios R of the intensities of fluorescence emission after excitation of the cell suspensions at 405 nm and 488 nm in pseudocolors. Scale bar = 5 μm. Pseudocolor scale = R values. Confocal images are ordered from the lower to the higher redox potentials. **b** Estimation of *E*_NADP(H)_ (E) in the *E. coli* cytosol as determined by NERNST biosensor imaging (BS) and redox cycling (RC). Data shown are means ± SD of 2–6 independent determinations. ns, non-significant; two-way ANOVA followed by Bonferroni’s multiple comparisons test. ND, not determined. Source data, including all precise *n* values, are provided as a Source Data file.

The redox-sensing properties of NERNST were first investigated in *E. coli* cells expressing the biosensor under control of the T7 promoter (Supplementary Fig. 1a) by monitoring fluorescence changes through laser-scanning confocal microscopy. We evaluated the capability of the sensor to estimate the NADP(H) redox poise in cells growing on different metabolic regimes. Typical results are shown in Fig. 2a and Supplementary Fig. 10a. NERNST showed significant fluorescence in all conditions, suggesting that assembly of the fused protein was not impaired by nutrient availability. Incubation of the cells with DTT or H_2_O_2_ led to the expected fluorescence changes (Supplementary Fig. 10a,b), allowing determination of *OxD*_NERNST_ and eventually *E*_NADP(H)_ (Fig. 2b). Within this context, changes in biosensor fluorescence are considered to provide reliable calculations of redox potential between 5 and 95% probe oxidation^30^, which would correspond to a window of 80 mV around an assumed ${{E}^{0{\prime} }}_{{{{{{{\rm{NERNST}}}}}}}}$ of −280 mV at pH 7. A pH correction needs to be applied to *OxD*_NERNST_ values, corresponding to −6 mV for each 0.1 unit above pH 7^30^. This would represent a useful range between −270 mV and -350 mV for *E. coli*, assuming a cytosolic pH of 7.5^47^.

When *E. coli* cells are cultured aerobically in rich media such as the Luria-Bertani (LB) broth, the availability of nutrients supports rapid growth via glycolysis, the pentose phosphate pathway (PPP) and the tricarboxylic acid (TCA) cycle^48^. These pathways provide the bulk of NADPH (Supplementary Fig. 11), whose levels are reported to increase during the first hours of growth to progressively decline as cells enter the stationary phase^48^. Accordingly, a redox potential *E*_NADP(H)_ of -308 mV was estimated with NERNST at the exponential phase (Fig. 2a, b). Oxidation of the NADP(H) pool became evident as bacteria entered the stationary phase (Fig. 2a), with *E*_NADP(H)_ increasing to −286 mV (Fig. 2b).

Growth of *E. coli* in minimal media allows studies on the metabolic effects of different carbon sources. Cultures in acetate display a number of characteristic features, such as the induction of the glyoxylate pathway and of the NADP^+^-dependent malic enzyme encoded by the *maeB* gene, which accounts for most NADPH production^49^ (Supplementary Fig. 12). Fluorescence measurements of cells expressing NERNST yielded an *E*_NADP(H)_ of -335 mV at the exponential phase of growth (Fig. 2a, b), indicating that the NADP(H) pool was indeed more reduced under these culture conditions than in LB medium.

Glycerol enters central metabolism as glyceraldehyde-3-phosphate^50^, delivering carbon backbones to the PPP, MaeB and the TCA cycle, which is the major NADPH source under these growth conditions (Supplementary Fig. 13). When bacteria were grown in glycerol as the sole carbon source, the redox potential calculated using NERNST was slightly less negative (*E*_NADP(H)_ = −324 mV) than that obtained in acetate (Fig. 2a, b).

During aerobic growth with glucose, most NADPH is generated through the PPP and the TCA cycle (Supplementary Fig. 14), although both pathways proceed at lower rates compared to those expected during growth from other carbon sources^48^. Accordingly, the NADP(H) redox potential of glucose-fed cells estimated using NERNST was *E*_NADP(H)_ = −295 mV (Fig. 2a, b). DR values between 0.9 and 1.4 were obtained for all conditions except for cells grown in glucose, which showed a rather small dynamic range (Supplementary Fig. 10b). Aerobic culture in glucose minimal medium led to lower NERNST expression levels compared to other growth media, presumably reflecting the extensively documented impairment of *lacZ* induction by glucose (reviewed by Studier^51^). As a general feature, dynamic ranges varied widely in different biological systems (see below). The mechanistic origin of these variations is unknown, but is a common feature of roGFP2-based biosensors^22,29,52,53^. Variable autofluorescence (especially at the lower-wavelength excitation maximum), the experimental setup, and the sensitivity of the fluorescent device can affect DR absolute values, and this should be considered by the user. Reliable quantitative measurements require a high signal-to-noise ratio, and we verified that this was the case in all our proof-of-concept assays. We also searched the images for spectroscopic artifacts, avoiding samples with “patches” that appeared to differ strongly in fluorescent readout between different areas of the same cell or organelle, thus contradicting general biophysical concepts on diffusion. We confirmed in our measurements that NERNST was uniformly distributed in the cytosol, stroma, matrix, etc., as reflected by homogeneous fluorescent readouts within a continuous space confined by a membrane. Also, the ratiometric nature of the sensor is expected to mitigate these inconveniences, except when the DR is too small to allow meaningful determinations.

Taken together, the results show that NERNST can monitor redox changes of the *E. coli* NADP(H) pool under various nutritional conditions. They also agree with the progressive increase of cellular NADPH levels from glucose to glycerol to acetate, as determined by liquid chromatography-mass spectrometry on exponentially growing *E. coli* cells^54^. To evaluate how precisely the biosensor estimates *E*_NADP(H)_ values, we determined the intracellular NADP^+^ and NADPH levels using the enzymatic cycling method^55^. This comparison is useful as a validation in this system because all NADP(H) is confined to a single cellular compartment. Redox potentials obtained using NERNST or the destructive assay showed a good correlation, with no statistically relevant differences (Fig. 2b).

NERNST fluorescence output most likely reflects the redox status of the free NADP(H) pool, while the extractive method estimates total NADP(H). A large fraction of this pool has been reported to be bound to cellular proteins in mammalian cells^56^, although little is known about the situation in bacteria. Then, the match between the two approaches might reflect a significantly higher fraction of free NADP(H) in *E. coli* compared to mammals, or a similar redox status in the free and bound pools. Further research will be necessary to elucidate this question.

### Determination of NADP(H) redox changes in the cytosol and chloroplasts of leaf cells

**Fig. 3:**
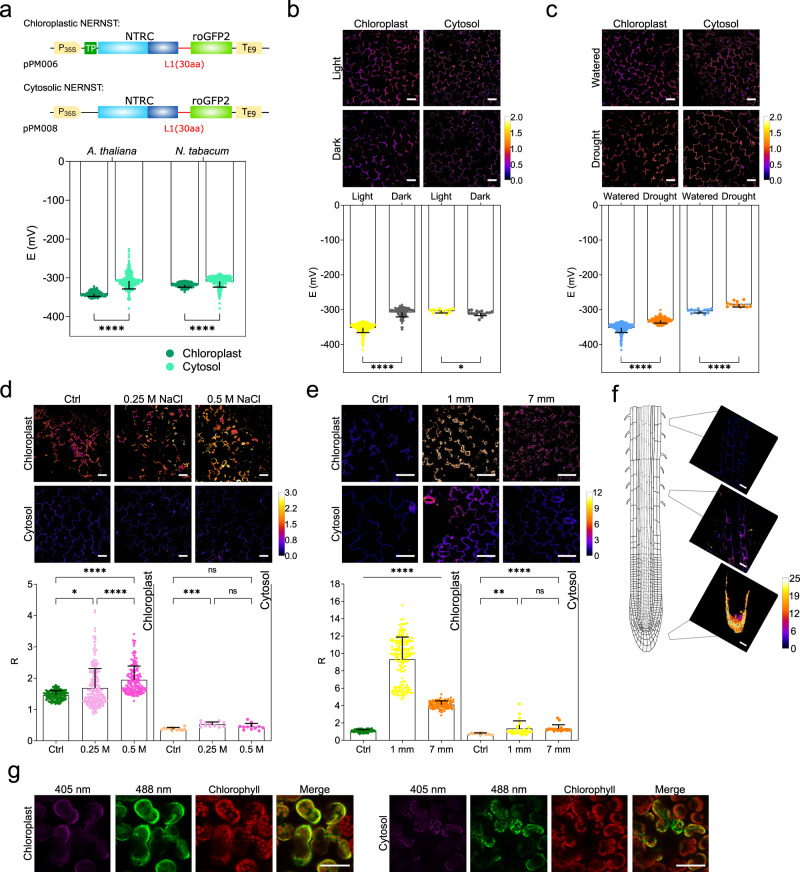
In vivo analysis of the NADP(H) redox state in the cytosol and chloroplasts of leaf cells and roots. **a** Top, constructs for stable NERNST expression in the cytosol or chloroplasts of plants under control of the *P*_*35S*_ promoter. TP: plastid-targeting sequence; L1: 30-aa linker. Bottom, estimation of *E*_NADP(H)_ in the chloroplasts and cytosol of illuminated Arabidopsis and *N. tabacum* leaf cells. Potentials were calculated assuming pH of 7.3 and 8.0 for cytosol and stroma, respectively^60^. Data shown are means ± SD of 131–253 cells. ****P ≤ 0.0001; two-way ANOVA, Bonferroni’s multiple comparisons test. **b** Light response of NERNST in tobacco leaf cells illuminated (Light) or not (Dark) at 200 μmol quanta m^−2^ s^−1^ for 3 h. *E*_NADP(H)_ values were calculated assuming stromal pH of 7.2 and 8.0 for dark and light conditions, respectively^61,62^. Data shown are means ± SD of 12-363 cells. *P ≤ 0.05, ****P ≤ 0.0001; two-tailed Mann–Whitney test (Chloroplast) or Unpaired *t* test (Cytosol). **c** Drought responses of NERNST in tobacco leaf cells. Excised leaves were air-dried for 3 h, prior to NERNST imaging. Data shown are means ± SD of 12-356 cells. ****P ≤ 0.0001; two-tailed Mann–Whitney test (Chloroplast) or Unpaired *t* test (Cytosol). **d** Salt responses of NERNST-expressing tobacco leaf discs exposed to the indicated concentrations of NaCl for 16 h. Data shown are means ± SD of 12-229 cells. *P ≤ 0.05, ***P ≤ 0.001, ****P ≤ 0.0001; ns, non-significant; one-way ANOVA, Dunn’s multiple comparisons test (Chloroplast) or Bonferroni’s multiple comparisons test (Cytosol). **e** NERNST imaging of tobacco leaves inoculated with *P. carotovorum* at two distances from the infiltration site. Data shown are means ± SD of 10-175 cells. **P ≤ 0.01, ****P ≤ 0.0001; ns, non-significant; one-way ANOVA, Dunn’s multiple comparisons test. **f** Ratiometric images (*R* values) of roots from 14-day-old tobacco plants grown in 0.5×MS-agar. **g** Transiently-expressed NERNST in the chloroplasts and cytosol of *N. benthamiana* leaf cells. Right panels: merge between 488-nm and chlorophyll fluorescence. Representative images and data shown were produced in at least two independent experiments. Scale bars, 50 μm. **b**–**f** Pseudocolor scales = R values. Source data, including all precise *n* values and exact *P* values, are provided as a Source Data file.

Monitoring NADPH levels in leaves is of the utmost importance because it provides the reducing power for the assimilation of CO_2_, which ultimately represents the main source of reduced carbon that shapes life on Earth as we know it. Most NADPH is produced in chloroplasts by the light reactions of photosynthesis, and consumed there in key biosynthetic reactions or exported to the cytosol to engage in other metabolic, protective or signaling pathways^57^, underscoring the importance of estimating NADP(H) levels in different cellular compartments, especially plastids. In order to provide NERNST access to specific organelles, we utilized a transit peptide (TP) as N-terminal extension added to the biosensor to permit chloroplast import and NADP(H) detection in the stroma. Constructs encoding plastid- and cytosol-targeted NERNST were placed under control of the constitutive cauliflower mosaic virus 35S promoter (*P*_*35S*_; Fig. 3a) and introduced in Arabidopsis and tobacco plants via *Agrobacterium*-mediated stable transformation^58,59^. While NERNST variants containing both the 30-aa and 45-aa linkers were readily expressed in the cytosol, only the 30-aa version could be imported by chloroplasts, and thus used in further experiments (Supplementary Table 2). Typical images of leaf cells from transgenic lines of the two species grown under chamber conditions are shown in Supplementary Fig. 15a–e. Plastid targeting was confirmed by co-localization with chlorophyll auto-fluorescence as chloroplast marker (Supplementary Fig. 15a, c). Cytosolic expression was visualized in both epidermal (Supplementary Fig. 15b, d) and mesophyll cells (Supplementary Fig. 15e). Transgenic plants displayed no obvious phenotypic alterations in development and reproduction due to expression of the biosensor (Supplementary Fig. 15f), allowing selection of homozygous lines.

Fluorescence imaging revealed uniform biosensor responses to H_2_O_2_ and DTT in both plastids and cytosol and in the expected directions (Supplementary Fig. 15a–d). Dynamic ranges were determined for both species (Supplementary Fig. 15g), and *R*_ox_/*R*_red_ values used to calculate the degree of oxidation of the reporter in each compartment, and from it the magnitude of *E*_NADP(H)_, assuming pH values of 7.3 and 8.0 for the leaf cytosol and stroma, respectively, under light conditions^60^. This sets a lower threshold for *E*_NADP(H)_ detection of −338 mV in the cytosol and −380 mV in chloroplasts. As expected, the NADP(H) pool was significantly more reduced in chloroplasts than in the cytosol for both species (Fig. 3a).

Since NADPH is produced and consumed during photosynthesis, the response of the biosensor was studied in dark-adapted and illuminated tobacco leaves. Stromal *E*_NADP(H)_ decreased from −308 mV in the dark to −352 mV in the light (Fig. 3b), assuming a pH shift of 7.2 to 8.0 upon illumination^61,62^. Average light *E*_NADP(H)_ values estimated an NADPH/NADP^+^ ratio of ~1.5, which agrees well with previous reports for barley chloroplasts^63^. Determination of *E*_NADP(H)_ for the cytosolic NADP(H) pool indicates that it was similar to that of dark-adapted chloroplasts, and slightly more positive (oxidized) in the light than in the dark (Fig. 3b). Using iNap bioprobes specific for NADPH, Lim et al.^57^ showed that the reduced form of the dinucleotide increased in the stroma but decreased in the cytosol during dark-light transitions of Arabidopsis seedlings, in line with the observations reported here. Similar results have been obtained in rapid fractionation experiments^64^, but not assigned any particular relevance in physiological terms.

NADP^+^ and NADPH contents were also determined by extractive methods in whole extracts from illuminated and dark-adapted leaves. Results obtained with both tobacco and Arabidopsis showed a trend similar to those reported by NERNST in chloroplasts, namely, a redox shift toward reduction of the NADP(H) pool upon dark-light transition (Supplementary Fig. 16). It is not possible to advance in comparison with NERNST-derived data because the NADPH/NADP^+^ ratios obtained by this procedure reflect the contributions of all cellular compartments (including mitochondria, peroxisomes, etc.). They cannot thus be used to calculate redox potentials, nor can they be quantitatively compared to the ratios or potentials estimated in specific locations such as chloroplasts or cytosol. This analysis illustrates the shortcomings of destructive methods compared to genetically-encoded biosensors in monitoring the space- and time-resolved dynamics of redox couples in vivo.

Stress situations known to trigger oxidative stress, such as drought, led to a significant increase in *E*_NADP(H)_, especially in chloroplasts (Fig. 3c). The R values, per se, provide a comparative estimation of the NADP(H) redox status in those cases in which R_ox_ and R_red_ cannot be determined^53^, due to poor access of oxidants and reductants and/or excess damage to the tissue. Moreover, if the change to be analyzed does not involve a pH shift, the R readouts directly reflect the differences in redox state between the initial and final conditions. This possibility is illustrated for NERNST-expressing leaves exposed to salt (Fig. 3d) and biotic stress (Fig. 3e). As in the case of drought, the redox shift caused by salinity predominantly affected the chloroplast NADP(H) pool (Fig. 3d). Biotic stress, in turn, was assayed by inoculating leaves with the necrotrophic bacterium *Pectobacterium carotovorum*, which elicits an oxidative burst that uses NADPH as electron donor^65^. Accordingly, NERNST indicated significant NADPH oxidation in the proximity of the infection site (Fig. 3e). Data points are distributed in two apparent populations at the 1-mm sampling site (Fig. 3e), likely reflecting the cellular heterogeneity in terms of both fluorescence output and biotic damage, which is particularly critical in this region close to the infection source. Oxidation of the chloroplast NADP(H) pool declined at longer distances, whereas the cytosolic redox status remained unchanged (Fig. 3e).

Constitutive expression driven by the *P*_35S_ promoter allowed visualization of the probe in various tissues including roots. Space-resolved imaging of the NADP(H) redox state along an axis longitudinal to the tobacco root length revealed that the pyridine nucleotide pool became more oxidized when approaching the root tip (Fig. 3f). This observation concurs with previous reports showing that both the root cap and the columella have increased levels of reactive oxygen species (ROS) and redox potential^66^.

The NERNST-encoding construct was also amenable to transient expression methods such as those developed for *Nicotiana benthamiana* leaves, displaying high levels of fluorescence in both chloroplasts and cytosol (Fig. 3g), and providing a rapid tool to estimate *E*_NADP(H)_ in different cellular compartments without the time investment needed for stable transformation.

### NERNST probes the NADP(H) redox status in isolated leaf protoplasts

Plant protoplasts display features that make them attractive as an experimental model system: they retain important properties of the original cells, can be easily isolated from mutant or transgenic plants, and can be transformed in vitro leading to transient expression of genes of interest. Moreover, the removal of the cell wall makes them accessible to pharmacological approaches which are impaired in whole tissues, and opens their adaptation to mid-throughput experimental set-ups^67,68^. Accordingly, they represent valuable tools to investigate biochemical and physiological processes in a fast and reliable way, and it was therefore of considerable interest to evaluate the behavior of NERNST in this system.

**Fig. 4:**
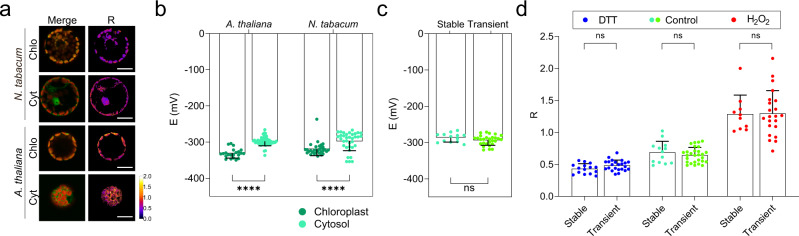
NERNST probes the NADP(H) redox status in isolated leaf protoplasts. Expression vectors used are those described in Fig. 3a. **a** Representative fluorescence images of NERNST in protoplasts isolated from Arabidopsis and tobacco leaves expressing the biosensor in chloroplasts (Chlo) or the cytosol (Cyt). Panels show merge between 488-nm and chlorophyll fluorescence, and R imaging. Scale bars, 20 μm. Pseudocolor scale = *R* values. **b** NADP(H) redox potentials (*E*) were estimated assuming pH values of 7.3 and 8.0 for the cytosol and stroma, respectively^60^. ****P ≤ 0.0001; two-way ANOVA followed by Bonferroni’s multiple comparisons test. **c**, **d** Comparison of cytosolic *E*_NADP(H)_ (**c**) and dynamic ranges including controls (**d**) between isolated Arabidopsis protoplasts stably and transiently expressing NERNST. ns, non-significant. Data are shown as means ± SD of 22-50 cells (**b**), 13–28 cells (**c**), 10-28 cells (**d**). Data were analyzed by two-way ANOVA followed by Bonferroni’s multiple comparisons test (**b**, **d**), two-tailed Unpaired *t* test (**c**). Source data, including all precise *n* values and exact *P* values, are provided as a Source Data file.

Protoplasts isolated from leaves of Arabidopsis and tobacco plants stably transformed with the biosensor displayed typical signals in both chloroplasts and cytosol (Fig. 4a). Exposure to H_2_O_2_ or DTT elicited fluorescence changes in the expected directions for the two species (Supplementary Fig. 17a–d). Estimations of *E*_NADP(H)_ for the chloroplast stroma and cytosol yielded results comparable to those obtained in whole leaves (Fig. 4b).

Transient expression of NERNST in Wt Arabidopsis protoplasts using the vectors employed for stable transformation yielded essentially the same type of fluorescent images, as exemplified for the cytosolic reporter (Supplementary Fig. 17e). *E*_NADP(H)_ values (Fig. 4c) and dynamic ranges (Fig. 4d) showed good correlations between stably and transiently transformed protoplasts, suggesting that the manipulations involved in protoplast transformation did not cause major shifts in the NADP(H) redox balance. The results indicate that NERNST can be employed to estimate *E*_NADP(H)_ in experiments using the protoplast model system.

### Monitoring NADP(H) dynamics in cultured human cells and mitochondria

**Fig. 5:**
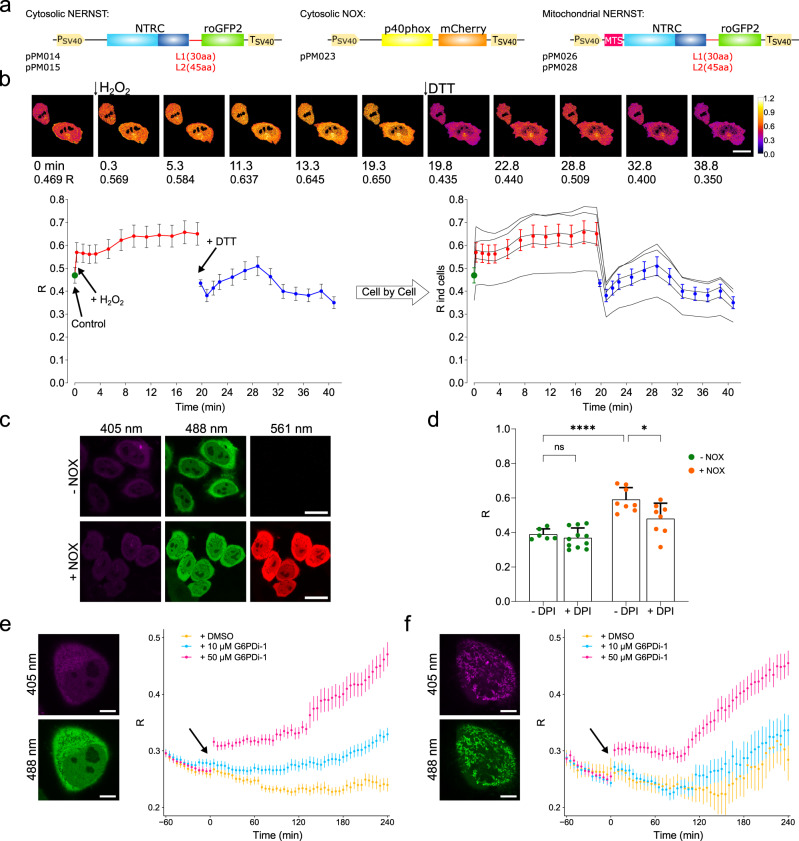
Mid-throughput analysis of NADP(H) dynamics in mammalian cells and mitochondria. **a** Construct design for expression of NERNST and the p40phox subunit of NADPH oxidase fused to the fluorescent marker mCherry (NOX) in the cytosol and mitochondria of mammalian cells. NERNST was placed under control of the SV40 promoter in pPM014/15, and the mitochondrial targeting sequence (MTS) for mitochondrial localization in pPM026/28. L1: 30-aa linker. L2: 45-aa linker. **b** Spatio-temporal resolution of fluorescence R responses of the NERNST biosensor expressed in HeLa cells to successive additions of 10 mM H_2_O_2_ and 10 mM DTT, as shown in sequential frames (above), time-course and *R* values (below). Scale bars, 20 μm. Average (left) and cell-by-cell (right) analyses are shown in the lower part of the panel. Time 0 (green dots) indicates cells without treatments. Data shown are means ± SEM of 5 cells. Pseudocolor scale = *R* values. **c** Representative fluorescence images of NERNST (405 nm and 488 nm) co-expressed (bottom) or not (top) with NOX (561 nm). Scale bars, 20 μm. **d** NOX-mediated oxidation of the NADP(H) pool was abolished by the addition of 50 μM DPI. Data shown are means ± SD of 6-11 cells. *P ≤ 0.05, ****P ≤ 0.0001, ns, non-significant; two-way ANOVA followed by Tukey’s multiple comparisons test. **e**, **f** Kinetics of NERNST fluorescence responses in the cytosol (**e**) or mitochondria (**f**) of HeLa cells upon the addition of dimethyl sulfoxide (DMSO; control), 10 μM or 50 μM of the G6PDH inhibitor G6PDi-1, as determined with the PerkinElmer Operetta multi-well plate format confocal microscope. Data shown are means ± SEM of 7-98 cells. Representative images of cells expressing NERNST in cytosol (**e**) and mitochondria (**f**) are shown on the left. Scale bars, 10 μm. Source data, including all precise *n* values and exact *P* values, are provided as a Source Data file.

To monitor NADP(H) in animal systems, we first expressed NERNST in the cytosol of cultured human embryonic kidney (HEK)-293T and HeLa cells by transfection with vectors expressing the biosensor under control of the constitutive SV40 promoter (Fig. 5a). Uniform and intense fluorescence was detected in cells of the two lines (Fig. 5b, c; Supplementary Fig. 18a–c). Images were analyzed after various treatments. Addition of a bolus of H_2_O_2_ or DTT caused fluorescence changes in the expected directions (Fig. 5b; Supplementary Fig. 18a). Use of the thiol oxidizing agent diamide^16^ yielded similar results (Supplementary Fig. 18a, b). Fluorescence changes driven by exposure to H_2_O_2_ displayed a biphasic response, with a fast increase in R upon addition of the oxidant, followed by a second, slower rise after a lag period of several minutes (Fig. 5b). Subsequent addition of DTT led to rapid reduction (Fig. 5b). HEK-293T cells behaved in a similar way (Supplementary Fig. 18c). Comparison of initial R values in untreated cells with R_ox_ and R_red_ indicates that NERNST was largely reduced prior to the addition of the oxidant (Fig. 5b; Supplementary Fig. 18c).

We then used the sensor to report the variations in intracellular NADP(H) redox status caused by two different types of perturbations: genetic and pharmacological. First, the NADP(H) redox poise of HeLa cells was altered by expressing the p40phox subunit of human NADPH oxidase, which promotes O_2_ reduction at the expense of NADPH consumption^69^. The oxidase subunit was fused to the fluorescent marker mCherry^70^ (Fig. 5a) for cellular visualization of the transgenic product (NOX), and indeed, strong red fluorescence could be detected in the co-transfected cells (Fig. 5c). Image analysis of the NERNST reporter showed that the NADP(H) pool became more oxidized upon NOX expression (Fig. 5d). Addition of the NOX inhibitor diphenyleneiodonium (DPI) to the cell suspension prevented NADPH oxidation in NOX-expressing cells (Fig. 5d), indicating that the effect was dependent on the activity of the oxidase. Finally, NOX had no effect on the R values displayed by HeLa cells expressing roGFP2 or the inactive Cys-to-Ser mutant of NERNST (Supplementary Fig. 19).

G6PDH is the main source of cytosolic NADPH in resting HeLa cells^16^. Incubation of the cells with the G6PDH inhibitor G6PDi-1 (ref. ^71^) in high-glucose medium is expected to impair NADP^+^ reduction, with concomitant oxidation of the NADP(H) pool. We monitored the NERNST fluorescence changes using the Operetta^64^ high-content confocal imaging system which allows mid-throughput parallel and fast analysis of set-ups in multi-well format. Addition of G6PDi-1 led to a significant increase in the cytosolic R values of NERNST in a time- and concentration-dependent manner (Fig. 5e). The time-course of NADP(H) oxidation shows a lag phase with little or no change in R values, presumably reflecting the time required to progressively deplete the NADPH pool by consuming pathways, under conditions of limited replenishment through NADPH-producing enzymes other than G6PDH.

In animals, the impact of mitochondrial oxido-reductive metabolism on physiology, disease and aging is well recognized^72^. Given its importance, we targeted NERNST to HeLa mitochondria to monitor the NADP(H) redox state, by fusing the mitochondrial targeting sequence of subunit 8 from the human cytochrome *c* oxidase complex to the N-terminus of the biosensor (Fig. 5a). As in chloroplasts, the biosensor carrying the 45-aa linker did not accumulate in mitochondria. In this case, however, significant fluorescence was observed in the cytosol (Supplementary Fig. 20), suggesting that this NERNST precursor could not be imported by the organelle. Instead, NERNST fluorescence was readily detected in the mitochondria of cells transfected with the biosensor carrying the 30-aa linker (Fig. 5f, left). The mitochondrial NADP(H) pool oxidized spontaneously (i.e., in the absence of G6PDi-1) after prolonged incubation in the LCIS solution used for imaging (see Methods). Oxidation was not caused by the dimethyl sulfoxide used as solvent for G6PDi-1 (Supplementary Fig. 21) and most likely relates to the nutrient paucity of the imaging medium, which might boost mitochondrial oxidative metabolism after prolonged incubation. Exposure to G6PDi-1 led to further oxidation of the mitochondrial NADP(H) pool, again showing a two-phase kinetics (Fig. 5f). It is not clear if this effect results from direct or secondary causes. Several pathways have been implicated in the shuttling of reducing equivalents between the cytosol and mitochondria^7,56^, and might explain the observed correlation in the responses of the two compartmentalized pools. It is also possible, however, that G6PDi-1 could inhibit the mitochondrial isoform of G6PDH, which is regarded as the main source of NADPH in the organelle^73,74^.

The collected results indicate that NERNST could be expressed and folded in a functional conformation in the cytosol and mitochondria of cultured mammalian cells, providing a reliable tool to monitor NADP(H) dynamics in different cellular compartments of the host cell. In combination with mid-throughput microscopy, the sensor can therefore be implemented as a versatile platform for pharmacological and genetic studies of metabolism and physiology dynamics in animal cells, with important applications in the biomedical field since perturbations of NADP(H) homeostasis have been associated with several pathological conditions^2–4,75^.

### Detection of NADP(H) changes upon injury responses in zebrafish larvae

**Fig. 6:**
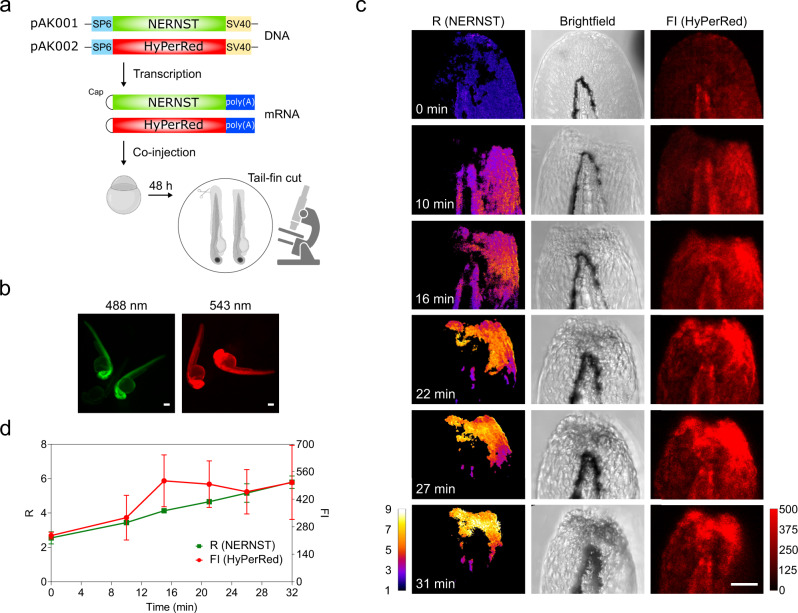
Simultaneous imaging of NADP(H) and peroxide dynamics in a wounded region of zebrafish tail fin. **a** Experimental set-up to evaluate NADP(H) redox status and ROS levels in zebrafish embryos after wounding. The upper part shows the constructs used for NERNST and HyPerRed expression, cloned into the pCS2+MT vector system between the SP6 promoter and SV40 terminator and in vitro transcribed from the corresponding linearized plasmids (pAK001/2). The mixed mRNAs were microinjected in 1-cell zebrafish embryos (scheme created with BioRender.com). **b** Visualization of NERNST (left) and HyPerRed (right) fluorescence in zebrafish specimens was carried out in each of 3 independent experiments at 48 h post-fertilization using an Olympus MVX10 Macro Zoom fluorescence microscope. Emission of NERNST was monitored at 510 nm after excitation at 488 nm, whereas the HyperRed sensor was excited at 543 nm and recorded at 610 nm. Representative images are shown. Scale bars, 50 μm. **c** Time-resolved NADP(H) and peroxide dynamics in wounded margins of the tail fin tip. From left to right, *R* (NERNST), brightfield and Fluorescence Intensity (FI; HyPerRed). Times after wounding are shown in the *R* panels. Scale bar, 50 μm. Pseudocolor scale = *R* values. Red scale = HyPerRed Fluorescence Intensity. (**d**) Kinetics of NADP(H) and peroxide changes. Green line, *R* values of NERNST; red line, HyPerRed fluorescence. Data in (**d**) are means ± SD of 2 (NERNST) or 3 (HyPerRed) independent measurements. Source data are provided as a Source Data file.

Wounding of zebrafish larvae leads to a localized oxidative burst and a concurrent decline of NADPH levels^16^. We therefore probed whether NERNST can detect these changes in NADP(H) redox dynamics in vivo. DNA sequences encoding NERNST and HyPerRed, a fluorescent H_2_O_2_ sensor^76^ were cloned in pCS2+MT vectors between the SP6 promoter and SV40 terminator (Fig. 6a). Messenger RNAs encoding NERNST and HyPerRed were separately synthesized, mixed, and microinjected in 1-cell staged zebrafish embryos for simultaneous visualization of the NADP(H) redox state and H_2_O_2_ levels (Fig. 6a). Strong fluorescence from NERNST and HyPerRed, green and red respectively, was detected 48 h after mRNA co-microinjection (Fig. 6b). Then, the tip of the tail fin was amputated (Fig. 6a) and ROS build-up at the injury region was monitored using HyPerRed. Sensor fluorescence increased and spread up to 15 min, then declined in intensity and began to recede (Fig. 6c).

NERNST R values also increased after fin wounding, indicating oxidation of the NADP(H) pool (Fig. 6c). The time course was similar to that of HyPerRed except that NADPH oxidation proceeded unabated during the 31-min timeframe of the assay (Fig. 6d). Using the NADPH biosensor iNap, Tao et al.^16^ reported a decline in NADPH levels upon wounding which is consistent with the decrease in the NADPH/NADP^+^ ratio observed here, indicating that NERNST can be employed to measure the NADP(H) redox status in living zebrafish embryos.

## Discussion

We describe herein the design and characterization of NERNST, a ratiometric biosensor for monitoring NADP(H) redox state in living cells, based on the fusion of NADP(H)-dependent NTRC^27^ to redox-sensitive roGFP2^21^. Purified NERNST was able to reversibly react with NADPH and NADP^+^, and was highly specific for the NADP(H) pair (Fig. 1; Supplementary Figs. 7–9). Under the conditions employed, full reduction and oxidation was completed in a few minutes (Supplementary Figs. 5–9), providing a measure of the time responsiveness of the probe. The NADP(H) reporter could be expressed in many different cell types and targeted to chloroplasts and mitochondria, allowing detection of changes in NADP(H) redox poise in bacteria (Fig. 2), leaf and root cells in whole plants and mesophyll protoplasts (Figs. 3, 4), mammalian cultured cells (Fig. 5) and live fish embryos (Fig. 6).

NERNST shares many favorable properties with former roGFP-based reporters^25,26,29,77^, including intense fluorescence emission, ratiometric behavior and activity in heterologous biological systems. NERNST is largely insensitive to pH variations within physiological margins (Fig. 1f). Changes in fluorescence intensities can be followed by confocal microscopy and microplate reading, as done here, and potentially also by flow cytometry. When expressed in living organisms, sensors based on roGFP may interact, in principle, with multiple redox partners, although kinetic limitations are expected to exist for most of them as the electron transfer is normally dominated by the fused partner/primary electron acceptor^26,40,41^. In the case of NERNST, the NADP(H)-specific NTRC module and roGFP2 are forced into close proximity in the fusion construct, favoring the kinetics of redox exchange between them over other possible competitive reactions, thus allowing the use of NERNST as a tool to monitor NADP(H) redox status in living organisms, within the timeframe and pH range reported. Indeed, proof-of-concept experiments designed to modify the NADP(H) status by genetic interventions, environmental challenges and different nutritional sources indicated that the biosensor effectively reflects the expected changes in the redox state of the NADP(H) pool (Figs. 2–6).

NERNST does not measure absolute NADP(H) concentrations, estimating instead its oxido-reductive status, a most relevant physiological parameter to investigate metabolic dynamics and predict cellular responses to environmental, developmental and genetic inputs. Indeed, the NADP(H) redox status is a key player in physiology, development and defense of all living organisms, with profound implications for biology and medicine. So far, estimations of this all-important parameter have relied on the use of destructive methods which cannot discriminate between cellular compartments and display significant reproducibility shortcomings. Genetically-encoded bioprobes have been designed to detect absolute levels of either NADP^+^ or NADPH but not their ratio, precluding estimation of the NADP(H) redox poise. NERNST overcomes this limitation and allows monitoring in vivo NADP(H) dynamics in a direct and reliable way, thus providing a useful tool to investigate the role of this central metabolite in growth, disease and responses to environmental stimuli. Moreover, it can be combined, in principle, with sensors based on a binding mechanism such as Apollo-NADP^+^ and iNap^16,17^, which can be spectrally tuned for multi-parametric imaging^78^.

The genetically-encoded biosensor proved to be versatile, as exemplified by its functionality in a series of evolutionary distant organisms (bacteria, plants and animals), different subcellular localizations (cytosol, mitochondria, chloroplasts) and in combination with nutritional, metabolic, pharmacological and genetic perturbations. It is readily customizable for use both in simple set-ups available in standard biochemistry/molecular biology labs and in mid-throughput experimental approaches employing advanced confocal microscopy set-ups. Generalized application of NERNST in biochemical, biotech and medical research will open up novel perspectives to study the contribution of NADP(H) in such fundamental biological processes as embryogenesis, aging, inflammation and cell death, tissue regeneration and disease responses.

## Methods

### Ethical Statement

The study used a zebrafish AB line provided by the IBR-CONICET Acuario®, Rosario, Argentina. Animals were handled in compliance with relevant international guidelines (Policy on Humane Care and Use of Laboratory Animals and American Veterinary Medical Association, https://zfin.org/). The CICUAL (Comité Institucional para el Cuidado y Uso de Animales de Laboratorio; Institutional Committee for the Care and Use of Laboratory Animals) from the University of Rosario, Argentina (Facultad de Ciencias Bioquímicas y Farmacéuticas – Universidad Nacional de Rosario—UNR), approved and monitored the research protocol involving zebrafish (Res. N° 207/2018). To obtain embryos, male and female specimens were mated in a 3:4 ratio. The study did not involve wild animals or samples collected from the field.

### Design of the NADP(H) ratiometric sensor (NERNST) and plasmids construction

DNA fragments coding for rice NADPH-thioredoxin reductase C (*NTRC*)^28^ and roGFP2^21^ were amplified from previously described expression vectors^28,52^ (see Supplementary Table 1) with primers oHB272/oHB273 for NTRC and oHB274/oHB275 for roGFP2 (Supplementary Table 3). The NERNST biosensor was generated by fusing the DNA sequence coding for roGFP2 downstream of *NTRC*. In-frame sequences encoding 30- or 45-amino acid (aa) linkers containing Gly, Ser and Ala in various arrangements (Supplementary Table 2) were introduced between NTRC and roGFP2 via Fusion-PCR to probe different geometries for electron exchange. Constructs were cloned into plasmid pWW301 digested with NdeI/HindIII using Gibson Cloning^79^ to generate pHB124 and pHB125 expression plasmids (Supplementary Table 1). Linker-encoding sequences were synthesized by gBlocks (Integrated DNA Technologies, Leuven, Belgium). Mutant versions of the biosensor (in pHB129 and pHB130) were prepared by simultaneously replacing functional cysteines by serines at positions C140, C143, C377 and C380 of NTRC (Supplementary Table 1), using PCR amplification and Gibson assembly with primers oHB181/oHB182 and oHB183/oHB184 (Supplementary Table 3).

### Expression and purification of recombinant proteins

NERNST DNA sequences were obtained from the corresponding pHB plasmids by digestion with NdeI and HindIII, and cloned in pET-TEV under the control of the T7 promoter to yield pPM002, pPM003, pPM004 and pPM005 (Supplementary Table 1). These plasmids were used to transform *Escherichia coli* BL21(DE3). To improve folding of the expressed proteins and recovery in the soluble fraction, cells were co-transformed with plasmids expressing various molecular chaperones under the control of the *araB* promoter (Supplementary Table 1, Takara Bio Inc, Kusatsu, Japan). Bacteria were grown in Luria-Bertani (LB) broth supplemented with 40 µg/ml kanamycin and 25 µg/ml chloramphenicol at 37 °C for 5 h and induced overnight at 20 °C by the addition of 50 μM IPTG and 0.5 mg/ml L-arabinose. Cells co-transformed with plasmid pGRO7 expressing GroEL and GroES yielded the highest NERNST levels and were used thereafter. Bacteria were lysed by sonication, and cleared lysates prepared by centrifugation. Recombinant proteins were bound to a Ni-NTA column (Qiagen, Hilden, Germany) through the His-tag, eluted with 0.5 M imidazole and then dialyzed against 100 mM K_3_PO_4_ pH 7.3, 1 mM EDTA, 150 mM NaCl. Eluates were analyzed by SDS- and native PAGE; the latter showed two major fluorescent bands corresponding to dimeric and monomeric forms. The dimer was purified by molecular exclusion chromatography (UNICORN Control System 1 software) on a Superdex-200 Increase 10/300GL column (GE Healthcare Life Sciences, Chicago, IL) developed in 100 mM K_3_PO_4_ pH 7.3, 1 mM EDTA, 150 mM NaCl, and used for in vitro experiments.

### In vitro characterization of NERNST

Absorption and fluorescence spectra of purified NERNST dimers were recorded before and after oxidation by 10 mM H_2_O_2_ or reduction with 10 mM DTT in 100 mM K_3_PO_4_ pH 7. Relative quantum yields were determined as described by Würth et al.^80^, using fluorescein (Sigma Aldrich St. Louis, MO) as standard. In brief, the absorption spectra of oxidized or reduced NERNST in 100 mM K_3_PO_4_ pH 7 and fluorescein in 0.1 N NaOH were recorded to identify a common isosbestic point of 471 nm. Concentrations of samples and standard were adjusted to yield the same absorbance at the isosbestic wavelength, which was used for excitation. Emission spectra were measured in a Cary Eclipse fluorescence spectrophotometer (Cary Eclipse software) with identical instrument settings for probes and standard, after excitation at 471 nm. Since the refractive index of the solutions and the absorbance at the excitation wavelengths were the same for samples and standard, the relative quantum yields of the samples (Φ_f,s_) were determined using the following equation^80^:

1 $${\Phi }_{{{{{{\rm{f}}}}}},{{{{{\rm{s}}}}}}}={\Phi }_{{{{{{\rm{f}}}}}},{{{{{\rm{st}}}}}}}\frac{{{{F}}}_{{{{{{\rm{s}}}}}}}}{{{{F}}}_{{{{{{\rm{st}}}}}}}}$$

where Φ_f,st_ is the quantum yield of the fluorescein standard (st) under these conditions (0.89)^80^, and *F*_s_ and *F*_st_ are the fluorescence emission of sample and standard, respectively.

To assay the redox properties of purified NERNST, dimers were incubated with 10 mM H_2_O_2_, 10 mM DTT or 0.5 mM of either NADPH, NADH or GSH in 100 mM K_3_PO_4_ pH 7. Fluorescence emission intensities of NERNST (*I*) were measured at 528/20 nm in a fluorimetric plate reader (Synergy 2 Biotek, Himex, Turku, Finland; Gen5 software) after excitation at 360/40 nm or 485/20 nm to determine the R parameter (*R* = *I*_390_/*I*_490_), using 90-µl solutions of the recombinant protein. The wavelengths correspond to those provided by the equipment, and their exact values will vary depending on the availability of filters in each fluorescence detection system, as indicated below. The pH response of the biosensor was assayed between 5.8–8.0 in a buffer containing 0.2 M NaH_2_PO_4_ and 0.2 M Na_2_HPO_4_.

For the determination of midpoint redox potentials, the recombinant dimer was mixed with different NADPH/NADP^+^ ratios at final concentrations of 0.5 mM or 1 mM total NADP(H). Fully oxidized and reduced samples were prepared by reaction with 10 mM H_2_O_2_ and 10 mM DTT under the same conditions. After 7 min of incubation at 25 °C, *I*_390_ and *I*_490_ were recorded and employed to calculate the degree of oxidation of the biosensor (*OxD*_NERNST_) using (Eq. 2) (ref. ^25,26,52^.). The *OxD*_NERNST_ values measured under each condition allowed determination of *E*_NERNST_ by the Nernst equation (Eq. 3), and *E*_NADP(H)_, assuming that the two potentials are equal at detection (Eq. 4).

2 $${{OxD}}_{{{{{{{\rm{NERNST}}}}}}}}=\frac{R-{R}_{{red}}}{\frac{{I}_{490\min }}{{I}_{490\max }}({R}_{{ox}}-R)+(R-{R}_{{red}})}$$

3 $${E}_{{{{{{{\rm{NERNST}}}}}}}}={{E}^{0{\prime} }}_{{{{{{{\rm{NERNST}}}}}}}}-\frac{{RT}}{{nF}}{{{{\mathrm{ln}}}}}\frac{\left({1-{OxD}}_{{{{{{{\rm{NERNST}}}}}}}}\right)}{\left({{OxD}}_{{{{{{{\rm{NERNST}}}}}}}}\right)}$$

4 $${E}_{{{{{{{\rm{NADP}}}}}}}\left(H\right)}={{E}^{0{\prime} }}_{{{{{{{\rm{NADP}}}}}}}\left(H\right)}-\frac{{RT}}{{nF}}{{{{\mathrm{ln}}}}}\frac{\left[{{{{{{\rm{NADPH}}}}}}}\right]}{\left[{{{{{{{\rm{NADP}}}}}}}}^{+}\right]}={{E}^{0{\prime} }}_{{{{{{{\rm{NERNST}}}}}}}}-\frac{{RT}}{{nF}}{{{{\mathrm{ln}}}}}\frac{\left({1-{OxD}}_{{{{{{{\rm{NERNST}}}}}}}}\right)}{\left({{OxD}}_{{{{{{{\rm{NERNST}}}}}}}}\right)}={E}_{{{{{{{\rm{NERNST}}}}}}}}$$

The term *R* in Eq. (2) is the *I*_390_/*I*_490_ ratio for NERNST under any given condition, with the subscripts “red” and “ox” indicating minimal and maximal R values measured with completely reduced and oxidized NERNST, respectively. *I*_490min_ and *I*_490max_ refer to the fluorescence intensities measured with excitation at 490 nm for fully oxidized and fully reduced NERNST. In Eqs. (3) and (4), *R* is the gas constant (8.314 J/mol.K), whereas *T* is the absolute temperature at which the assays were conducted (~298 K), F is the Faraday constant (96,485 C/mol) and *n* is the number of electrons involved in the exchange (2). Standard redox potentials (*E°’*) used were −320 mV for NADP(H) and −280 mV for NERNST^22,25^ at pH 7.

Kinetic measurements were carried out in a Cary Eclipse fluorescence spectrophotometer after excitation at 390 nm and 490 nm. Emission was recorded at 510 nm. Dimeric NERNST responses to 0.5 mM NADPH alone or in the presence of 0.5 mM of either cysteine, GSH, GSSG, NADH or NAD^+^ were assayed in 100 mM K_3_PO_4_ pH 7. Dimeric NERNST oxidation by H_2_O_2_ in the presence of NADPH was measured in the same reaction medium. The biosensor was initially reduced by 0.5 mM NADPH and then exposed to successive additions of H_2_O_2_ (0.015–29 mM). Monomeric NERNST was tested in the same medium with 0.5 mM NADPH or 10 mM DTT.

For oxidation experiments with NADP^+^, dimeric NERNST was initially reduced by 10 mM DTT for 6 min on ice, and the fully reduced protein was desalted with Micro Bio-Sin® 6 Chromatography Columns (BioRad). Oxidation was carried out with 0.5-1 mM NADP^+^, 10 µM thioredoxin-A from *Escherichia coli* or 10 µM thioredoxin-*h* from peach, and the kinetics was followed in a Cary Eclipse fluorescence spectrophotometer after excitation at 390 and 490 nm. To test the oxidation of the biosensor by NADP^+^ in the presence of NADPH, dimeric NERNST was incubated with 0.25 mM glucose-6-phosphate (G6P), 1 unit of glucose−6-phosphate dehydrogenase (G6PDH) and the indicated concentrations of NADP^+^. Fluorescence was recorded at 6 min in the Synergy plate reader with the corresponding setup.

### Growth of *E. coli* in rich and minimal media

*E. coli* BL21 DE3 cells expressing NERNST were cultured in LB broth or M9 minimal medium^81^, in the latter case supplemented with 0.2% (w/v) casaminoacids and either 0.5% (w/v) glucose, 1% (v/v) glycerol or 1.2% (w/v) acetate as carbon sources. Antibiotics (40 μg/ml kanamycin and 25 μg/ml chloramphenicol) were added to all media. Induction with IPTG and L-arabinose was carried out as described before, except that the temperature was set at 28 °C instead of 20 °C after the addition of the inducer for bacteria grown in minimal media. Cells were harvested by centrifugation to quantify NADPH by redox cycling or used for confocal imaging after loading 10 μl of the bacterial suspension on a slide.

### Transformation of *Nicotiana* and Arabidopsis plants

The complete sequence encoding NERNST was PCR-amplified and cloned into the SacI/SalI sites of the plant binary vector pCHF3 under control of the constitutive cauliflower mosaic virus 35S promoter (*P*_*35S*_, Supplementary Table 1). The cytosolic version (pPM008) was amplified from pPM002 with primers oAK001/oAK002 (Supplementary Table 3). For chloroplast targeting, the biosensor-encoding DNA was amplified with the same primers and fused in-frame to the 5’ end of a 122-bp sequence encoding the transit peptide (TP) of pea ferredoxin-NADP^+^ reductase in plasmid pCHF3-TP-FLV1^82^, from which the FLV1 sequence had been removed, to yield pPM006. These plant vectors were used to transform *Agrobacterium tumefaciens* GV3101 PMP90 by electroporation and selection with 100 μg/ml spectinomycin in LB-agar plates. Stable transformations of *Nicotiana tabacum* cv. Petit Havana and *Arabidopsis thaliana* ecotype Columbia (Col-0) were carried out by published procedures^58,59^. In the case of tobacco, recombinant plasmids were introduced into the plant genome through *Agrobacterium*–mediated leaf disc transformation^58^. Discs were immersed in liquid culture of transformed Agrobacterium, and co-cultivated in Murashige and Skoog (MS) 0,8% agar supplemented with 1 µg/ml kinetin (Sigma) and 0.1 µg/ml naftalen acetic acid (Sigma) (regeneration medium) for two days^83^. At the end of this period, discs were transferred to the regeneration medium supplemented with 50 µg/ml cefotaxime to kill the bacteria and 100 µg /ml kanamycin to select the transformed plants. After two months the rooted plantlets were transferred to soil. Homozygous lines were selected by following kanamycin resistance in the progeny of self-pollinated primary transformants up to the T3 generation. Seeds were germinated on half-strength MS (0.5 × MS) agar plates supplemented with 2% (w/v) sucrose and 50 µg/ml kanamycin. Arabidopsis plants were transformed using the floral dip procedure^59^. Plants were grown until flowering, and the main inflorescence was cut to induce lateral branching and favor transformation by dipping the cut inflorescences in *Agrobacterium* cultures. T1 seeds were collected after 30/45 days. The selection of transformants was also conducted in 0.5 × MS-agar plates without sucrose and supplemented with 50 µg/ml cefotaxime, 100 µg /ml kanamycin, and followed up to the T3 generation to obtain homozygous lines. Two cytosolic lines exhibiting good expression levels (101-3-3 and 101-9-10 of tobacco, 101-2-4 and 101-2-6 of Arabidopsis) and two chloroplast-targeted lines (99-3-2 and 99-7-1 of tobacco, 99-4-6 and 99-4-11 of Arabidopsis) were used throughout this work. Tobacco plants were grown at 200 μmol quanta m^−2^ s^−1^, 16-h photoperiod and 25 °C (tobacco chamber conditions), whereas Arabidopsis plants were cultured at 135 µmol quanta m^−2^ s^−1^, 8-h photoperiod and 22 °C/18 °C (Arabidopsis chamber conditions).

Transient transformation of *Nicotiana benthamiana* was carried out by infiltrating the youngest fully expanded leaves of 22-day old plants^84^ using *A. tumefaciens* strain EHA105 carrying the pCHF3-derived vectors described above. Plants were grown under tobacco chamber conditions. Transiently expressed products showed maximum fluorescence at 72 h, as revealed by microscopic inspection.

### Stress treatments on tobacco plants expressing NERNST

The youngest fully expanded leaves of soil-grown 7-week-old tobacco plants were used for all assays. Unless otherwise stated, experiments were carried out under tobacco chamber conditions. For the analysis of light effects, leaves were illuminated at 200 μmol quanta m^−2^ s^−1^ or dark-adapted for 3 h. Drought stress was visualized in detached leaves that were air-dried for 3 h. Controls were kept in distilled water for the same time. Salt stress was imposed by incubating leaf discs (12 mm in diameter) with either water, 0.25 M NaCl or 0.5 M NaCl for 16 h. For the biotic stress assay, detached leaves were punctured with 100 μl of a suspension of the necrotrophic bacterium *Pectobacterium carotovorum* corresponding to 10^8^ colony forming units (CFU)/ml in LB medium^85^, and incubated under tobacco chamber conditions for 16 h.

For image acquisition, leaf tissue of Arabidopsis or *N. tabacum*/*benthamiana* expressing NERNST targeted to the cytosol or chloroplasts were placed in a drop of distilled water on a microscope slide. Imaging of roots was performed in the same form. Leaf discs treated with DTT or H_2_O_2_ were vacuum-infiltrated for 20 min in order to obtain the fully reduced or oxidized sensor.

### Protoplast isolation and assay

Tobacco protoplasts were isolated from young leaves of 4-week-old plants expressing the NADP(H) biosensor directed to chloroplasts or cytosol as described by Müller et al.^86^. Plants were grown at 23 °C in magenta boxes containing 0.32% (w/v) Gamborg’s basal salt powder with 0.1% (v/v) B5 Mix Vitamins (bioWORLD, Dublin, OH), 4 mM MgSO_4_.7H_2_O, 43.8 mM sucrose and 0.8% (w/v) gelrite, pH 5.8 (SCN medium), supplemented with 30 µg/µl kanamycin (Duchefa Biochemie, Haarlem, Netherlands), under a 16-h photoperiod. Leaves were cut with scissors and the slices incubated overnight in the dark at 23 °C in F-Pin solution (10 mM MES pH 5.8, 0.32% Gamborg’s B5 basal salt powder with bioWORLD vitamins, 0.4 M sucrose) containing 0.5% (w/v) cellulase Onozuka R10 and macerozyme R10 (SERVA Electrophoresis GmbH, Heidelberg, Germany). After release of the protoplasts by soft shaking, the suspension was overlaid with MMM solution (5 mM MES pH 5.8, 15 mM MgCl_2_, 467 mM mannitol), and protoplasts counted in a Rosenthal chamber. They were washed with and resuspended in PCA medium (5 mM MES pH 5.8, 0.32% Gamborg’s B5 basal salt powder with vitamins, 2 mM MgSO_4_.7H_2_O, 3.4 mM CaCl_2_.2H_2_O, 0.342 mM l-glutamine, 58.4 mM sucrose, 444 mM glucose, 8.4 μM calcium pantothenate, 4 μg/ml biotin, 64.52 μg/μl ampicillin) at ~300,000 protoplasts per 1.8 ml, before immobilization in 0.75% (w/v) agarose (see below) to acquire confocal microscopy images. All solutions and media were filter-sterilized except for the SCN medium which was autoclaved.

Arabidopsis protoplasts were obtained from leaves of 4-week-old plantlets expressing the NADP(H) biosensor directed to chloroplasts or cytosol grown under Arabidopsis chamber conditions in Magenta boxes containing SCA medium (0.32% Gamborg’s basal salt powder with vitamins, 4 mM MgSO_4_.7H_2_O, 43.8 mM sucrose and 0.15% (w/v) phytoagar, pH 5.8) supplemented with 30 µg/µl kanamycin. Protoplasts were also isolated from leaves of 2- to 3-week-old Arabidopsis plants (ecotype Columbia), cultured in 12-cm square plates containing the same medium supplemented with 50 μg/μl ampicillin. For both types, a floatation method was employed for protoplast isolation^87^. Briefly, leaf material was sliced with a scalpel and incubated overnight in the dark at 23 °C in filter-sterilized MMC solution (10 mM MES pH 5.8, 40 mM CaCl_2_.2H_2_O, 467 mM mannitol) containing 0.5% (w/v) cellulase Onozuka R10 and macerozyme R10 (SERVA). After release of the protoplasts with a pipette, the suspension was transferred to a filter-sterilized MSC solution (10 mM MES pH 5.8, 0.4 M sucrose, 20 mM MgCl_2_.6H_2_O, 467 mM mannitol) and overlaid with MMM solution. Protoplasts were collected at the interphase and transferred to a filter-sterilized W5 solution (2 mM MES pH 5.8, 154 mM NaCl, 125 mM CaCl_2_.2H_2_O, 5 mM KCl, 5 mM glucose) prior to counting in a Rosenthal chamber.

The pCHF3 plasmids encoding NERNST (Supplementary Table 1) were introduced into protoplasts isolated from non-transformed Arabidopsis leaves by polyethylene glycol (PEG)-mediated transformation as described by Ochoa-Fernández et al.^88^. Briefly, ~500,000 protoplasts in 100 μl of MMM were mixed with 20 μg of plasmid, and the PEG solution (4 g of PEG-4000, 2.5 ml of 800 mM mannitol, 1 ml of 1 M CaCl_2_ and 3 ml H_2_O) was added dropwise. After 8 min of incubation, 120 μl of MMM and 1440 μl of PCA medium were added to the protoplast suspension to a final volume of 1.8 ml. Protoplasts were incubated in the dark for 20–24 h after transformation, concentrated and used immediately.

Protoplasts were immobilized in 0.75% (w/v) low-gelling agarose (Sigma Aldrich St. Louis, MO), carefully dispensed in agarose-containing PCA medium beads formed into 75 × 25-mm microscope slides warmed at 37 °C and covered with a 46 × 27-mm slide. Confocal microscopy images were acquired after cooling.

### Mammalian cell culture and transfection

HeLa (HELA; no.: ACC57, DSMZ, Braunschweig, Germany) and human embryonic kidney cells (HEK-293T; no.: ACC635, DSMZ) were seeded and cultivated overnight in Imaging Dish 1.5 plates (170-μm Cover Glass Bottom, Zell-kontakt GmbH) in Dulbecco’s modified Eagle’s medium (DMEM, PAN, cat. no. P03-0710) supplemented with 10% fetal bovine serum (DMEM_complete_) at 37 °C in a humidified atmosphere of 95% air and 5% CO_2_ (ref. ^89^.)

For HeLa and HEK-293T cell transfection, DNA sequences encoding Wt and mutant NERNST, NOX and roGFP2 (pPM014, pPM015, pPM017, pPM023 and pPM025 plasmids, respectively) were cloned between the SV40 promoter and terminator of the pMZ333 plasmid, as detailed in Supplementary Table 1. Primers used for amplification were oPM001 and oPM002 for Wt and mutant NERNST, oPM028 and oPM029 for NOX and oPM019, and oPM002 for roGFP2 (Supplementary Table 3). For mitochondrial targeting, the transit peptide of subunit 8 from the human cytochrome *c* oxidase complex was amplified from pCDNA3.1-MitoGFP, using oPM034/oPM035 as internal and oPM036/oPM037 as external primers, in a nested PCR reaction placed in one tube (Supplementary Table 3). The amplified fragment was cloned in-frame to the 5’-end of the NERNST coding sequence in plasmid pPM014, to yield pPM026 (Supplementary Table 1).

Suspensions containing 2 × 10^5^ cells were transfected using polyethyleneimine (PEI; Polysciences Inc. Europe, Hirschberg, Germany; no.23966-1) as described by Müller et al.^89^. Briefly, aliquots containing 3 µg of DNA were diluted in 200 µl of OptiMEM (Invitrogen) and mixed with 10 µl of PEI solution in 200 µl of OptiMEM under vortexing. After 15 min of incubation at 25 °C, the PEI-OptiMEM-DNA precipitate was added to the cells. The culture medium was replaced 4 h after the transfection. All plasmids were transfected in equal amounts (w:w). Cells were imaged at 24 h post-transfection in a confocal microscope.

For experiments carried out with the PerkinElmer Operetta CLS confocal high-content imaging system, 1 × 10^6^ HeLa cells were seeded and transfected in Petri Dishes (Corning 430167, Corning, Inc) in DMEM_complete_. Four h after transfection, the culture was trypsinised and 1.5 × 10^4^ cells in fresh medium were transferred to optical quality 96-well plates (PerkinElmer Cell Carrier Ultra 96-well) and imaged the following day.

### G6PDi-1 and DPI assays

Experiments involving inhibition of glucose 6-phosphate dehydrogenase (G6PDH) were performed on the PerkinElmer Operetta CLS. Cells were suspended in Live Cell Imaging Solution (LCIS, Thermo Fisher, Waltham, MA) containing 20 mM HEPES pH 7.4, 140 mM NaCl, 2.5 mM KCl, 1.8 mM CaCl_2_, 1 mM MgCl_2_, supplemented with high glucose (4.5 g/l). Two hours later, a 2× solution containing the G6PDH inhibitor (G6PDi-1^71^, Biomol GmbH) or dimethyl sulfoxide (DMSO) (for control) was added to each well and measured every 5 min for 4 h. The entire experiment was carried out on a single plate.

For diphenyleneiodonium chloride (DPI, Sigma Aldrich St. Louis, MO) experiments, the reagent was added to a final concentration of 50 µM and its effects measured after 5 min of incubation in a confocal microscope.

### In vitro mRNA transcription, animal handling and microinjection of zebrafish embryos

DNA sequences encoding NERNST and HyPerRed, a fluorescent H_2_O_2_ sensor^76^, were PCR-amplified from pPM003 and pC1-HyPerRed (Addgene plasmid # 48249; http://n2t.net/addgene:48249; RRID: Addgene_48249; Supplementary Table 1) using primers oAK003/oAK004 and oAK005/oAK006, respectively (Supplementary Table 3). The amplified products were cloned in the pCS2+MT vector system between the SP6 promoter and SV40 terminator to yield plasmids pAK001 and pAK002 (Supplementary Table 1). They were purified with the Wizard DNA purification system (Promega, Madison, WI) and linearized by digestion with NotI. Forty ng of linearized plasmid were in vitro transcribed with the mMESSAGE mMACHINE® SP6 Transcription Kit from Ambion (Thermo Fisher) following the manufacturer’s instructions.

Adult zebrafish were maintained at 28 °C on a 14:10 h light:dark cycle as previously described^90^. Zebrafish matings were carried out by crossing three males and four females in the same spawning tank. All embryos were staged according to morphological development in hours post-fertilization (hpf) at 28 °C (ref. ^91^) and handled in compliance with relevant international guidelines (Policy on Humane Care and Use of Laboratory Animals and American Veterinary Medical Association, https://zfin.org/). Embryos were injected at the 1-cell stage into the yolk immediately below the cell using a gas-driven microinjection apparatus (MPPI-2 Pressure Injector, Applied scientific Instrumentation; Eugene, OR, USA). Five nl of 100 ng/μl dilutions of the NADP(H) and H_2_O_2_ sensor mRNAs were co-microinjected. Embryos were allowed to develop until 48 hpf at 28 °C, manually dechorionated, and treated with tricaine^92^. Larvae were placed on a 1.2% (w/v) agarose chamber and tail fin tips were cut with a sharper under a scope. The injured larvae were mounted on a drop of 3% (w/v) methyl cellulose for visualization by confocal microscopy. The tail fin tip experiment and quantification were independently repeated three times with at least four specimens each.

### Confocal laser microscopy and image analysis

Images of bacteria, whole plant and zebrafish were taken with a Zeiss 8000 laser scanning confocal microscope (Zeiss ZEN BLACK 2.3 software), whereas images of protoplasts and mammalian cells were obtained with a Nikon Eclipse Ti with C2 Plus confocal laser scanning microscope (NIS-Elements imaging 5.01. software). In both cases, samples were excited at 405/488 nm and the emission was collected at 510 nm^52^. Chlorophyll auto-fluorescence was detected following excitation at 488 nm and emission at 650 nm. For co-imaging of NADP(H) and H_2_O_2_ dynamics, the intensiometric HyperRed sensor was excited at 543 nm and its fluorescence emission measured at 610 nm. The final processing of images was carried out with the ImageJ software.

Excitation filters at 355–385 nm and 460–490 nm were used for images obtained with the PerkinElmer Operetta CLS confocal high-content imaging system (Harmony 4.8 software). The emission filter was selected for light between 500 and 550 nm. To label mitochondria, cells were incubated with the MitoTracker Red CMXRos probe (Invitrogen) and followed by excitation using a filter at 530–560 nm, with the corresponding emission recording at 570-650 nm. Images were analyzed with the CellProfiler software^93^ and KNIME.

Dynamic ranges were determined by image analysis after exposing the different systems to reductants (DTT) and oxidants (H_2_O_2_ or diamide) as follows: a) bacteria, 10 min with 10 mM DTT or 10 mM H_2_O_2_; b) mammalian cells, 2 min with 10 mM DTT, 10 mM H_2_O_2_, 10 mM diamide; c) leaves, 20 min in a vacuum chamber with 50 mM DTT or 50 mM H_2_O_2_; d) leaf protoplasts, 5–10 min with 10 mM DTT or 10 mM H_2_O_2_.

### Fluorescence data analysis

Images of the biosensor were analyzed using the ImageJ software, unless otherwise stated. Raw data were exported to ImageJ as 32-bit TIFF files and the complete field or a user’s defined Region of Interest^52^ (ROI), when indicated, were taken for analysis. Determinations of R values were corrected for background emission intensity by subtracting the intensity of an adjacent cell-free ROI and using the Otsu algorithm threshold to generate masks of positive expression. The corrected 405-nm fluorescence intensity image was divided by the corrected 488-nm fluorescence intensity image to produce a ratio image on a pixel by pixel basis through the command “Image Expression Parser”. The grayscales of the ratio images were colored using the ImageJ look-up table ‘Fire’. Image analysis was automated through scripts we developed using ImageJ macros. Data obtained from ImageJ were imported, concatenated and prepared for analysis in RStudio (RStudio: Integrated Development for R, http://www.rstudio.com/). Images of zebrafish were taken from stacks acquired with an 18-µm *Z*-axis scaling. The maximum intensity projection of each stack was used for ratio processing, as previously described. Longitudinal studies in mammalian cells were carried out by using an ROI in the images obtained over time. This allowed “Cell-by-Cell” tracking.

For experiments carried out in the PerkinElmer Operetta CLS confocal high-content imaging system, image analyses were performed with custom scripts and the CellProfiler^93^ image analysis software. Numeric data were further processed using the KNIME data analysis platform and the R programming language. Analysis pipelines for both software packages and R scripts are available in the GitHub repository found at https://github.com/PameeMolinari/NCOMMS-22-05375-T^94^.

### NADP(H) quantification by redox cycling

The protocol of Slater et al.^55^ was modified to determine NADP(H) levels in bacterial extracts. Briefly, *E. coli* BL21 DE3 cells transformed with pM002/3 and pGroE7 (Supplementary Table 1) were grown for 16 h at 37 °C in LB or the corresponding minimal media and collected by centrifugation. For the determination of total NADP(H) contents, pellets were suspended in 400 µl of 0.2 M NaOH, immediately neutralized with 100 µl of 1 M Tris-HCl pH 8.0 and 200 µl of 0.2 N HCl, and centrifuged for 5 min at 16,000 *g*. Then, 200 µl of the supernatants were adjusted to pH 8 with a microelectrode. For NADPH measurements, cellular pellets were suspended in 400 µl of 0.2 M NaOH, and centrifuged. The NADP^+^ present in a 200-µl aliquot of the supernatants was destroyed by heating at 95 °C for 90 s, the solution cooled immediately in ice and neutralized as described before. The reaction was carried out in 1 ml of 25 mM Tris-HCl pH 8, 3.6 mM G6P, 100 mM 2,6-dichlorophenolindophenol, 0.5 mM phenazine methosulphate, 1 unit/ml G6PDH and 5-50 µl sample, following the change of absorbance at 600 nm in a Shimadzu spectrophotometer (UVProbe ver. 3.1. software). Concentrations were calculated from a standard curve (0–400 pmol NADPH). NADP^+^ contents were calculated by difference.

A similar protocol was employed to determine NADP(H) levels in whole-leaf extracts. Briefly, 100 mg of tobacco or Arabidopsis leaves were collected in eppendorf tubes and immediately frozen in liquid N_2_. The youngest fully expanded leaves from 7-week-old tobacco or 4-week-old Arabidopsis plants were used. For the determination of NADP^+^ or NADPH contents, the tissue was ground and suspended in 0.2 N HCl or 0.2 N NaOH, respectively. After centrifugation, an aliquot was separated and the NADPH or NADP^+^ present in the respective sample was selectively destroyed by heating at 100 °C for 60 s. The extracts were immediately neutralized and the reaction was carried out as described before. Concentrations were calculated from a standard curve.

### Analytical procedures

NERNST purification was followed by SDS-PAGE and immunoblotting with anti-GFP antisera, dilution 1:500 (Santa Cruz Biotechnology), using standard procedures^95^. Gel loading was carried out on the basis of protein mass, as determined by the Sedmak and Grossberg^96^ procedure.

### Statistics and reproducibility

Sample sizes were chosen according to the state of the art for experimental setups comprising genetically encoded biosensors in bacteria, animal and plant cells, and these numbers are according to our experience adequate for the kind of experiments and measurements performed. No outliers were excluded from the analysis. Randomization is not relevant to our study as the operator cannot influence the outcome of the measurement. The investigators were not blinded to allocation during experiments and outcome assessment. In general, different conditions, times, transformation batches, etc. are and must be known by the operator and cannot be blinded. In any case, the operator cannot influence the outcome of the determination. Data are presented as mean values  ±  SD (standard deviation) or ± SEM (standard error of the mean). Data analysis, calculation of corresponding *P* values, and generation of graphs were carried out using GraphPad Prism 9 (GraphPad Software, Inc.) and RStudio. Significant differences refer to statistical significance at various *P* values. *P* value < 0.05 was considered the threshold for statistical significance. *P* value significance intervals (*) and *n* values are provided within each figure legend and in the Source Data files, together with the statistical test performed for each experiment. Source data, including all individual *n* values and exact *P* values, are provided as a Source Data file. Representative images and data shown were produced in at least two independent experiments.

### Reporting summary

Further information on research design is available in the Nature Portfolio Reporting Summary linked to this article.

## Supplementary information

Supplementary Information File

Reporting Summary

## Data Availability

All data associated with this study are available within the article and its Supplementary Information files. Source data are provided with this paper.

## Source data

Source Data

## Code availability

Analysis pipelines for both software packages and Macro and R scripts used for analyzing microscopy images in ImageJ or CellProfiler are available in a GitHub repository, https://github.com/PameeMolinari/NCOMMS-22-05375-T^94^.
