# Supplementary material for: NERNST: a genetically-encoded ratiometric non-destructive sensing tool to estimate NADP(H) redox status in bacterial, plant and animal systems
Source: Nat Commun. 2023 Jun 6;14:3277. doi: 10.1038/s41467-023-38739-4 (PMC10244373; doi:10.1038/s41467-023-38739-4)

NERNST: a genetically-encoded ratiometric non-destructive sensing tool to estimate NADP(H) redox status in bacterial, plant and animal systems

Pamela E. Molinari[1], Adriana R. Krapp[1], Andrea Weiner[1], Hannes M. Beyer[2], Arun Kumar Kondadi[3], Tim Blomeier[2], Melina López[1], Pilar Bustos-Sanmamed[1], Evelyn Tevere[1], Wilfried Weber[4,5], Andreas S. Reichert[3], Nora B. Calcaterra[1], Mathias Beller[6], Nestor Carrillo[1,*] and Matias D. Zurbriggen[2,7,]

[1]Instituto de Biología Molecular y Celular de Rosario (IBR-UNR/CONICET), Facultad de Ciencias Bioquímicas y Farmacéuticas, Universidad Nacional de Rosario (UNR), 2000 Rosario, Argentina

[2]Institute of Synthetic Biology, University of Düsseldorf, Düsseldorf, Germany

[3]Institute of Biochemistry and Molecular Biology I, Medical Faculty and University Hospital Düsseldorf, Heinrich-Heine-University Düsseldorf, Düsseldorf, Germany

[4]Faculty of Biology and Signalling Research Centres BIOSS and CIBSS, University of Freiburg, Freiburg, Germany

[5]Present address: INM - Leibniz Institute for New Materials and Department of Materials Sciences and Engineering, Saarland University, Saarbrücken, Germany

[6]Institute of Mathematical Modeling of Biological Systems, University of Düsseldorf, Düsseldorf, Germany

[7]CEPLAS – Cluster of Excellence on Plant Sciences, Düsseldorf, Germany

*E-mail to: carrillo@ibr-conicet.gov.ar and matias.zurbriggen@uni-duesseldorf.de

Supplementary Figures 1-21

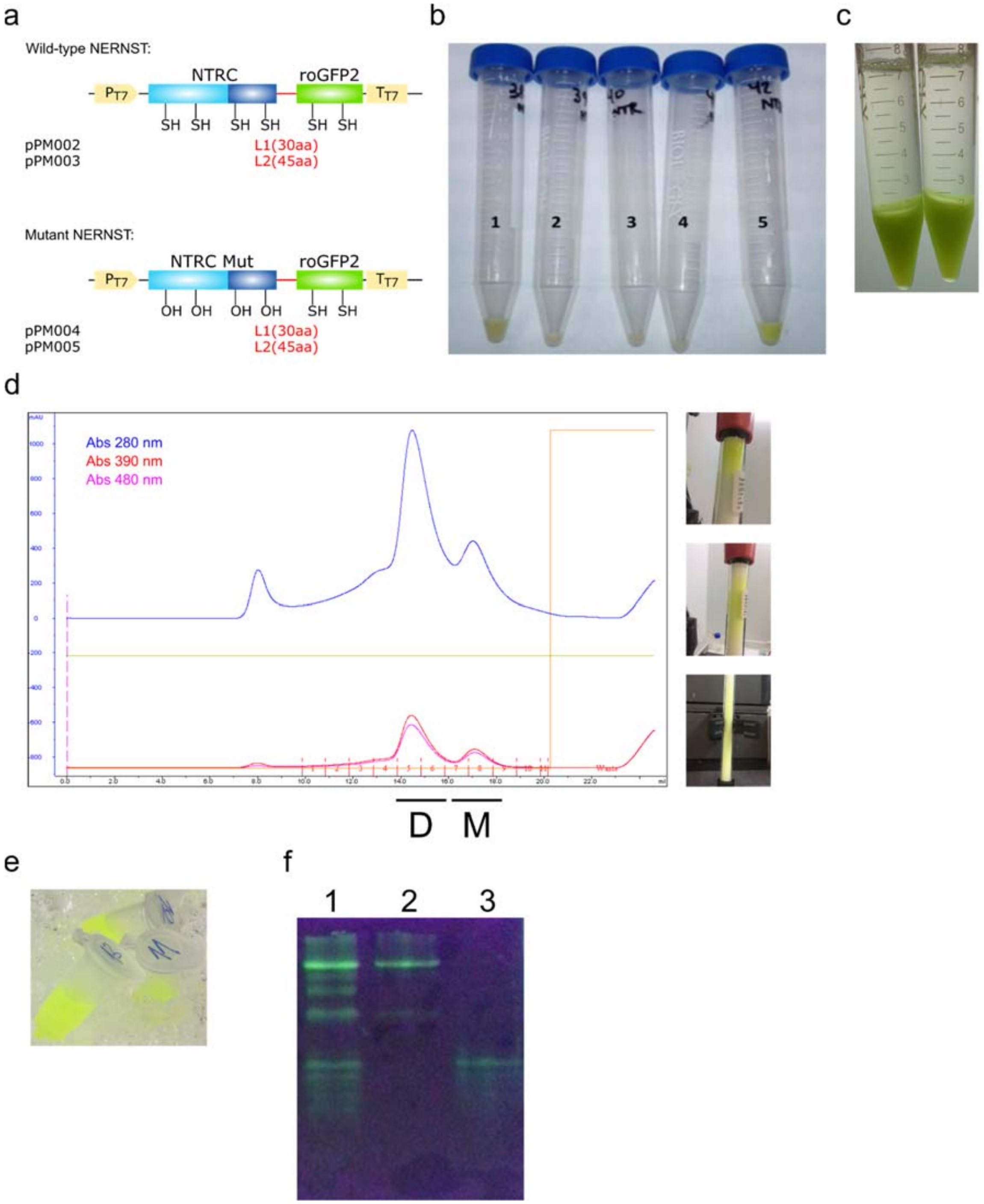

Supplementary Figure 1. Preparation and purification of NERNST. (a) Construction of pET_TEV vectors for expression of NERNST in *E. coli*. Light blue and blue bars indicate regions encoding the NTR and Trx domains of NTRC, respectively. The spacer arm of variable length connecting NTRC with roGFP2 (green) is depicted as a red line. The T7 promoter (P_{T7}) and terminator (T_{T7}) regions are shown as yellow

boxes. The lower vector represents the construct encoding Cys-to-Ser mutants (Mut). Sulfhydryl (-SH) and hydroxyl (-OH) groups are superimposed to the coding sequence of NERNST to illustrate these substitutions. L1: 30-aa linker. L2: 45-aa linker. **(b)** Bacterial pellets obtained after expression of the biosensor in BL21(DE3) cells co-transformed with plasmids TF16 (1), pKJE7 (2), pGTF2 (3), pGKJE8 (4) and pGro7 (5) from Takara (Takara Bio Inc). Cells were grown overnight in 10 ml of LB broth (see Online Methods). **(c)** Ni-sepharose beads after incubation with cleared extracts of pGRO7 cells expressing NERNST. **(d)** Superdex-200 Increase 10/300 GL chromatogram monitored at the indicated wavelengths. D, dimer; M, monomer. **(e)** Fractions of purified NERNST dimer after size exclusion chromatography. **(f)** Native PAGE visualized under UV light. Lane 1, total extract (control); 2, Dimer fraction; 3, Monomer fraction. The Native gel was performed once.

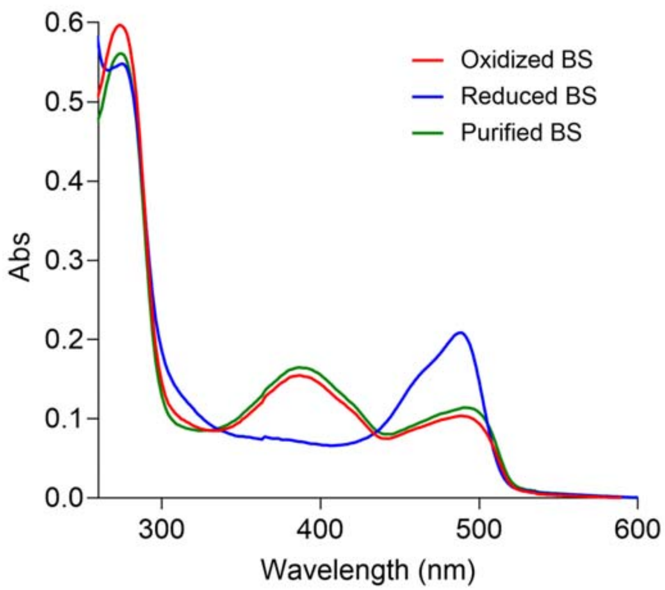

Supplementary Figure 2. Absorption spectra of oxidized and reduced NERNST. Purified biosensor (green) was incubated with 10 mM H_2O_2 (red) or 10 mM DTT (blue) in 100 mM K_3PO_4 pH 7.3, 150 mM NaCl, for 5 min prior to spectra recording. Spectra of oxidized and reduced biosensor (BS) were not corrected for the 10% dilution resulting from addition of H_2O_2 or DTT.

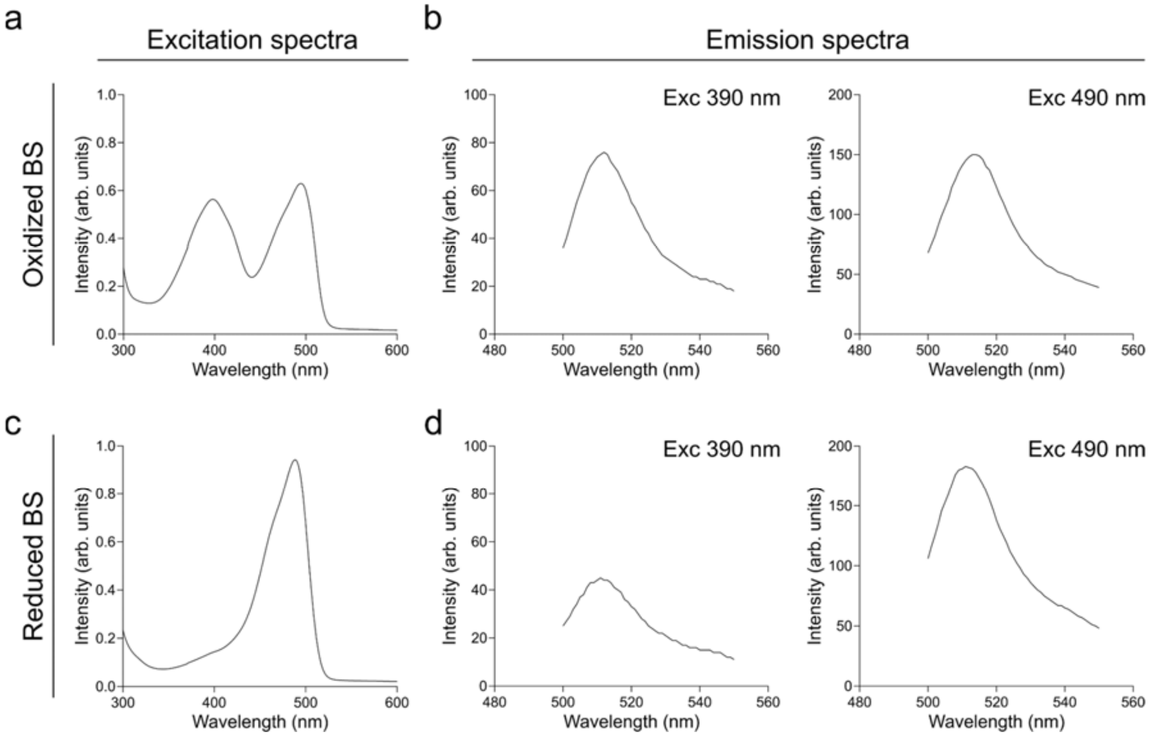

Supplementary Figure 3. Excitation and emission spectra of purified NERNST. (a) Excitation spectrum of oxidized biosensor (BS). **(b)** Emission spectra of oxidized biosensor after excitation at 390 nm and 490 nm. **(c)** Excitation spectrum of DTT-reduced NERNST. **(d)** Emission spectra of reduced NERNST after excitation at 390 nm and 490 nm. Spectra were recorded in 100 mM K_3PO_4 pH 7.3, 1 mM EDTA, 150 mM NaCl. Arb. Units, arbitrary units; Exc, excitation.

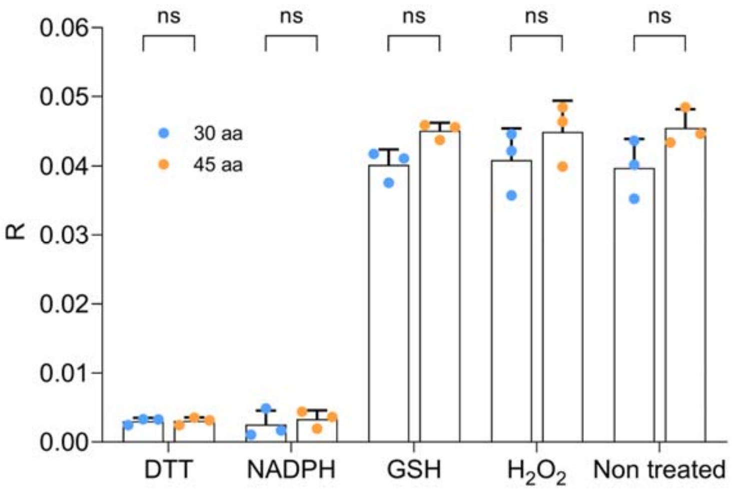

Supplementary Figure 4. NERNST dimers containing 30-aa and 45-aa spacer arms are equally reduced by NADPH. The redox state of the biosensor was estimated by the corresponding R values after incubation with 10 mM DTT, 10 mM H_2O_2, 0.5 mM NADPH or 0.5 mM GSH in 100 mM K_3PO_4 pH 7, 150 mM NaCl for 5 min. Fluorescence changes were determined in a Synergy 2 Biotek fluorimetric plate reader (Himex, Turku) and R values were calculated as indicated in the text. Data shown are means ± SD of 3 independent determinations. Two-way ANOVA followed by Bonferroni's multiple comparisons test. ns, non-significant.

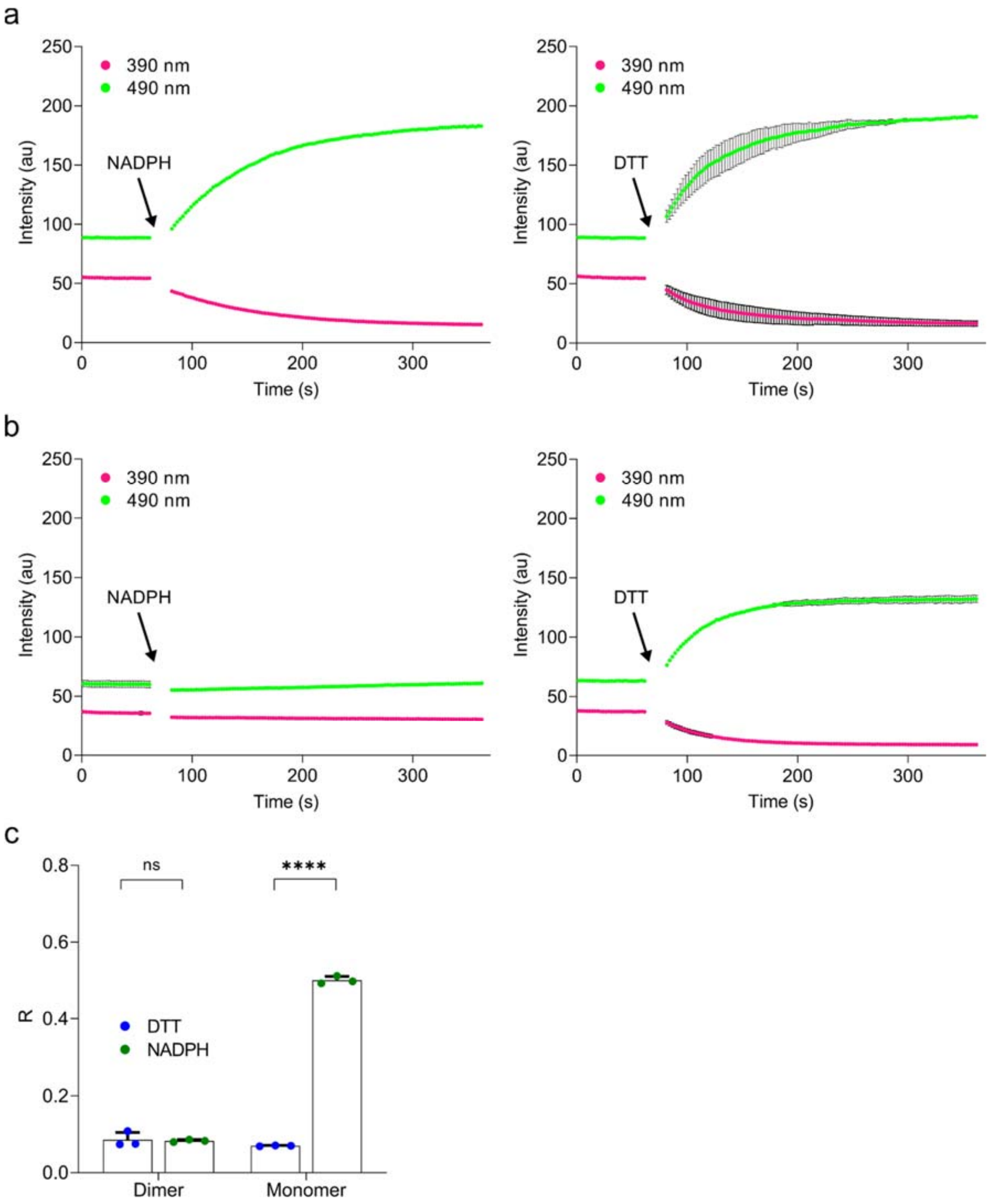

Supplementary Figure 5. Monomeric NERNST is not reduced by NADPH. Time course of fluorescence changes at 510 nm upon excitation at 390 nm (magenta) and 490 nm (green) upon incubation of NERNST dimer **(a)** or monomer **(b)** with 0.5 mM NADPH or 10 mM DTT in 100 mM K_3PO_4 pH 7, 150 mM NaCl. Fluorescence was recorded in a Cary Eclipse fluorescence spectrophotometer. R values **(c)** were

obtained through division of the fluorescence readout at 390 nm by that at 490 nm at 6 min. Data shown in **(a, b)** are means ± SEM of 3 independent determinations. **(c)** Data shown are means ± SD of 3 different determinations. Two-way ANOVA followed by Bonferroni's multiple comparisons test. ****$P \leq 0.0001$; ns, non-significant. Source data, including exact *P* values, are provided as a Source Data file.

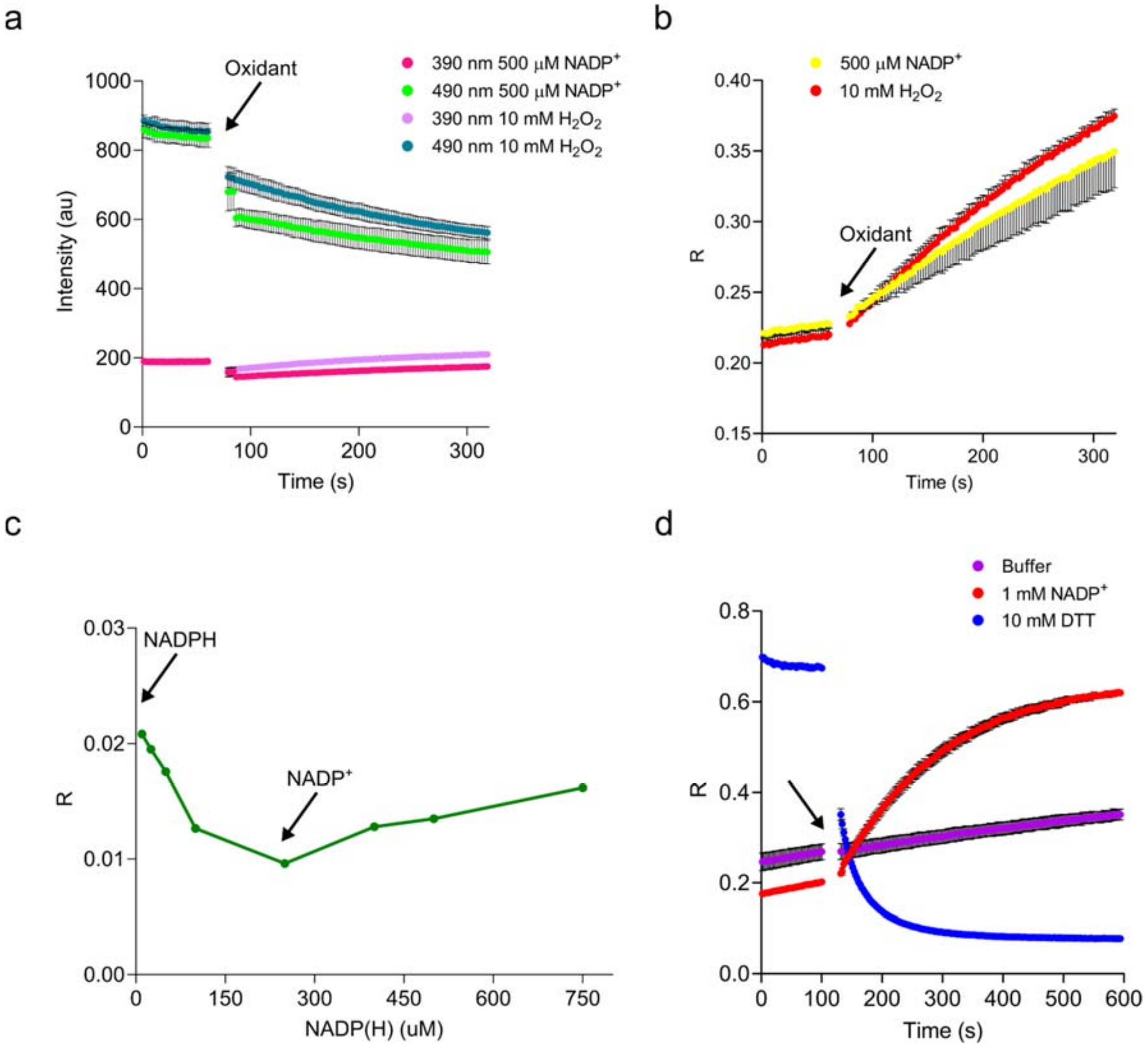

Supplementary Figure 6. NADP(H)-dependent redox changes of NERNST are fully reversible. (a, b) The biosensor was reduced with 10 mM DTT for 6 min, and after removal of the reductant by gel filtration, incubated with 0.5 mM $NADP^+$ or 10 mM H_2O_2 under the conditions indicated in the legend to Supplementary Fig. 5. Calculation of R values **(b)** is also described there. **(c)** NERNST responds to $NADP^+$ in the presence of NADPH. The biosensor was incubated with 0.25 mM G6P, 1 unit of G6PDH and the indicated concentrations of $NADP^+$ in 100 mM K_3PO_4 pH 7, 150 mM NaCl. $NADP^+$ will be converted into NADPH by G6PDH until exhaustion of G6P (0.25 mM). Subsequent additions of $NADP^+$ lead to NERNST oxidation in the presence of 0.25 mM NADPH. Fluorescence was recorded at 6 min. **(d)** Oxidation of DTT-reduced NERNST by $NADP^+$. About 90% of the fluorescence decrease undergone by the biosensor upon reduction was recovered by reaction with $NADP^+$. The slow oxidation

shown in the “Buffer” curve was attributed to reaction with dissolved oxygen. Other conditions are those of Supplementary Fig. 5. Data shown in **(a, b, d)** are means ± SEM of 3 independent determinations. **(c)** Data shown are means ± SD of 2 different determinations.

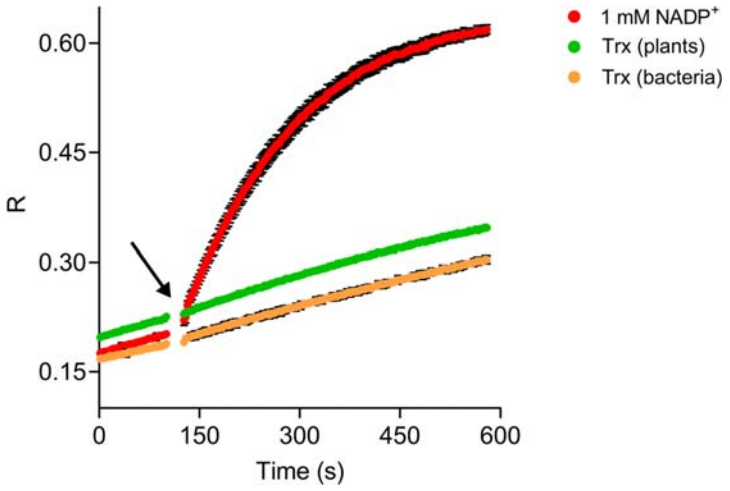

Supplementary Figure 7. DTT-reduced NERNST is not oxidized by bacterial or plant thioredoxins. *E. coli* thioredoxin (Trx) A and peach Trx *h* were used at 10 µM, $NADP^+$ was 1 mM. All other conditions are those of Supplementary Figure 5. Data shown in are means ± SEM of 3 independent determinations.

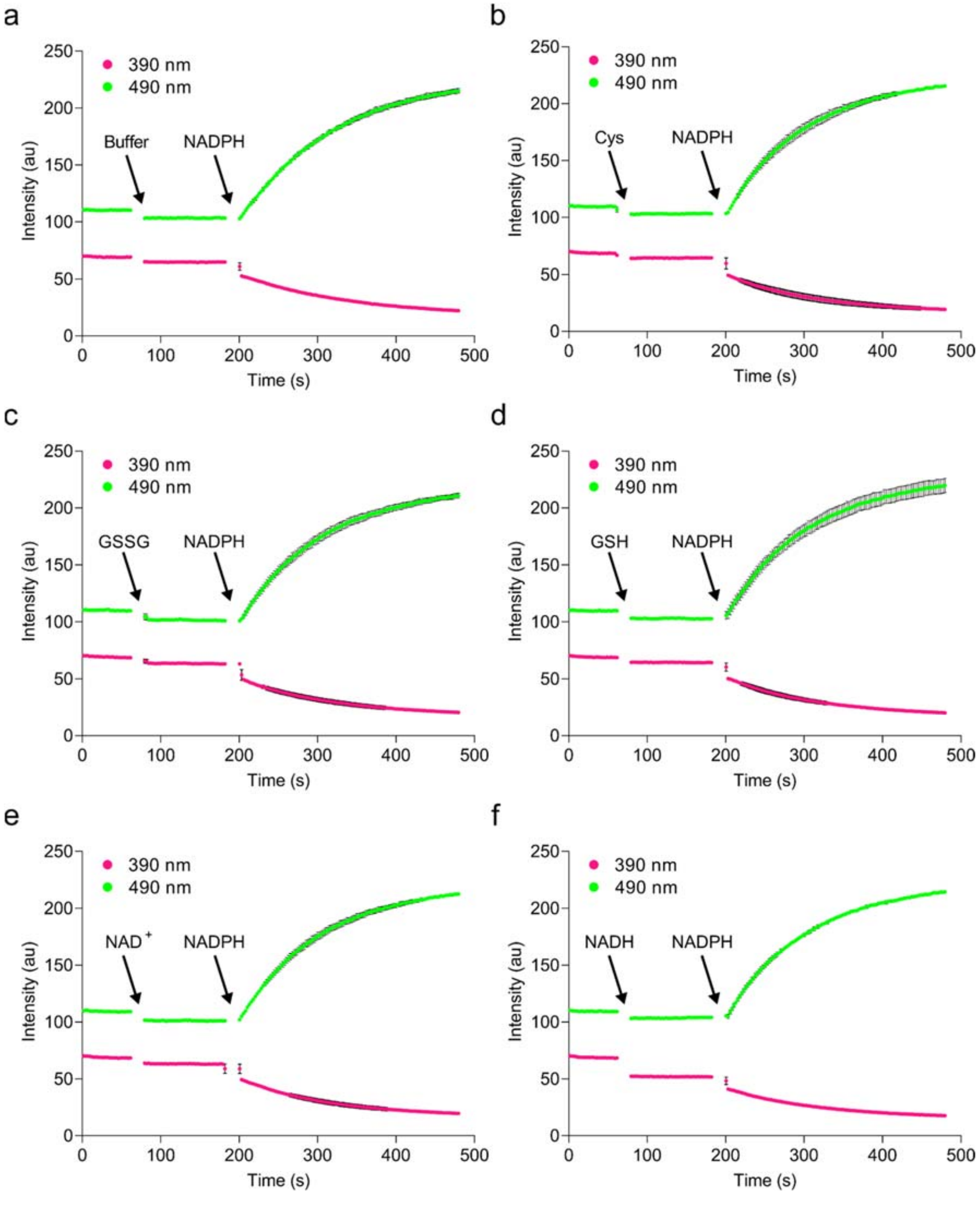

Supplementary Figure 8. NERNST reduction by NADPH is not affected by the presence of other redox-active metabolites. The biosensor was incubated with 0.5 mM NADPH after a 1-min incubation with **(a)** buffer, or 0.5 mM each of **(b)** Cys, **(c)** GSSG, **(d)** GSH, **(e)** NAD^+ or **(f)** NADH. Other conditions are those of Supplementary Fig. 5. Data shown in **(a-f)** are means ± SEM of 3 independent determinations.

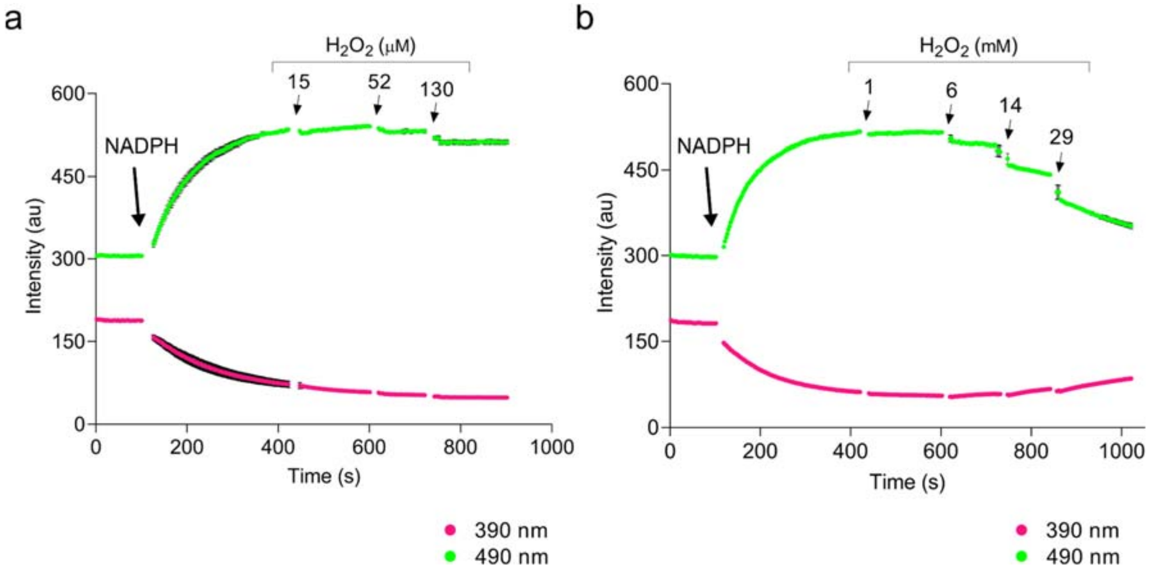

Supplementary Figure 9. NERNST oxidation by H_2O_2 in the presence of NADPH requires high concentrations. The biosensor was reduced by 0.5 mM NADPH and subsequently exposed to H_2O_2 in the low micromolar **(a)** or millimolar **(b)** range. Other conditions are those of Supplementary Fig. 5. Data shown in **(a, b)** are means ± SEM of 3 independent determinations.

a

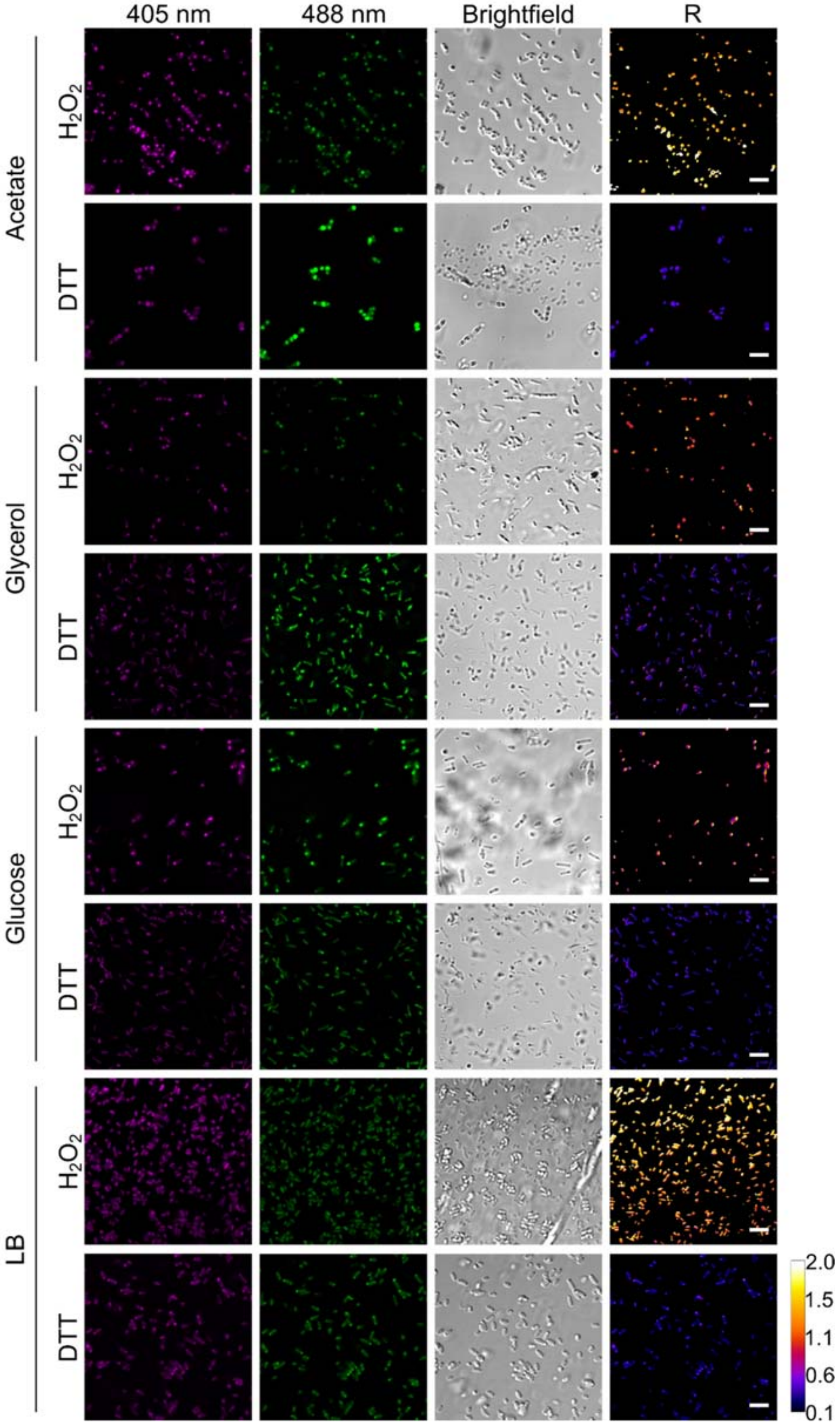

405 nm
488 nm
Brightfield
R
Acetate
H_2O_2
DTT
Glycerol
H_2O_2
DTT
Glucose
H_2O_2
DTT
LB
H_2O_2
DTT
2.0
1.5
1.1
0.6
0.1

187

188

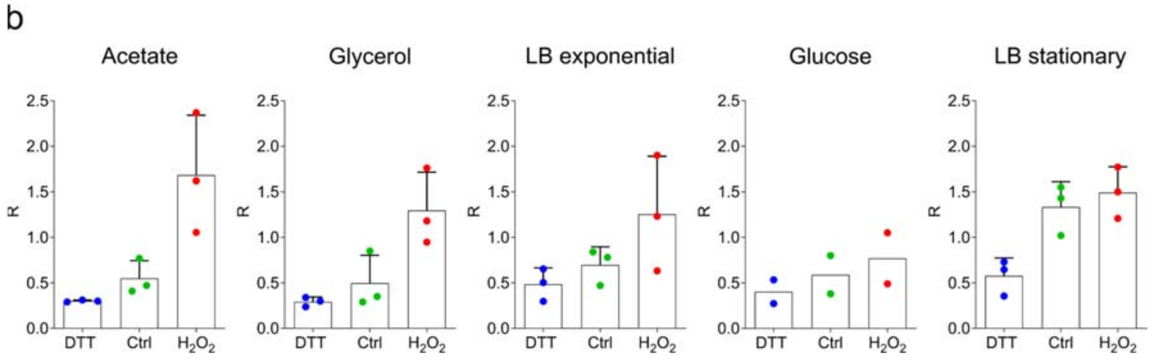

Supplementary Figure 10. Dynamic ranges of NERNST expressed in *E. coli* cells. **(a)** Cells grown in the indicated media to mid-exponential phase were exposed to 10 mM H_2O_2 or 10 mM DTT and subsequently analyzed by confocal microscopy. Panels show, from left to right, representative images of the fluorescence emission at 510 nm after excitation at 405 nm and 488 nm, the brightfield and the resulting R values. Scale bars, 5 µm. Pseudocolor scale = R values. **(b)** Bar graphs with individual data points depict the ratiometric behavior of fully reduced, control or resting state, and fully oxidized cells in each medium. LB, Luria-Bertani broth. Data are shown as means ± SD of 2 or 3 independent determinations.

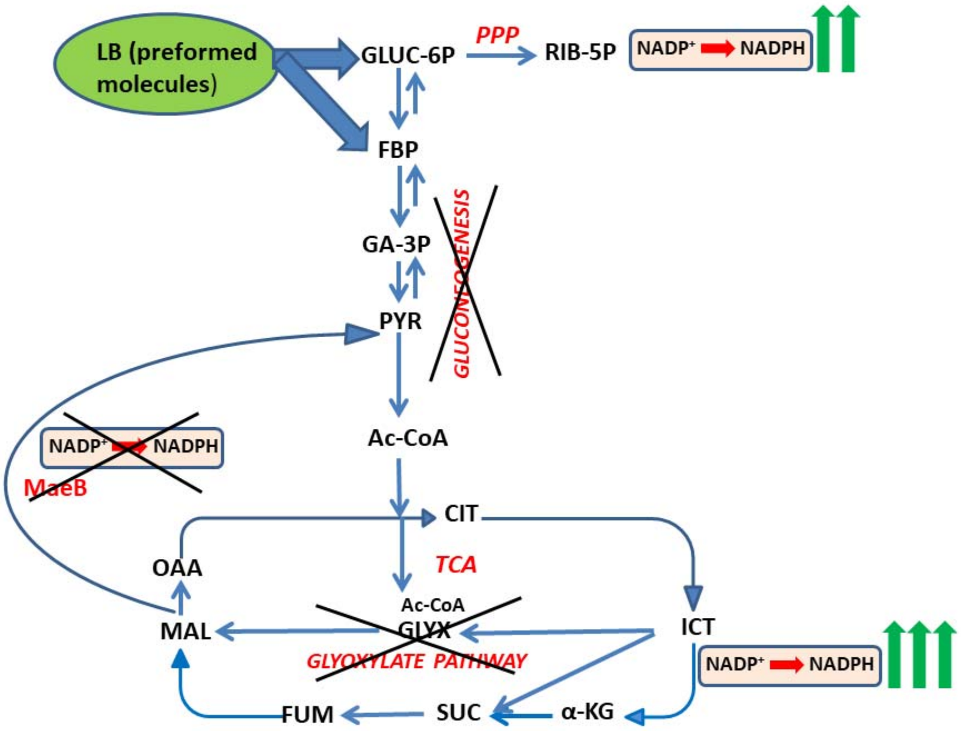

Supplementary Figure 11. Major metabolic pathways operating during *E. coli* growth in LB rich medium. Carbon metabolism proceeds through three main pathways under these culture conditions: glycolysis, the pentose phosphate pathway (PPP) and the tricarboxylic acid cycle (TCA). The presence of preformed molecules in rich media results in rapid initial growth of the bacteria, associated to the abundance of enzymes belonging to these metabolic pathways, and leading to increased carbon flux toward the TCA. Other routes of carbon metabolism such as gluconeogenesis, the glyoxylate pathway and the $NADP^+$-dependent malic enzyme MaeB are instead down-regulated[1]. During the exponential phase of growth, most NADPH is produced by the PPP and by the activity of isocitrate dehydrogenase at the TCA. The number of arrows provides a visual indication of the amount of NADPH produced at each step. As cells enter the stationary phase, NADPH levels are expected to decay mainly due to the depletion of substrates. Abbreviations used: Ac-CoA, acetyl-coenzyme A; CIT, citrate; FBP, fructose-1,6-bisphosphate; FUM, fumarate; Gluc-6P, glucose-6P; GLYX, glyoxylate; GA-3P, glyceraldehyde-3P; ICT, isocitrate; α-KG, α-ketoglutarate; MAL, malate; OAA, oxaloacetate; PYR, pyruvate; Rib-5P, ribulose-5P; SUC, succinate. Based on Li et al.[1]

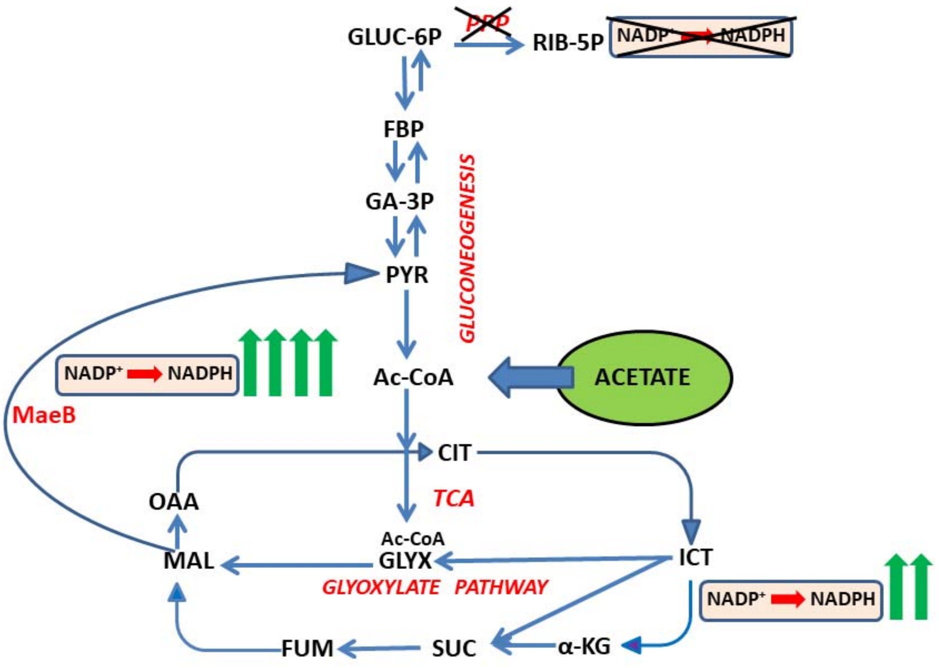

Supplementary Figure 12. Major metabolic pathways operating during *E. coli* growth in acetate minimal medium. In this medium, acetate is converted to acetyl-CoA (Ac-CoA) by Ac-CoA synthetase, which is further metabolized at the tricarboxylic acid cycle (TCA) or used for the synthesis of malate (MAL) through the glyoxylate shunt. Enzymes of the gluconeogenic pathways and TCA are induced in acetate minimal medium, together with the malic enzyme MaeB, whereas genes encoding the first two enzymes of the PPP are significantly down-regulated[2]. Moreover, a large fraction (75%) of isocitrate dehydrogenase (IDH) is inactivated by reversible phosphorylation catalyzed by IDH kinase/phosphatase, whose function is central to successful adaptation and growth of *E. coli* on acetate[3]. Arrows indicate the major points of NADPH production under these growth conditions. Other abbreviations used are listed in the legend to Supplementary Figure S11. The model is based upon Oh et al.[2]

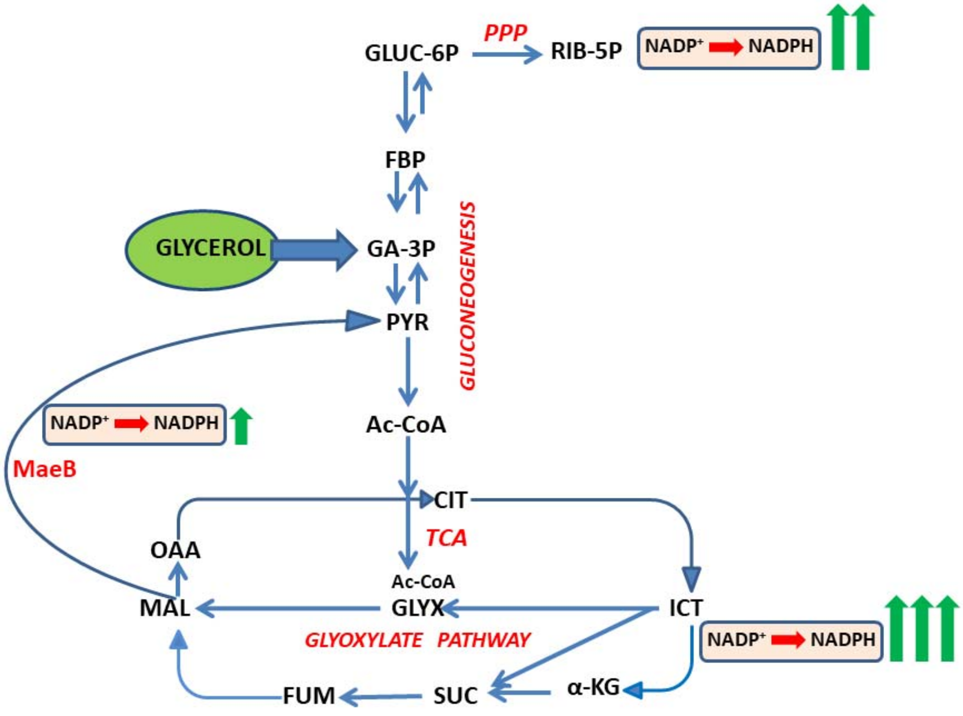

Supplementary Figure 13. Major metabolic pathways operating during *E. coli* growth in glycerol minimal medium. In this medium, glycerol is converted into glyceraldehyde 3-phosphate (GA-3P) which is a key metabolite that can be utilized in several pathways including glycolysis and the PPP[4]. Gluconeogenic carbon recycling is occurring simultaneously to glycolysis. Moreover, the glyoxylate shunt and TCA genes are also up-regulated when bacterial growth relies on glycerol as the sole carbon source[2,5], whereas the activity of MaeB is comparatively low in glycerol[4]. Accordingly, main NADPH sources are the PPP and the TCA (arrows). Other abbreviations used are listed in the legend to Supplementary Figure S11. The model is based upon Martínez-Gómez et al. New insights into Escherichia coli metabolism: carbon scavenging, acetate metabolism and carbon recycling responses[4].

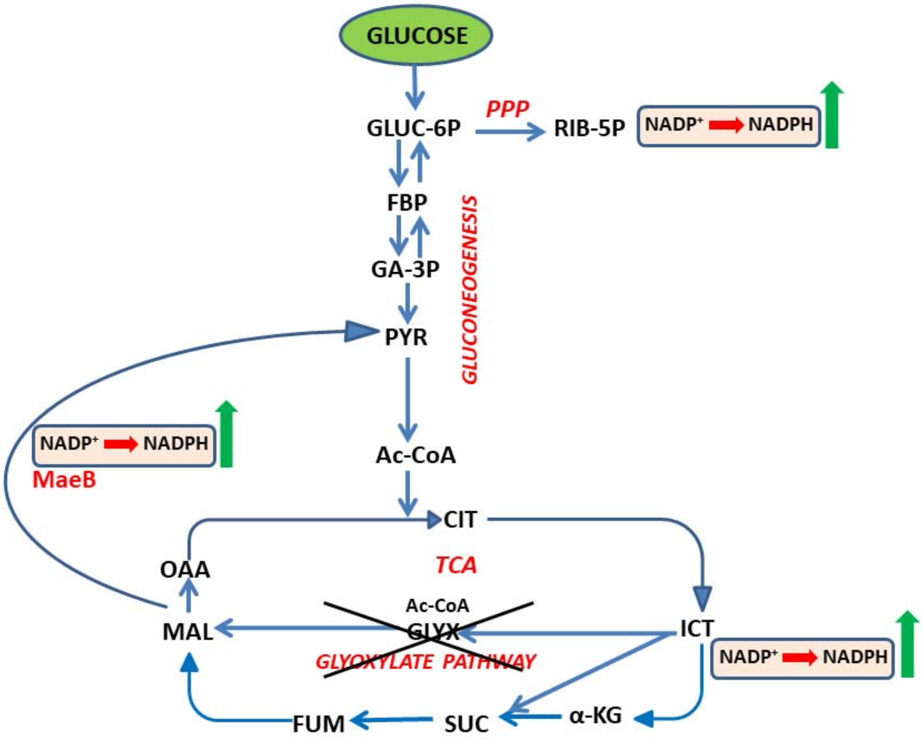

Supplementary Figure 14. Major metabolic pathways operating during *E. coli* growth in glucose minimal medium. In this medium, enzymes involved in the upper glycolytic pathway and anaplerotic reactions accumulate to slightly higher amounts compared to rich media. As shown by the arrows, MaeB, the PPP and isocitrate dehydrogenase at the TCA are the major producers of NADPH in glucose minimal medium[1,6]. The glyoxylate pathway is instead down-regulated and isocitrate lyase, one of the rate-limiting enzymes of the pathway, becomes undetectable[4]. As a consequence, growth is slow and NADPH levels are lower than in rich media. Other abbreviations used are listed in the legend to Supplementary Figure S11. The model is based upon Li et al.[1]

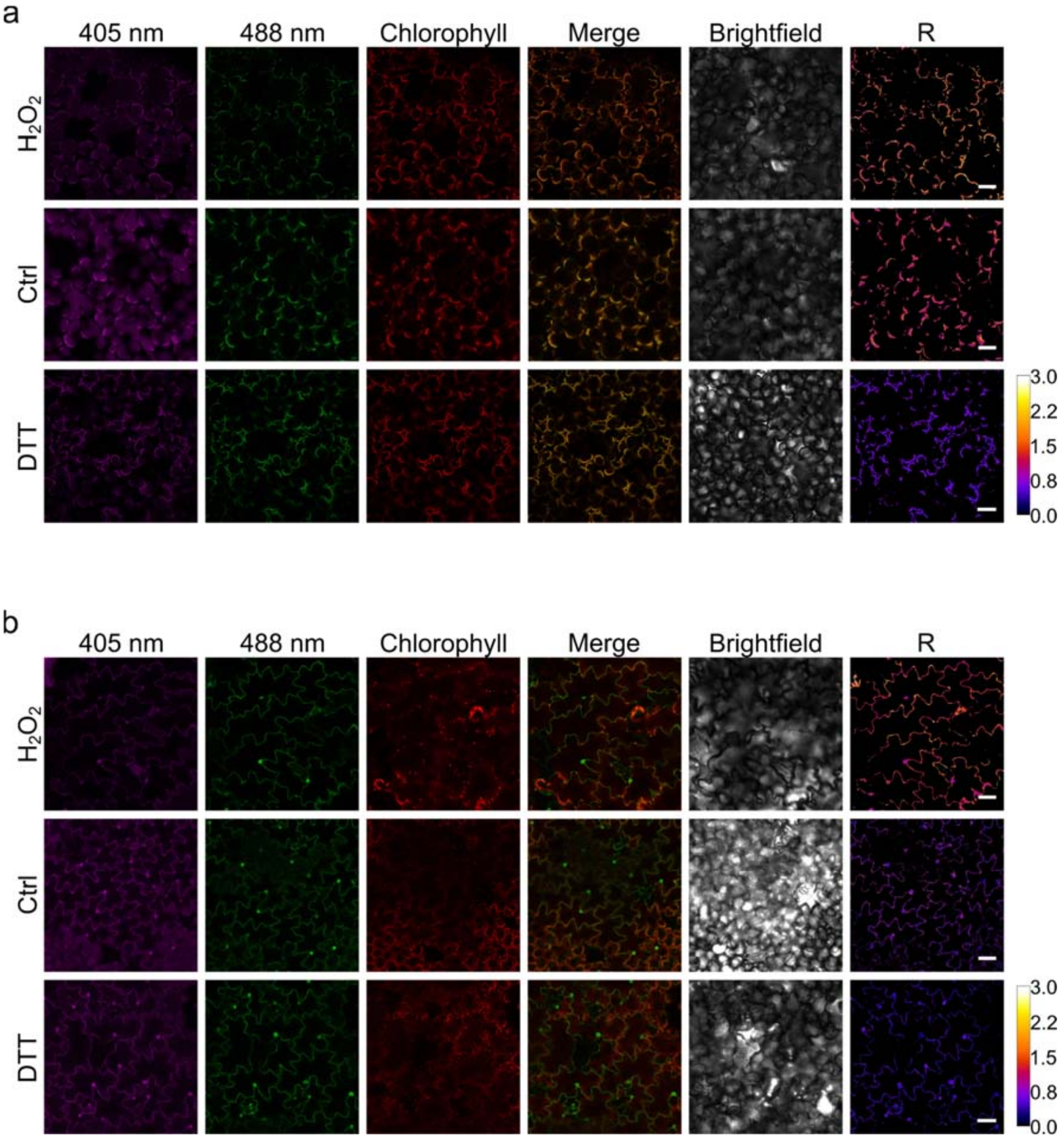
a
405 nm
488 nm
Chlorophyll
Merge
Brightfield
R
H2O2
Ctrl
DTT
3.0
2.2
1.5
0.8
0.0
b
405 nm
488 nm
Chlorophyll
Merge
Brightfield
R
H2O2
Ctrl
DTT
3.0
2.2
1.5
0.8
0.0

c

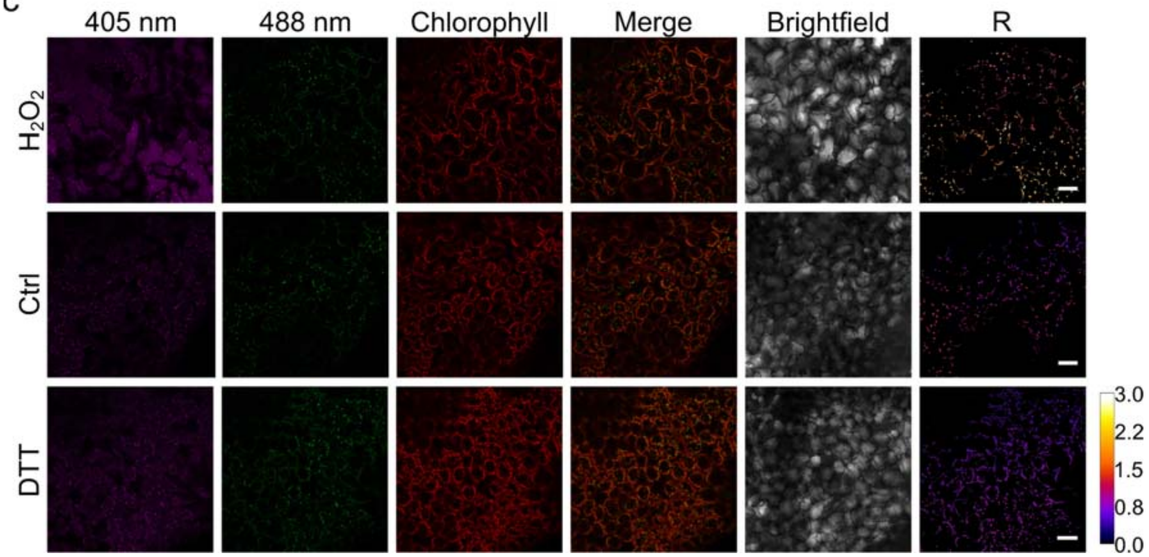
405 nm
488 nm
Chlorophyll
Merge
Brightfield
R
H_2O_2
Ctrl
DTT
3.0
2.2
1.5
0.8
0.0

d

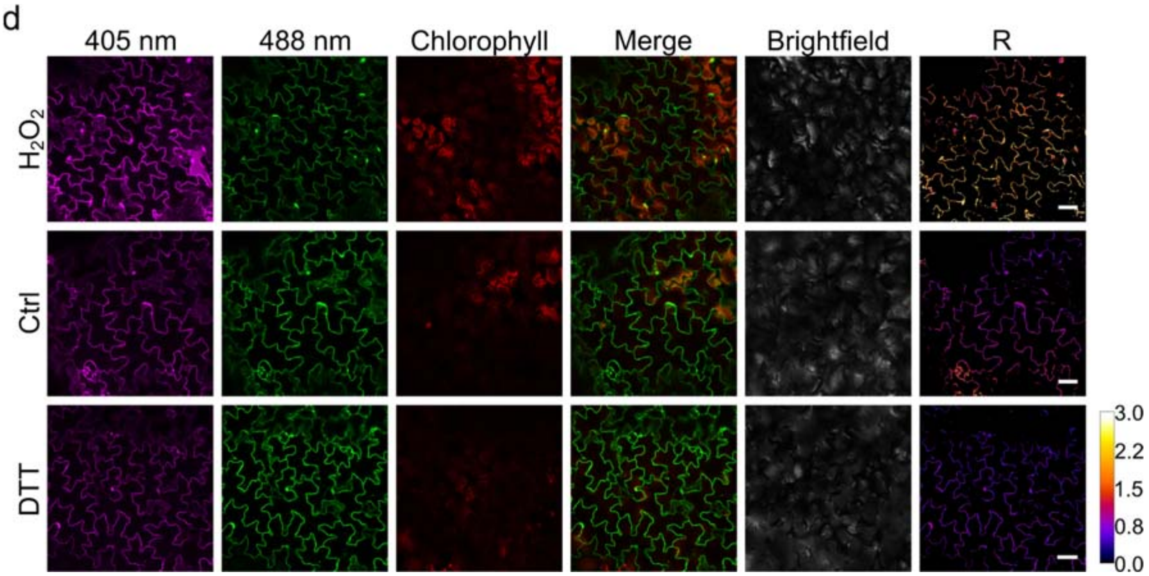
405 nm
488 nm
Chlorophyll
Merge
Brightfield
R
H_2O_2
Ctrl
DTT
3.0
2.2
1.5
0.8
0.0

e

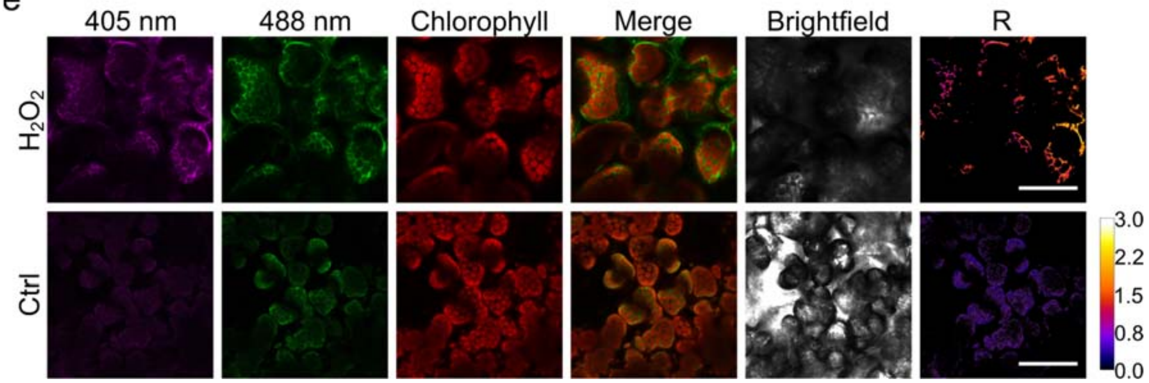
405 nm
488 nm
Chlorophyll
Merge
Brightfield
R
H_2O_2
Ctrl
3.0
2.2
1.5
0.8
0.0

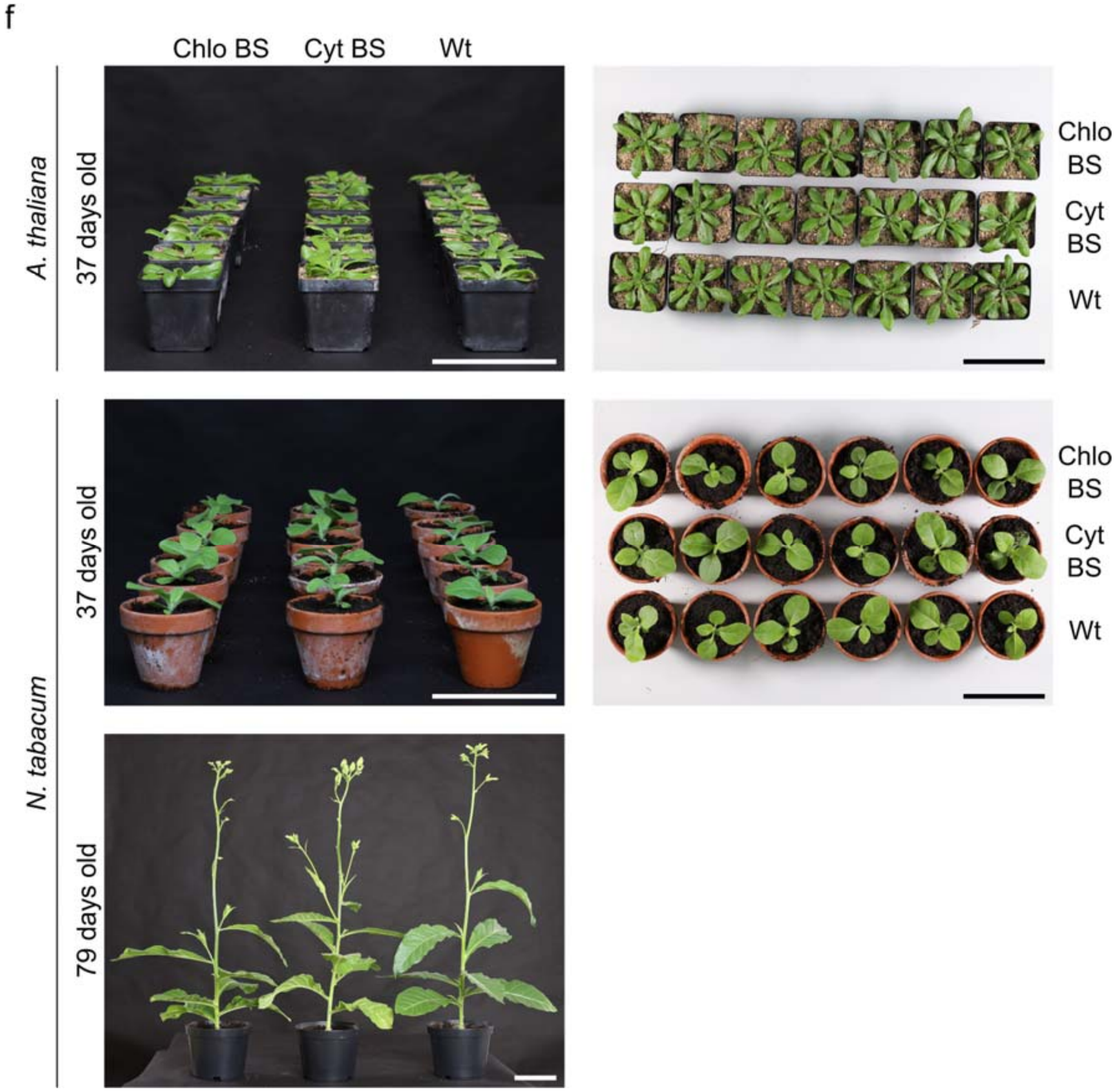
f
Chlo BS
Cyt BS
Wt
A. thaliana
37 days old
Chlo BS
Cyt BS
Wt
N. tabacum
37 days old
Chlo BS
Cyt BS
Wt
79 days old

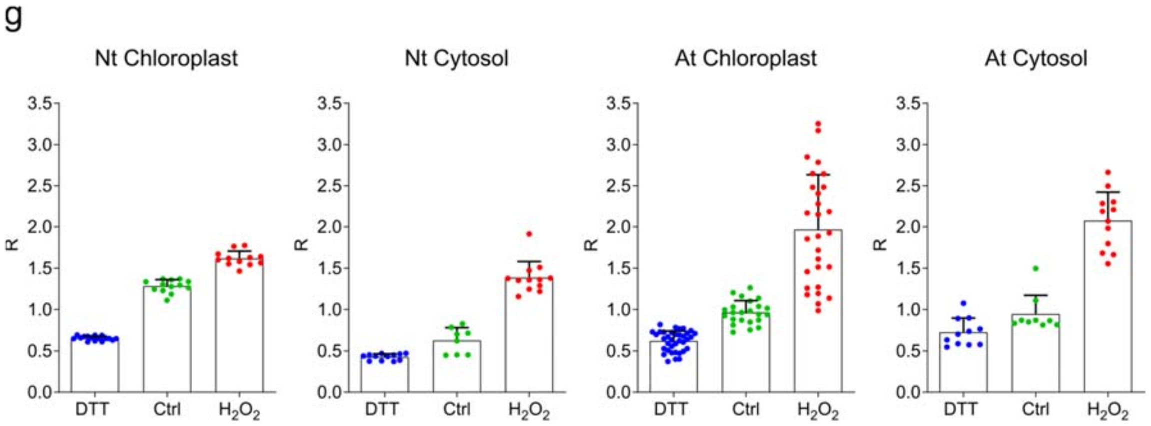
g
Nt Chloroplast
Nt Cytosol
At Chloroplast
At Cytosol
R
0.0
0.5
1.0
1.5
2.0
2.5
3.0
3.5
DTT
Ctrl
H2O2

Supplementary Figure 15. Analysis of NADP(H) dynamics in leaf cells. (a,b) Representative fluorescence images of NERNST expressed in chloroplasts **(a)** and cytosol **(b)** of tobacco mesophyll and epidermal cells, respectively, under control (Ctrl) conditions and following treatment with 10 mM H_2O_2 or 10 mM DTT. Panels show fluorescence images after excitation at 405 nm and 488 nm, chlorophyll auto-fluorescence, merge between 488-nm and chlorophyll fluorescence, brightfield and R imaging. **(c,d)** Representative fluorescence images of NERNST expressed in chloroplasts **(c)** and cytosol **(d)** of Arabidopsis mesophyll and epidermal cells, respectively, in the absence (Ctrl) and presence of 10 mM H_2O_2 or 10 mM DTT. **(e)** Representative fluorescence images of the biosensor in the cytosol of Arabidopsis and tobacco mesophyll cells. **(a-e)** Scale bars, 50 µm. Pseudocolor scales = R values. **(f)** Phenotypes of 37-days-old soil-grown Arabidopsis plants and 37-79-days-old soil-grown tobacco plants expressing NERNST biosensor in chloroplasts (Chlo BS) or the cytosol (Cyt BS). Wt, non-transformed. Scale bars, 10 cm. **(g)** Dynamic ranges and control ratios of NERNST expressed in chloroplasts or cytosol of tobacco (Nt) or Arabidopsis (At) leaf cells. Data shown as means ± SD of 8-36 cells. Source data, including all precise *n* values, are provided as a Source Data file.

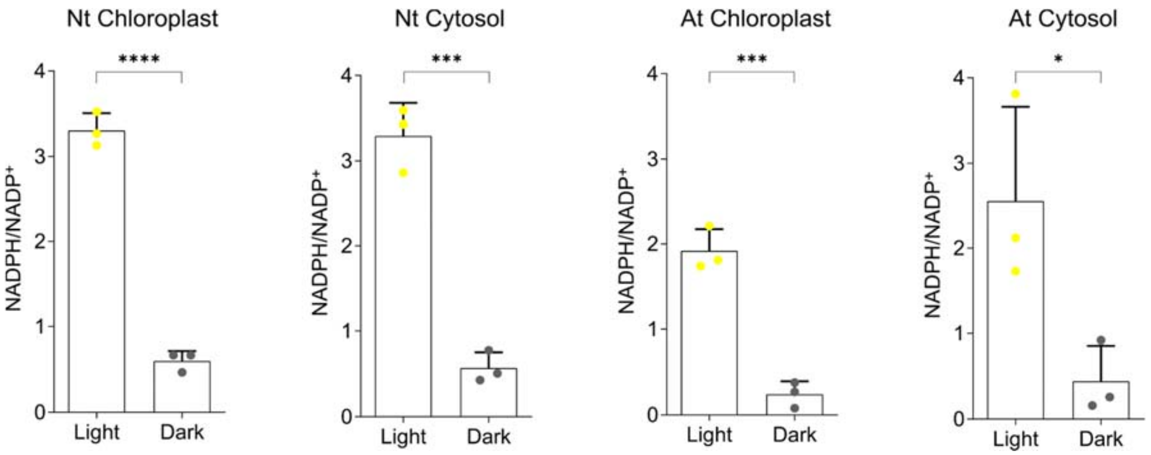

Supplementary Figure 16. Determination of $NADP^+$ and NADPH levels in whole leaf extracts of tobacco (Nt) and Arabidopsis (At). Plants were illuminated (light) or dark-adapted as described in Online Methods. The youngest fully expanded leaves of 7- (Nt) or 4-weeks-old (At) plants were used for nucleotide extraction. The terms chloroplast or cytosol only indicate the locations of NERNST in the corresponding transgenic lines, but the ratios reported reflect the NADP(H) contents of whole leaf lysates in all cases. Nucleotides were extracted using the acid/alkali method of Slater et al.[7], and their amounts determined by redox cycling. Experimental details on nucleotide extraction and dosage are also given in the Online Methods. Data shown are means ± SD of 3 independent determinations. Two-tailed Unpaired *t*-test. *$P \leq 0.05$, ***$P \leq 0.001$, ****$P \leq 0.0001$. Source data, including exact *P* values, are provided as a Source Data file.

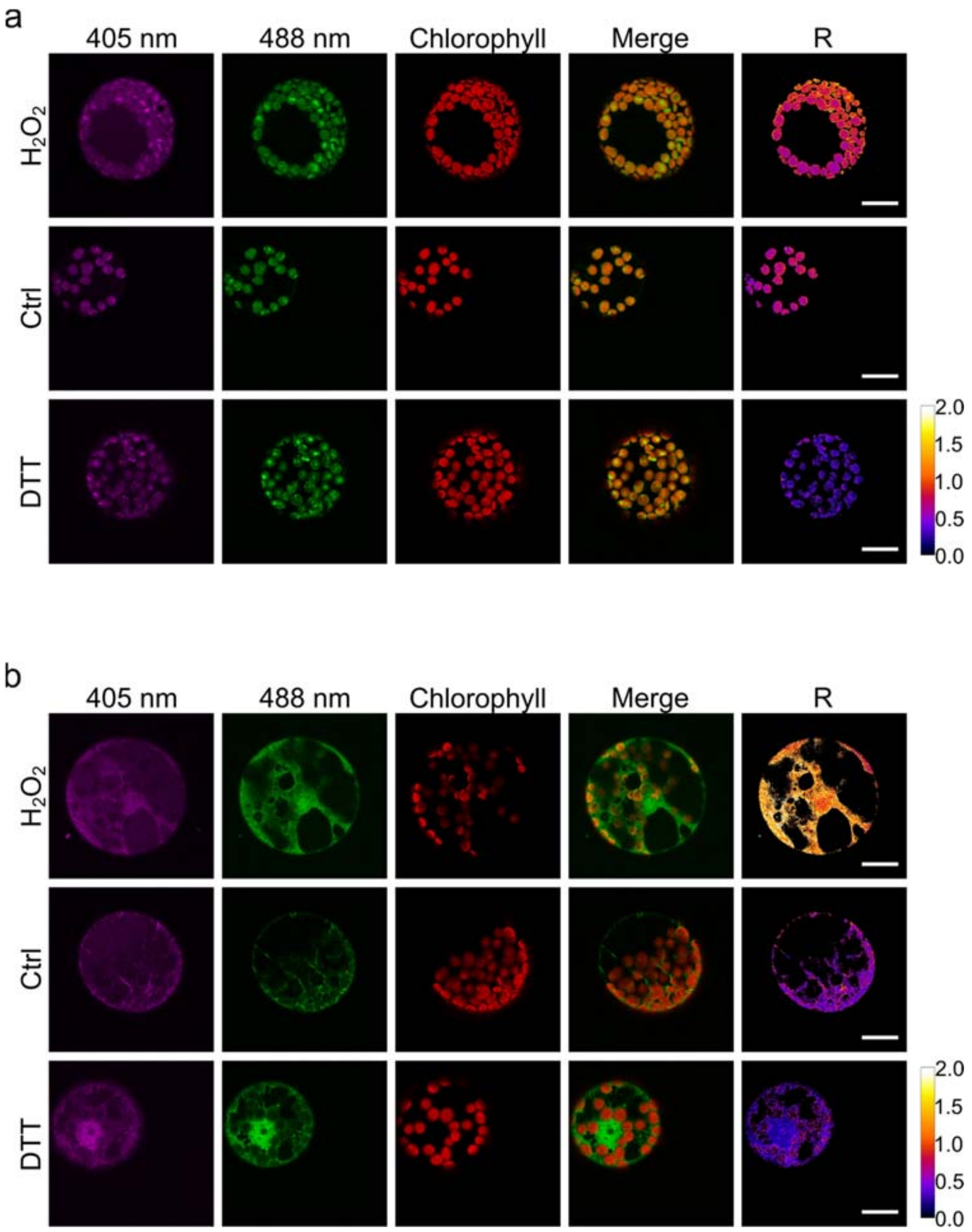
a
405 nm
488 nm
Chlorophyll
Merge
R
H_2O_2
Ctrl
DTT
2.0
1.5
1.0
0.5
0.0
b
405 nm
488 nm
Chlorophyll
Merge
R
H_2O_2
Ctrl
DTT
2.0
1.5
1.0
0.5
0.0

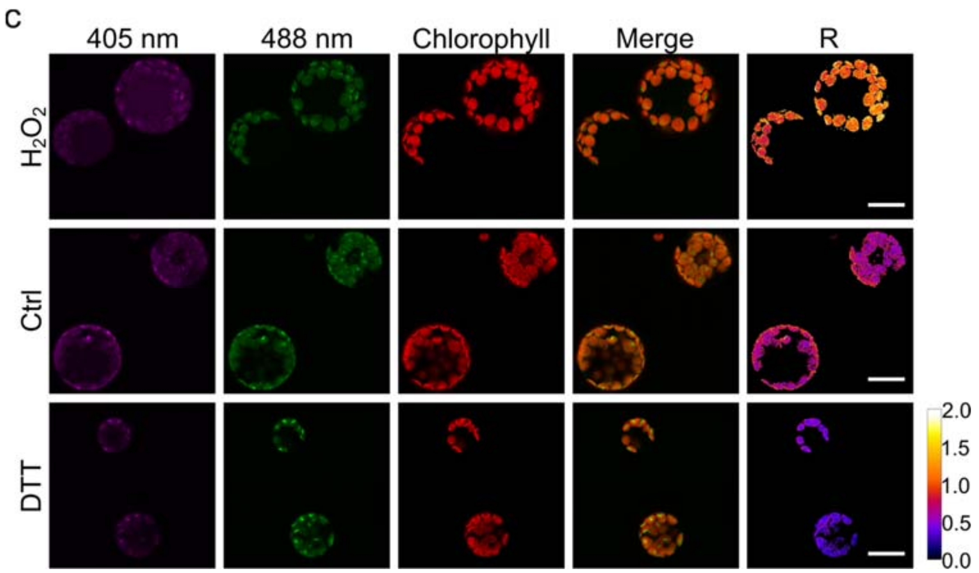
c
405 nm
488 nm
Chlorophyll
Merge
R
H2O2
Ctrl
DTT
2.0
1.5
1.0
0.5
0.0

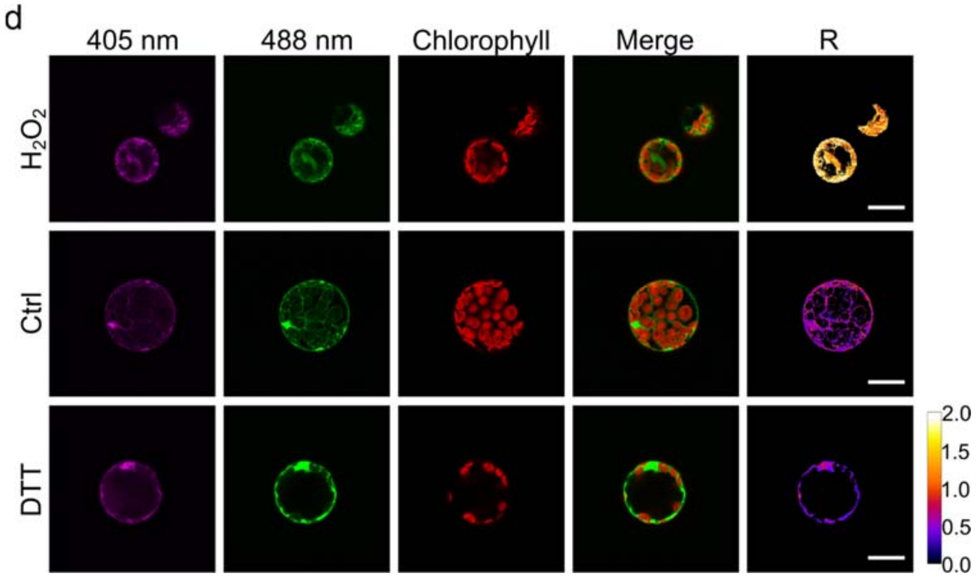
d
405 nm
488 nm
Chlorophyll
Merge
R
H2O2
Ctrl
DTT
2.0
1.5
1.0
0.5
0.0

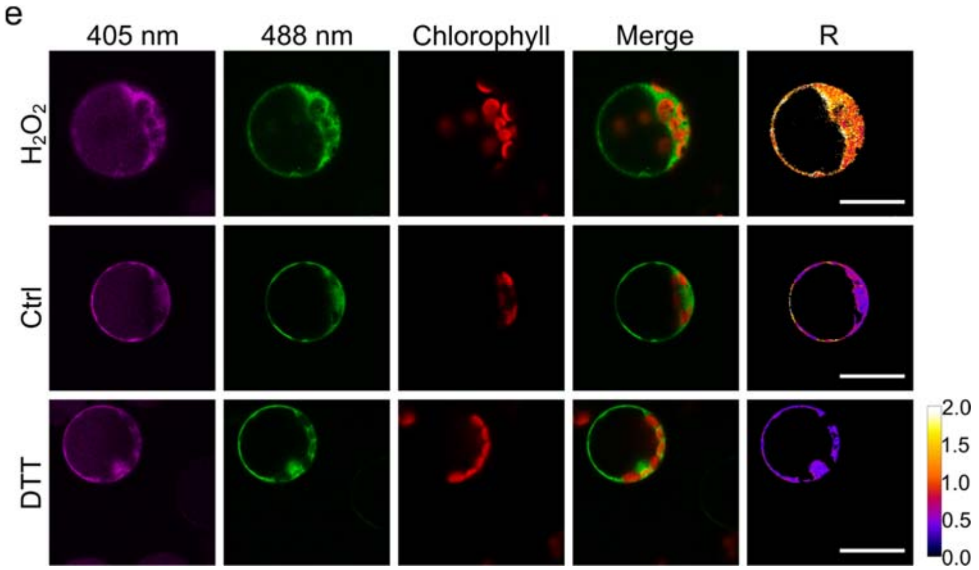

Supplementary Figure 17. NADP(H) imaging in isolated leaf protoplasts. (a-d) Representative fluorescence images of NERNST in protoplasts isolated from tobacco **(a,b)** and Arabidopsis **(c,d)** leaves expressing the biosensor in chloroplasts **(a,c)** or the cytosol **(b,d)**. Protoplasts were visualized by confocal microscopy under normal conditions (Ctrl) and after exposure to 10 mM H_2O_2 or 10 mM DTT. Panels show fluorescence signals obtained by excitation at 405 nm and 488 nm, chlorophyll auto-fluorescence, merge between the 488-nm and chlorophyll channels, and ratiometric images (R). **(e)** Representative fluorescence signals elicited by isolated Arabidopsis protoplasts transiently expressing NERNST in the cytosol and exposed or not (Ctrl) to 10 mM H_2O_2 or 10 mM DTT. Fluorescence was monitored 24 h after transformation. Determinations were carried out as described in panels **(a-d)**. Scale bars, 20 µm. Pseudocolor scales = R values. All experiments were performed at least two times.

a

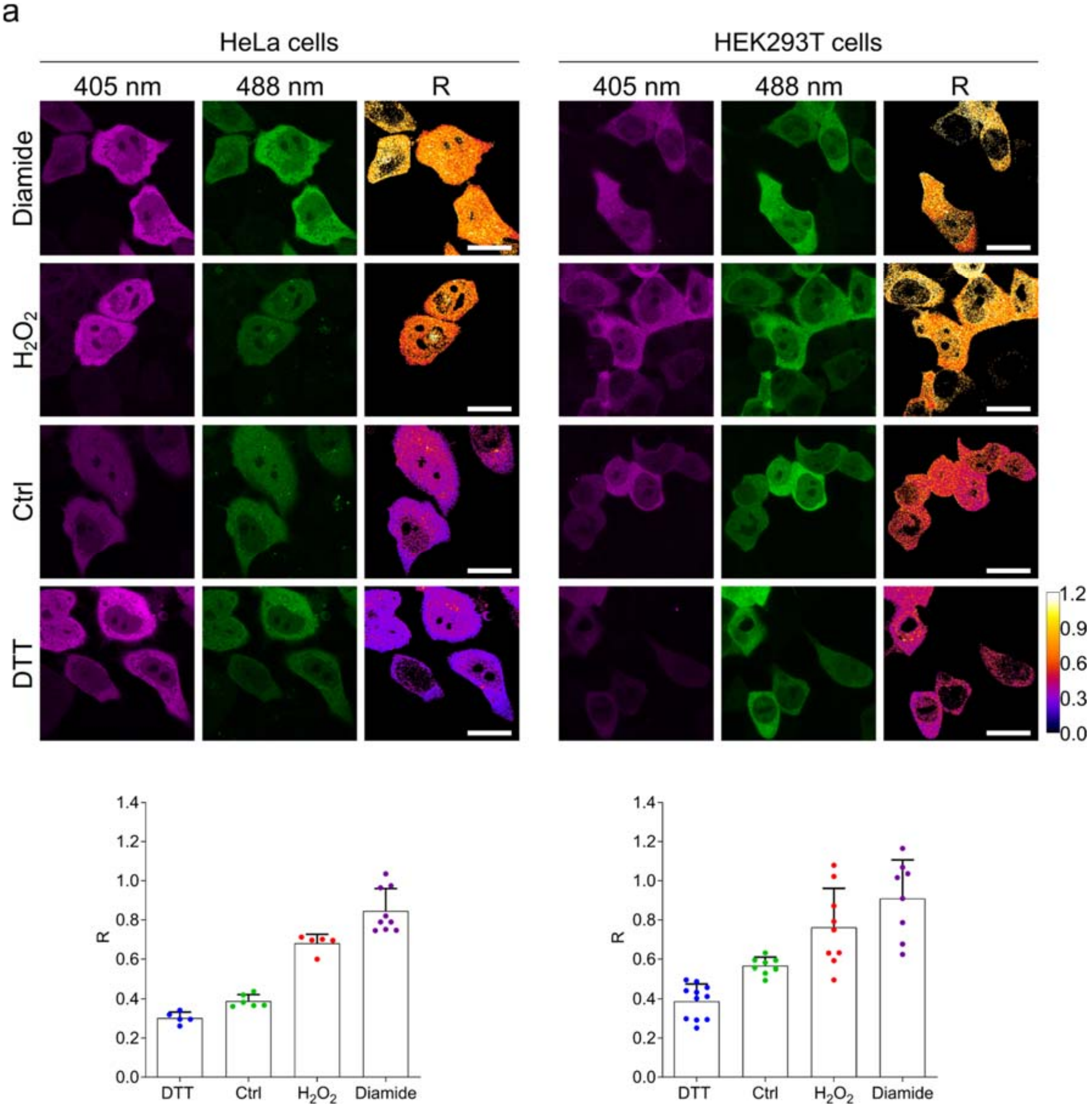

HeLa cells
HEK293T cells
405 nm
488 nm
R
Diamide
H_2O_2
Ctrl
DTT
1.2
0.9
0.6
0.3
0.0
R
DTT
Ctrl
H_2O_2
Diamide

346

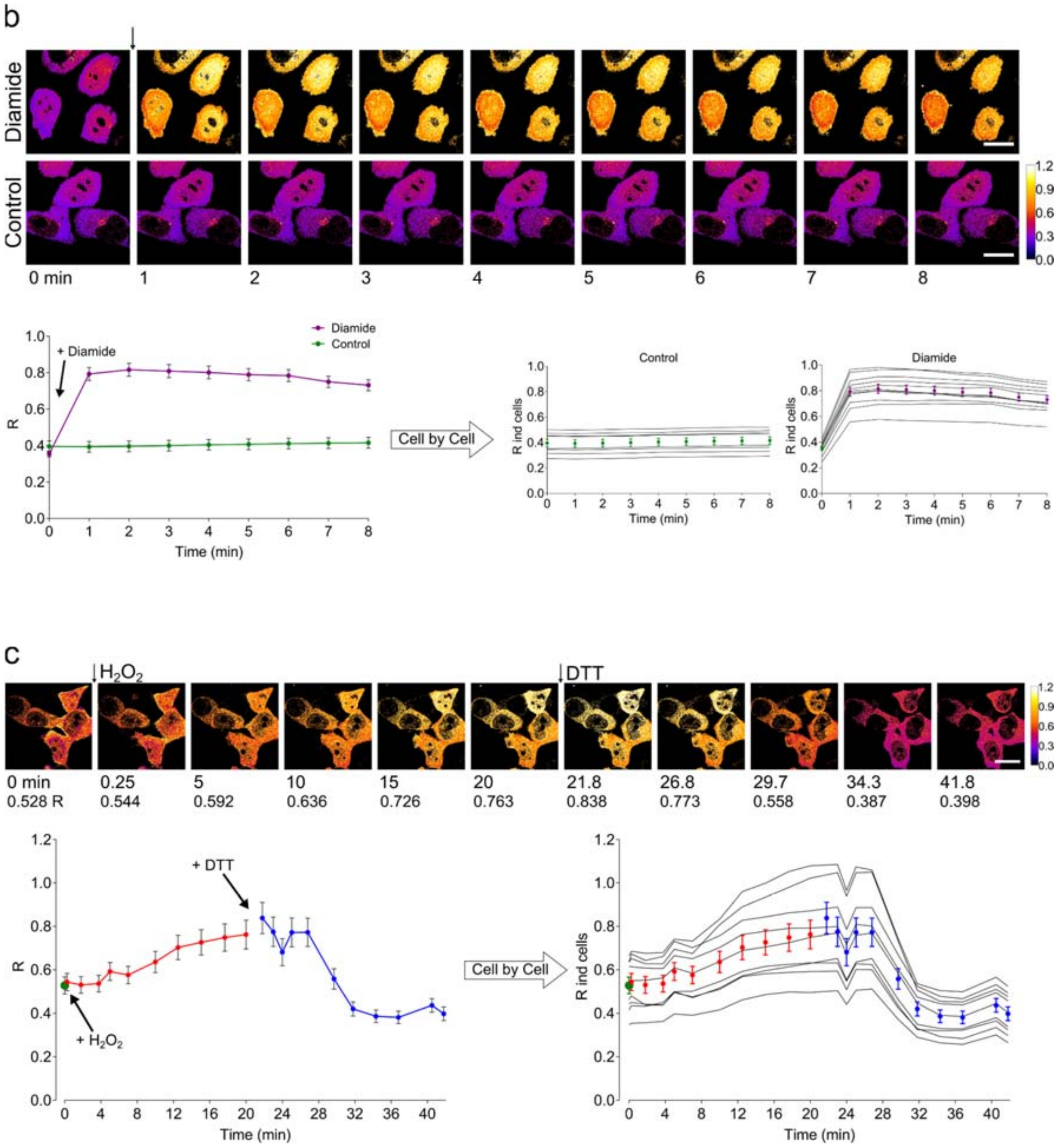

Supplementary Figure 18. Analysis of biosensor redox state in cultured mammalian cells. (a) Oxidation and reduction of NERNST expressed in HeLa and HEK-293T cells. Top, representative fluorescence images of NERNST after or not (Ctrl) cell treatments with 10 mM of either DTT, H_2O_2 or diamide. Panels show representative images obtained following excitation at 405 nm and 488 nm and the corresponding ratiometric images (R) in pseudo-color. R values for HeLa cells are shown as means ± SD of 5 (DTT and H_2O_2), 6 (Ctrl) or 9 (Diamide) cells. R values for HEK293T cells are shown as means ± SD of 11 (DTT), 8 (Ctrl and Diamide) or 9 (H_2O_2)

cells. **(b)** Oxidation of NERNST in HeLa cells exposed to 10 mM diamide showing images (top), and average and cell-by-cell time-courses (bottom) of R values. In the case of diamide, time 0 indicates cells without treatment. Data shown are means ± SEM of 11 (diamide) and 8 (control) cells. **(c)** Spatio-temporal resolution of fluorescence R responses of the NERNST biosensor expressed in HEK293T cells to successive additions of 10 mM H_2O_2 and 10 mM DTT, as shown in sequential frames (above), time-course and R values (below). Average (left) and individual cell (right) R values are shown in the lower part of the panel, and below each image. Data shown are means ± SEM of 9 cells. Time 0 (green dots) indicates cells without treatments. **(a-c)** Scale bars, 20 μm. Pseudocolor scales = R values.

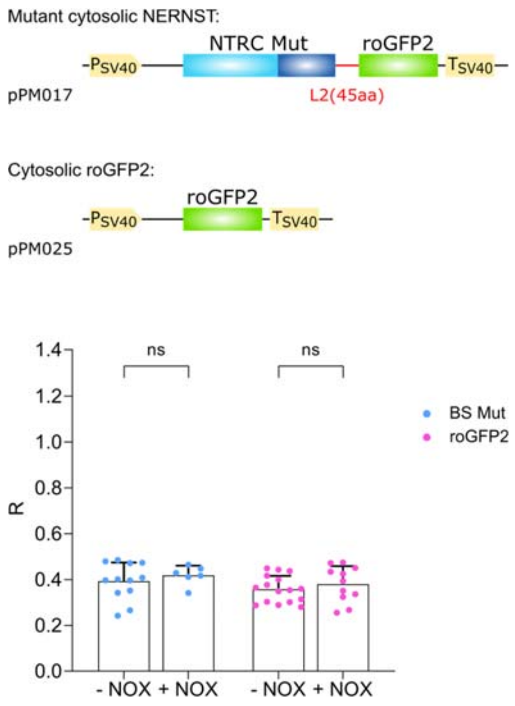

Supplementary Figure 19. Ratiometric responses of HeLa cells expressing either the Cys-to-Ser mutant of NERNST (BS Mut) or roGFP2 to the expression of NOX. R values are shown as means ± SD of 12 (-NOX) and 6 (+NOX) cells in the case of BS Mut; or 16 (-NOX) and 11 (+NOX) cells in the case of roGFP2; ns, non-significant; two-way ANOVA followed by Bonferroni's multiple comparisons test.

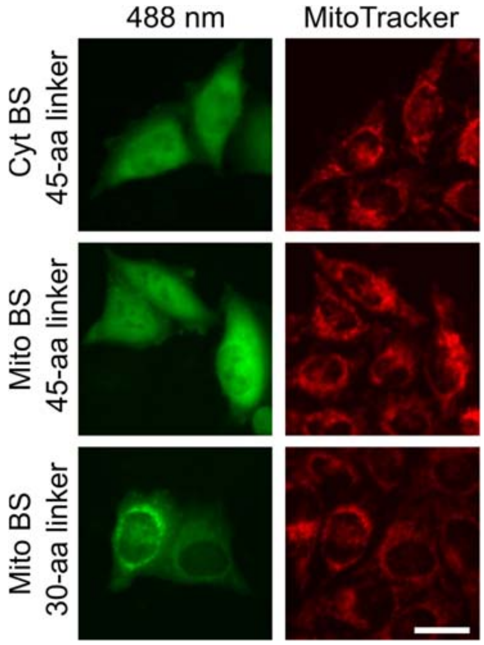

Supplementary Figure 20. The biosensor carrying the 45-aa linker could not be imported by mitochondria. Cells were incubated with a MitoTracker probe and followed by excitation at 560 nm in a PerkinElmer Operetta confocal microscope, whereas the biosensor (BS) was followed at 488 nm. Fluorescence of cytosolic- (Cyt BS) or mitochondrial-targeted (Mito BS) NERNST carrying the 45-aa linker was observed only in the cytosol, presumably representing unimported precursor protein. Fluorescence of the 30-aa Mito BS was instead recovered from mitochondria. The experiment was performed once. Representative images are shown. Scale bar, 20 μm.

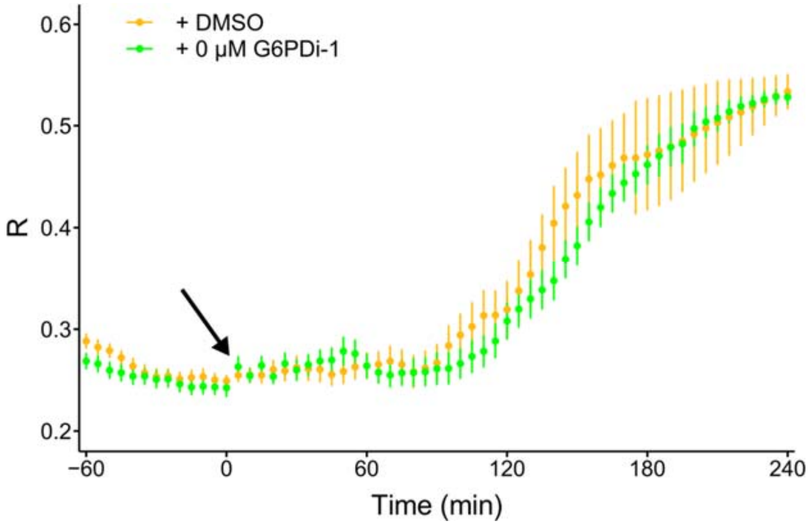

Supplementary Figure 21. Oxidation of mitochondrial NERNST under prolonged incubation of HeLa cells in LCIS solution is not caused by dimethyl sulfoxide. The time-course of R change is not affected by the addition (yellow dots) or not (green dots) of dimethyl sulfoxide (DMSO) to the imaging solution (arrow). G6PDi-1, G6PDH inhibitor. Experimental conditions are given in the Online Methods and the legend to Fig. 5e,f. Data shown are means ± SEM of 7-46 cells. Source data, including all precise *n* values, are provided as a Source Data file.

Supplementary Tables 1-4

Supplementary Table 1. Plasmids used in experiments described in this work (gray shading). Plasmids below in the hierarchy were used as intermediate vectors for cloning purposes. Arrows denote hierarchical dependence in the cloning process, e.g., pPM002 was constructed based on pHB124, pPM003 on pHB125, and so on.

Plasmid name	Description	Insert	Backbone	Cloning procedure
pPM002	P_{T7}-6xHis tag-NTRC-L1(30aa)-roGFP2-T_{T7}	NTRC-L1(30aa)-roGFP2 was excised from pHB124 with NdeI/HindIII	pET-TEV (Addgene) digested with NdeI/HindIII	Ligation with T4 DNA ligase
↳ pHB124	P_{T7}-NTRC-L1(30aa)-roGFP2-T_{T7}	The rice NTRC-coding sequence without its TP was amplified from pQE30-NTRC[8] with primers oHB272/oHB273. The roGFP2-coding region was amplified from pBinAR-roGFP2[9] using primers oHB274/oHB275. The 30-aa L1 linker was synthesized (gBlocks). Fragments were joined by Fusion PCR	pWW301[10] digested with NdeI/HindIII	Gibson[11] assembly of backbone and PCR insert
pPM003	P_{T7}-6xHis tag-NTRC-L2(45aa)-roGFP2-T_{T7}	NTRC-L2(45aa)-roGFP2 was excised from pHB125 with NdeI/HindIII	pET-TEV (Addgene) digested with NdeI/HindIII	Ligation with T4 DNA ligase
↳ pHB125	P_{T7}-NTRC-L2(45aa)-roGFP2-T_{T7}	The rice NTRC-coding sequence without its TP was amplified from pQE30-NTRC[8] with primers oHB272/oHB273. The roGFP2-coding region was amplified from pBinAR-	pWW301[10] digested with NdeI/HindIII	Gibson[11] assembly of backbone and PCR insert

			roGFP2[9] using oHB274/oHB275. The 45-aa L2 linker was synthesized (gBlocks). Fragments were joined by Fusion PCR		
pPM004		P_{T7}-NTRC(C140S,C143S,C377S,C380S)-L1(30aa)-roGFP2-6xHis tag-T_{T7}	NTRC(C140S,C143S,C377S,C380S)-L1(30aa)-roGFP2 sequence was excised from pHB129 with NdeI/HindIII	pET-TEV (Addgene) digested with NdeI/HindIII	Ligation with T4 DNA ligase
↳	pHB129	P_{T7}-NTRC(C140S,C143S,C377S,C380S)-L1(30aa)-roGFP2-T_{T7}	The C140S, C143S, C377S and C380S mutations were introduced in NTRC by PCR amplification from pHB124 using oHB181/oHB182 and oHB183/oHB184		Gibson[11] assembly of the two PCR products
pPM005		P_{T7}-NTRC(C140S,C143S,C377S,C380S)-L2(45aa)-roGFP2-6xHis tag-T_{T7}	NTRC(C140S,C143S,C377S,C380S)-L2(45aa)-roGFP2 sequence was excised from pHB130 with NdeI/HindIII	pET-TEV (Addgene) digested with NdeI/HindIII	Ligation with T4 DNA ligase
↳	pHB130	P_{T7}-NTRC(C140S,C143S,C377S,C380S)-L2(45aa)-roGFP2-T_{T7}	The C140S, C143S, C377S and C380S mutations were introduced in NTRC by PCR amplification from pHB125 using oHB181/oHB182 and oHB183/oHB184		Gibson[11] assembly of the two PCR products
pPM006		P_{35S}-TP-NTRC-L1(30aa)-roGFP2-T_{E9}	NTRC-L1(30aa)-roGFP2 sequence was PCR-amplified from pPM002 with	pCHF3-TP-FLV1[12] with FLV1 region removed by cleavage with SacI/SalI, contain the 122-bp	Ligation with T4 DNA ligase

		primers oAK001/oAK002 introducing SacI and SalI restriction sites	sequence encoding the chloroplast TP of pea FNR	
pPM008	P_{35S}-NTRC-L1(30aa)-roGFP2-T_{E9}	NTRC-L1(30aa)-roGFP2 sequence was PCR-amplified from pPM002 using oAK001/oAK002 introducing SacI and SalI restriction sites	pCHF3[13] digested with SacI/SalI	Ligation with T4 DNA ligase
pPM015	P_{SV40}-NTRC-L2(45aa)-roGFP2-T_{SV40}	NTRC-L2(45aa)-roGFP2 sequence was PCR-amplified from pHB125 using oPM001/oPM002	pMZ333[14] digested with NotI/XbaI	AQUA[15] cloning
pPM026	P_{SV40}-MTS-NTRC-L1(30aa)-roGFP2-T_{SV40}	The sequence encoding mitochondrial MTS of human COX8 was PCR-amplified from pCDNA3.1-MitoGFP[16] using oPM034/oPM035 as internal and oPM036/oPM037 as external primers in a nested PCR reaction placed in one tube	pPM014 digested with NotI	Gibson[11] assembly of backbone and PCR insert
↳ pPM014	P_{SV40}-NTRC-L1(30aa)-roGFP2-T_{SV40}	NTRC-L1(30aa)-roGFP2 sequence was PCR-amplified from pHB124 using oPM001/oPM002	pMZ333[14] digested with NotI/XbaI	AQUA[15] cloning
pPM028	P_{SV40}-MTS-NTRC-L2(45aa)-roGFP2-T_{SV40}	The sequence encoding mitochondrial MTS of human COX8 was PCR-amplified from pCDNA3.1-MitoGFP[16] using oPM034/oPM035 as internal and oPM036/oPM037 as external	pPM015 digested with NotI	Gibson[11] assembly of backbone and PCR insert

		primers in a nested PCR reaction placed in one tube		
pPM023	P_{SV40}-p40phox-mCherry-T_{SV40}	p40phox subunit sequence was synthesized (IDT) and amplified using primers oPM028/oPM029	pTB200 sequence was amplified with oTB211/oTB210	Gibson[11] assembly of the two PCR products
↳ pTB200	A P_{SV40}-driven mammalian expression vector containing mCherry[17] sequence			
pPM017	P_{SV40}-NTRC(C140S,C143S,C377S,C380S)-L2(45aa)-roGFP2-T_{SV40}	NTRC(C140S,C143S,C377S,C380S)-L2(45aa)-roGFP2 sequence was PCR-amplified from pHB130 using oPM001/oPM002	pMZ333[14] digested with NotI/XbaI	AQUA[15] cloning
pPM025	P_{SV40}-roGFP2-T_{SV40}	roGFP2 sequence was amplified from pPM014 using oPM019/oPM002	pMZ333[14] digested with NotI/XbaI	AQUA[15] cloning
pAK001	P_{SP6}-NTRC-L2(45aa)-roGFP2-T_{SV40}	NTRC-L2(45aa)-roGFP2 sequence was PCR-amplified from pPM003 using oAK003/oAK004 introducing EcoRV and XhoI restriction sites	pCS2+MT (Addgene) digested with EcoRV/XhoI	Ligation with T4 DNA ligase
pAK002	P_{SP6}-HyPerRed-T_{SV40}	HyPerRed sequence was PCR-amplified from pC1-HyPerRed[18] using oAK005/oAK006 introducing EcoRV and XhoI restriction sites	pCS2+MT (Addgene) digested with EcoRV/XhoI	Ligation with T4 DNA ligase

pTf16 (Takara Bio Inc.)	P_{araB}, *E. coli* expression vector for Trigger factor chaperone			
pGRO7 (Takara Bio Inc.)	P_{araB}, *E. coli* expression vector for GroEL-GroES chaperones			
pGTf2 (Takara Bio Inc.)	P_{zt-1}, *E. coli* expression vector for GroES-GroEL-Trigger factor chaperones			
pKJE7 (Takara Bio Inc.)	P_{araB}, *E. coli* expression vector for DnaK-DnaJ-GrpE chaperones			
pGKJE8 (Takara Bio Inc.)	P_{araB}, P_{zt-1}, *E. coli* expression vector for DnaK-DnaJ-GrpE and GroES-GroEL chaperones			

Abbreviations: COX8, subunit 8 of human cytochrome *c* oxidase; Flv1, flavo-diiron protein 1 from *Synechocystis* sp. PCC6803; FNR, pea ferredoxin-NADP$^+$ reductase; MTS, mitochondrial transit sequence; NTRC, rice NADPH-thioredoxin reductase C; P_{araB}, *Escherichia coli* araB promoter; P_{35S}, cauliflower mosaic virus 35S promoter; P_{SP6}, bacteriophage SP6 promoter; P_{SV40}, simian virus 40 early promoter; P_{T7}, bacteriophage T7 promoter; P_{zt-1}, tetracycline-inducible promoter; T_{E9}, terminator of the pea ribulose-1,5-bisphosphate carboxylase small subunit E9 gene; TP, chloroplast transit peptide; T_{SV40}, simian virus 40 early terminator; T_{T7}, bacteriophage T7 terminator.

Supplementary Table 2. Expression of NERNST variants containing 30-aa and 45-aa connecting linkers in various biological systems.

**L1: 30-aa spacer**	5’-<br>GGCAGCGGTGCAGGCAGCGGTAGCGCGGGCGCAGGTAGCGGTGCGGGCAGCGCAGGTAGCGGCGCAGGTAGCGGCAGCG<br>GTGGTGCGGGT-3’
**L2: 45-aa spacer**	5’GGTAGCAGTGGTGCAGGCAGCGCAGGTAGTGGTGCGAGCGGCGCAGCAGGTAGTGGTAGCGCGGGCAGTGCAGGTGCGG<br>GAAGCGCAGGTGGAGCAGGTAGTGGTGCAGGCAGTGGTGCAAGTGGTGCAGCCGGT-3’

		**L1: 30-aa spacer**	**L2: 45-aa spacer**
**System**		GSGAGSGSAGAGSGAGSAGSGAGSGSGGAG	GSSGAGSAGSGASGAAGSGSAGSAGAGSAGGAGS<br>AGSGASGAAG
in vitro		X	XX
Bacteria	Cytosol	X	XX
Plants	Cytosol	XX	X

(Arabidopsis and tobacco)	Chloroplast	XX	
Plants Protoplasts (Arabidopsis and tobacco)	Cytosol	XX	X
	Chloroplast	XX	
Mammalian cells (HeLa and HEK)	Cytosol	X	XX
	Mitochondria	XX	
Zebrafish	Cytosol	X	XX

One variant of the 30-aa linker and one variant of the 45-aa linker were created. Expression (X) was considered functional when proteins, cells and tissues exhibited the expected fluorescence changes. Variants reported in the article for each biological system are shown by double crosses (XX). While NERNST variants containing both the 30- and 45-aa linkers were readily expressed in the cytosol, only the 30-aa version could be imported by chloroplasts or mitochondria, and thus used in further experiments with plant cells and protoplasts, and mammalian cells in culture.

Supplementary Table 3. Oligonucleotides used in this study.

Oligonucleotide name	Sequence 5’ -> 3’
oHB272	AATTTTGTTTAACTTTAAGAAGGAGATATACATATGGATCTTGGCAAGGGAGTGGAGAAC
oHB273	TTTGTTTGACTCGATAAACTCCCTATATTCCTTC
oHB274	GTGAGCAAGGGCGAGGAGC
oHB275	GCTTCCTTTCGGGCTTTGTTAGCAGCCGGATCAAGCTTTTAGTGATGGTGATGGTGATGGCCGCTGCTGCCCTGAAAAT ACAGGTTTTCACCGCCCTTGTACAGCTCGTCCATGCCG
oHB181	GAATTTTGGAGTAGAGGTATCAGTGCAAGCGCAATAAGCGATGGAGCATCCCCACTATTTAAGGG
oHB182	CTTGCTTAGAATTGGCTTTAAGGTTCTGCTAGGACCGCTTGTTGGAGAAGTATATAGAACACAGACAAGC
oHB183	AGCGGTCCTAGCAGAACCTTAAAGCCAATTCTAAGCAAGGTTATAGATGAGTACAACGAACACGTTCATTTTG
oHB184	GCTTATTGCGCTTGCACTGATACCTCTACTCCAAAATTCATCTTCACGAGGTAATCGAAGGCG
oAK001	GAGAGCTCCAATGGATCTTGGCAAG
oAK002	GAGTCGACTTAGTGATGGTGATGG
oPM001	CTTTTTGTCTTTTATTTCAGGTCCCGGATCGAATTGCGGCCGCATGGATCTTGGCAAGGGAGTGGAG
oPM002	GTCTGGATCGAAGCTTGGGCTGCAGGTCGACTCTAGATCAGTGATGGTGATGGTGATGGCCG

pPM034	CCCGGATCGAATTGCGGCCGCATGTCCGTCCTGACGCCGCTGCTGCTGCGGGGCTTGACAGGCTCGGCCC
pPM035	CATGGTGGCGCTTCCGCTTCCGCTTCCCAACGAATGGATCTTGGCGCGCGGCACTGGGAGCCGCCGGGCC
pPM036	CTTTTTGTCTTTTATTTCAGGTCCCGGATCGAATTGCGGCCGC
pPM037	CTCCACTCCCTTGCCAAGATCCATGGTGGCGCTTCCGCTTCCG
oPM028	GTCTTTTATTTCAGGTCCCGGATCGAATTGCGAATTCCCACCATGGCTGTGGCCCAGCAGCT
oPM029	GTTATCCTCCTCGCCCTTGCTCACCATTGCTGATGGCATCGTGTTGTAGACCCTGTAGTTG
oTB211	TCAGCAATGGTGAGCAAGGG
oTB210	GCAATTCGATCCGGGACCT
oPM019	TTTTGTCTTTTATTTCAGGTCCCGGATCGAATTGCGGCCGCATGGTGAGCAAGGGCGAGGAG
oAK003	CTGATATCAATGGATCTTGGCAAG
oAK004	GTCTCGAGTTAGTGATGGTGATGG
oAK005	CTGATATCAATGGAGATGGCGAGC
oAK006	CATCTCGAGTCATTAAACCGCCTC

Underlined nucleotides indicate the restriction sites introduced in primers: oAK001, SacI; oAK002, SalI; oAK003/oAK005, EcoRV; oAK004/oAK006, XhoI.

Supplementary Table 4. Spectral characteristics of NERNST biosensor.

	NERNST oxidized	NERNST reduced
Extinction coefficient (ε) mM^{-1} cm^{-1} at 387 nm	17.7 ± 0.6	ND
Extinction coefficient (ε) mM^{-1} cm^{-1} at 488 nm	13.0 ± 0.7	21.8 ± 1.5
Fluorescence quantum yield* (Φf) $\lambda_{excitation}$ 471 nm $\lambda_{emission}$ 510-513 nm	0.66 ± 0.01	0.91 ± 0.02

Brightness ($\varepsilon_{488} \times \Phi f$)	8.58	19.84

* Relative determination of fluorescence quantum yield was carried out as described by Würth et al.[19], using fluorescein as standard dye; ND, not determined.

Supplementary note

On the estimation of redox potentials in living systems

On general grounds, the Nernst equation is derived from Gibbs' formalism and thus a part of classical (equilibrium) thermodynamics. Current advances in redox biology show, however, that individual redox pools operate under dynamic conditions in open systems, and are not in thermodynamic equilibrium with each other (reviewed in Jones, 2010[20]). For instance, the *E* values estimated for the Trx1 and GSH/GSSG couples in the mammalian cell cytosol are different, and neither is equilibrated with the NADP(H) pool[21]. Even within a given redox couple, redox potentials could vary widely among subcellular compartments[21,22]. Indeed, the advent of organelles and other cellular compartments during evolution allowed the formation of separate micro-environments that enabled specialization on particular redox chemistries[23,24]. Despite this lack of equilibrium, theoretical arguments as well as experimental observations indicate that redox potentials can be determined under many physiological conditions and used to estimate the redox drive of the corresponding couple, providing insightful information on key biological processes.

On theoretical grounds, the conceptual framework of equilibrium thermodynamics has been successfully extended to steady-state conditions and termed precisely "steady-state thermodynamics"[25]. Theoretical bases were laid down by Hatano and Sasa[26], and experimental validation came soon afterwards (see for instance[27,28]). Although living organisms are open systems, the levels of intermediate, including redox metabolites, may remain stable under many stable physiological conditions, the time sufficient to interact with specific bioreporters. Moreover, the existence of such steady-state conditions can be tested experimentally, and genetically-encoded biosensors are particularly well-suited in this sense. They can provide an estimation of the redox status of a given couple under a defined physiological or nutritional condition, and by following the time course of fluorescence readouts, determine if the pool is under steady state or changing. Differences in the redox potential of a given pair between cellular compartments can be easily monitored by targeting the probe to specific locations within the cell. Moreover, many recent observations indicate that maintenance of

the steady-state condition is quite robust for many redox couples and physiological conditions. Thiol/sulfide systems, for example, are maintained under non-equilibrium states, but if the redox active components in the cytoplasm are rapidly diffusing (as is generally the case), the cytoplasmic redox potential will be uniform throughout the compartment and will not change with time (see Fig. 7 in[20]), allowing estimation of redox potentials as a proxy of redox chemical drive. Accordingly, redox potentials determined with genetically-encoded biosensors have been reported over the years to describe the redox landscape of various biological systems, becoming state of the art[23,24,29–36]. In a fairly recent development the redox potential of chloroplast glutathione has been measured using whole-plant imaging with roGFP2[35].

While R values of genetically-encoded biosensors can provide valuable comparative information on the redox state of a determined redox active couple, we favor the use of redox potentials when this is experimentally feasible. They capture critical features of the biological systems that are not easily accounted for R values or reduced/oxidized ratios, such as the influence of pH. Redox potentials change 60 mV per each pH unit departing from pH 7, which represents a significant difference[9,37,38]. As an example, early studies have shown only small differences between mitochondrial and cytoplasmic GSH/GSSG estimates, but they did not include the consideration of a pH difference between the compartments, which makes the redox potential more reducing in the mitochondria even with the same GSH/GSSG ratios[20]. In the case of the NADP(H) pool, the progressive scarcity of protons as the pH is raised above 7 will increase the redox drive of the couple in the reducing direction for any given $NADPH/NADP^+$ ratio.

In the accompanying article, we therefore informed redox potentials in all cases in which the fluorescent emissions of the fully oxidized and reduced forms of the NERNST biosensor could be determined (Figs. 2-4). This was particularly important when comparing the redox status of the NADP(H) pair in cellular compartments with different pH (i.e., chloroplasts vs cytosol in plant cells and protoplasts, Figs. 3a and 4b), or the behavior of the pool during physiological processes involving a pH shift (i.e., the chloroplast stroma upon dark-light transitions, Fig. 3b). Incidentally, NERNST fluorescent intensities did not change

significantly with time in any of the systems assayed as proof-of-concept, until they were subjected to nutritional, environmental or pharmacological perturbations, lending support to the notion that steady-state redox states are fairly robust[20].

Redox potentials describe the drive of the redox couple under each defined condition in a direct manner that cannot be reflected by R values and NADPH/NADP$^+$ ratios, making the information more readily accessible to a broader audience of non-specialists who can profit from the use of genetically-encoded biosensors for a plethora of scientific endeavors. For the specialist, not only the R values but also the raw fluorescent readouts of each individual experiment are provided in the accompanying metadata.

Supplementary references

1. Li, Z., Nimtz, M. & Rinas, U. The metabolic potential of Escherichia coli BL21 in defined and rich medium. *Microb. Cell Fact.* **13**, 45 (2014).
2. Oh, M.-K., Rohlin, L., Kao, K. C. & Liao, J. C. Global Expression Profiling of Acetate-grown Escherichia coli. *J. Biol. Chem.* **277**, 13175–13183 (2002).
3. El-Mansi, M., Cozzone, A. J., Shiloach, J. & Eikmanns, B. J. Control of carbon flux through enzymes of central and intermediary metabolism during growth of Escherichia coli on acetate. *Curr. Opin. Microbiol.* **9**, 173–179 (2006).
4. Martínez-Gómez, K. *et al.* New insights into Escherichia coli metabolism: carbon scavenging, acetate metabolism and carbon recycling responses during growth on glycerol. *Microb. Cell Fact.* **11**, 46 (2012).
5. Chiang, C. J., Ho, Y. J., Hu, M. C. & Chao, Y. P. Rewiring of glycerol metabolism in Escherichia coli for effective production of recombinant proteins. *Biotechnol. Biofuels* **13**, 1–9 (2020).
6. Spaans, S. K., Weusthuis, R. A., van der Oost, J. & Kengen, S. W. M. NADPH-generating systems in bacteria and archaea. *Front. Microbiol.* **6**, 1–27 (2015).
7. Slater, T. F., Sawyer, B. & Straüli, U. An assay procedure for nicotinamide-adenine dinucleotides in rat liver and other tissues. *Arch. Int. Physiol. Biochim.* **72**, 427–447 (1964).
8. Serrato, A. J., Pérez-Ruiz, J. M., Spínola, M. C. & Cejudo, F. J. A Novel NADPH Thioredoxin Reductase, Localized in the Chloroplast, Which Deficiency Causes Hypersensitivity to Abiotic Stress in Arabidopsis thaliana. *J. Biol. Chem.* **279**, 43821–43827 (2004).
9. Schwarzländer, M. *et al.* Confocal imaging of glutathione redox potential in living plant cells. *J. Microsc.* **231**, 299–316 (2008).

10. Weber, C. C. *et al.* Broad-spectrum protein biosensors for class-specific detection of antibiotics. *Biotechnol. Bioeng.* **89**, 9–17 (2005).

11. Gibson, D. G. *et al.* Enzymatic assembly of DNA molecules up to several hundred kilobases. *Nat. Methods* **6**, 343–345 (2009).

12. Gómez, R. *et al.* Faster photosynthetic induction in tobacco by expressing cyanobacterial flavodiiron proteins in chloroplasts. *Photosynth. Res.* **136**, 129–138 (2018).

13. Jarvis, P. *et al.* An Arabidopsis Mutant Defective in the Plastid General Protein Import Apparatus. *Science.* **282**, 100–103 (1998).

14. Beyer, H. M. *et al.* Red Light-Regulated Reversible Nuclear Localization of Proteins in Mammalian Cells and Zebrafish. *ACS Synth. Biol.* **4**, 951–958 (2015).

15. Beyer, H. M. *et al.* AQUA Cloning: A Versatile and Simple Enzyme-Free Cloning Approach. *PLoS One* **10**, e0137652 (2015).

16. Anand, R. *et al.* MIC26 and MIC27 cooperate to regulate cardiolipin levels and the landscape of OXPHOS complexes. *Life Sci. Alliance* **3**, e202000711 (2020).

17. Shaner, N. C. *et al.* Improved monomeric red, orange and yellow fluorescent proteins derived from Discosoma sp. red fluorescent protein. *Nat. Biotechnol.* **22**, 1567–1572 (2004).

18. Ermakova, Y. G. *et al.* Red fluorescent genetically encoded indicator for intracellular hydrogen peroxide. *Nat. Commun.* **5**, 5222 (2014).

19. Würth, C., Grabolle, M., Pauli, J., Spieles, M. & Resch-Genger, U. Relative and absolute determination of fluorescence quantum yields of transparent samples. *Nat. Protoc.* **8**, 1535–1550 (2013).

20. Jones, D. P. Redox sensing: orthogonal control in cell cycle and apoptosis signalling. *J. Intern. Med.* **268**, 432–448 (2010).

21. Go, Y.-M. & Jones, D. P. Thiol/disulfide redox states in signaling and

sensing. *Crit. Rev. Biochem. Mol. Biol.* **48**, 173–181 (2013).

22. Go, Y.-M. & Jones, D. P. The Redox Proteome. *J. Biol. Chem.* **288**, 26512–26520 (2013).

23. Santolini, J., Wootton, S. A., Jackson, A. A. & Feelisch, M. The Redox architecture of physiological function. *Curr. Opin. Physiol.* **9**, 34–47 (2019).

24. Held, J. M. Redox Systems Biology: Harnessing the Sentinels of the Cysteine Redoxome. *Antioxid. Redox Signal.* **32**, 659–676 (2020).

25. Oono, Y. & Paniconi, M. Steady State Thermodynamics. *Prog. Theor. Phys. Suppl.* **130**, 29–44 (1998).

26. Hatano, T. & Sasa, S. Steady-state thermodynamics of Langevin systems. *Phys. Rev. Lett.* **86**, 3463–3466 (2001).

27. Trepagnier, E. H. *et al.* Experimental test of Hatano and Sasa's nonequilibrium steady-state equality. *Proc. Natl. Acad. Sci. U. S. A.* **101**, 15038–15041 (2004).

28. Mounier, A. & Naert, A. The Hatano-Sasa equality: Transitions between steady states in a granular gas. *EPL (Europhysics Lett.* **100**, 30002 (2012).

29. Morgan, B., Sobotta, M. C. & Dick, T. P. Measuring EGSH and H2O2 with roGFP2-based redox probes. *Free Radic. Biol. Med.* **51**, 1943–1951 (2011).

30. Aller, I., Rouhier, N. & Meyer, A. J. Development of roGFP2-derived redox probes for measurement of the glutathione redox potential in the cytosol of severely glutathione-deficient rml1 seedlings. *Front. Plant Sci.* **4**, 1–12 (2013).

31. Bilan, D. S. & Belousov, V. V. New tools for redox biology: From imaging to manipulation. *Free Radic. Biol. Med.* **109**, 167–188 (2017).

32. Wagner, S. *et al.* Multiparametric real-time sensing of cytosolic physiology

links hypoxia responses to mitochondrial electron transport. *New Phytol.* **224**, 1668–1684 (2019).

33. Nietzel, T. *et al.* Redox-mediated kick-start of mitochondrial energy metabolism drives resource-efficient seed germination. *Proc. Natl. Acad. Sci.* **117**, 741–751 (2020).

34. Haber, Z. *et al.* Resolving diurnal dynamics of the chloroplastic glutathione redox state in Arabidopsis reveals its photosynthetically derived oxidation. *Plant Cell* **33**, 1828–1844 (2021).

35. Hipsch, M. *et al.* Sensing stress responses in potato with whole-plant redox imaging. *Plant Physiol.* **187**, 618–631 (2021).

36. Ugalde, J. M. *et al.* Endoplasmic reticulum oxidoreductin provides resilience against reductive stress and hypoxic conditions by mediating luminal redox dynamics. *Plant Cell* **34**, 4007–4027 (2022).

37. Meyer, A. J. & Dick, T. P. Fluorescent Protein-Based Redox Probes. *Antioxid. Redox Signal.* **13**, 621–650 (2010).

38. Fricker, M. D. Quantitative Redox Imaging Software. *Antioxid. Redox Signal.* **24**, 752–762 (2016).

Uncropped and unprocessed scan of the Native gel of **Supplementary Fig. 1f.**

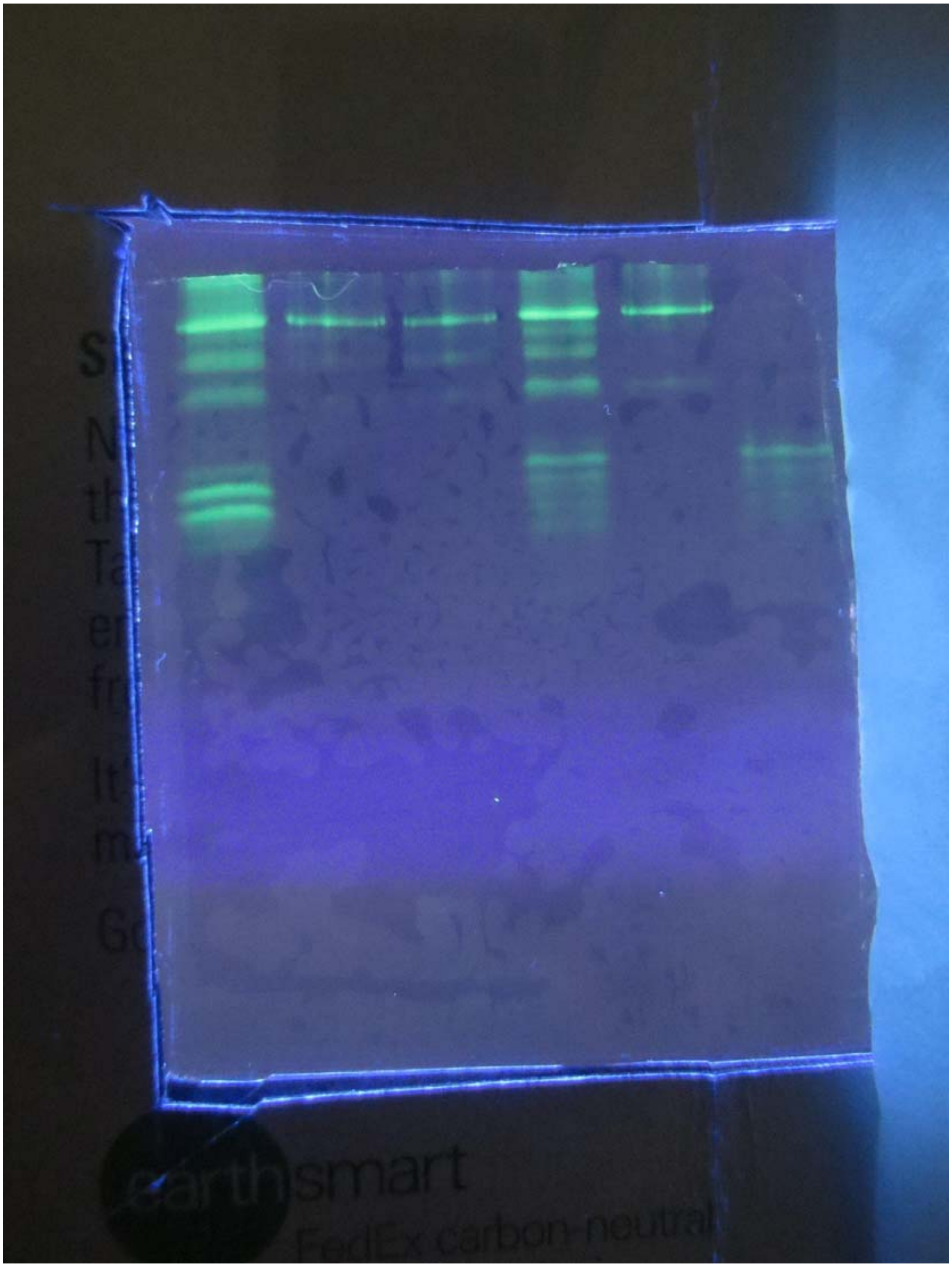

Supplement: Supplementary file 1 — Supplementary Information File [file 41467_2023_38739_MOESM1_ESM.pdf]
