# Supplementary material for: NERNST: a genetically-encoded ratiometric non-destructive sensing tool to estimate NADP(H) redox status in bacterial, plant and animal systems
Source: Nat Commun. 2023 Jun 6;14:3277. doi: 10.1038/s41467-023-38739-4 (PMC10244373; doi:10.1038/s41467-023-38739-4)
Supplement: Supplementary file 2 — Reporting Summary [file 41467_2023_38739_MOESM2_ESM.pdf]

nature portfolio

Corresponding author(s): Nestor Carrillo and Matias D. Zurbriggen

Last updated by author(s): May 5, 2023

Reporting Summary

Nature Portfolio wishes to improve the reproducibility of the work that we publish. This form provides structure for consistency and transparency in reporting. For further information on Nature Portfolio policies, see our Editorial Policies and the Editorial Policy Checklist.

Statistics

For all statistical analyses, confirm that the following items are present in the figure legend, table legend, main text, or Methods section.

n/a	Confirmed	
☐	☒	The exact sample size (n) for each experimental group/condition, given as a discrete number and unit of measurement
☐	☒	A statement on whether measurements were taken from distinct samples or whether the same sample was measured repeatedly
☐	☒	The statistical test(s) used AND whether they are one- or two-sided<br>*Only common tests should be described solely by name; describe more complex techniques in the Methods section.*
☒	☐	A description of all covariates tested
☐	☒	A description of any assumptions or corrections, such as tests of normality and adjustment for multiple comparisons
☐	☒	A full description of the statistical parameters including central tendency (e.g. means) or other basic estimates (e.g. regression coefficient) AND variation (e.g. standard deviation) or associated estimates of uncertainty (e.g. confidence intervals)
☐	☒	For null hypothesis testing, the test statistic (e.g. F, t, r) with confidence intervals, effect sizes, degrees of freedom and P value noted<br>*Give P values as exact values whenever suitable.*
☒	☐	For Bayesian analysis, information on the choice of priors and Markov chain Monte Carlo settings
☒	☐	For hierarchical and complex designs, identification of the appropriate level for tests and full reporting of outcomes
☒	☐	Estimates of effect sizes (e.g. Cohen's d, Pearson's r), indicating how they were calculated

Our web collection on statistics for biologists contains articles on many of the points above.

Software and code

Policy information about availability of computer code

Data collection

Experiments where fluorescence was measured with purified NERNST: the software used for acquisition in the plate reader is gen5 (Fig. 1b, 1c, 1d, 1e, 1f; Supplementary Fig. 4, 6c), and for acquisition in the spectrofluorometer is Cary Eclipse (Supplementary Fig. 3, 5, 6 a,b,d, 7, 8, 9).

Size-exclusion chromatography (Supplementary Fig. 1d): the software used for absorbance acquisition is UNICORN Control System 1.

For the fluorescence microscopy acquisition experiments of either NERNST or HyPerRed using a Zeiss 8000 laser scanning confocal microscope in E. coli (Fig. 2a, 2b; Supplementary Fig. 10), Arabidopsis and Nicotiana leaves (Figure 3a, 3b, 3c, 3d, 3e, 3f, 3g; Supplementary Fig. 15 a,b,c,d,e,g) and zebrafish (Fig. 6c, 6d), the microscope acquisition software is Zeiss ZEN BLACK 2.3.

For the fluorescence microscopy acquisition experiments of either NERNST or HyPerRed using an Olympus MVX10 Macro Zoom fluorescence microscope in zebrafish (Fig. 6b), the microscope acquisition software is Olympus Application Software DP2-BSW.

For the fluorescence microscopy acquisition experiments of either NERNST, roGFP2 or mCherry (the last two in the case of mammalian cells) using a Nikon Eclipse Ti C2 Plus laser scanning confocal microscope in leaf protoplasts (Fig. 4a, 4b, 4c, 4d; Supplementary Fig. 17) and mammalian cells (Fig. 5b, 5c, 5d; Supplementary Fig. 18, 19), the microscope acquisition software is NIS-Elements imaging software 5.01.

For the fluorescence microscopy acquisition experiments of NERNST using a PerkinElmer Operetta CLS confocal microscope in mammalian cells (Fig. 5e, 5f; Supplementary Fig. 20, 21), the microscope acquisition software is Harmony 4.8.

NADP/H quantification by redox cycling (Fig. 2b, Supplementary Fig. 16), or for absorption spectra of NERNST (Supplementary Fig. 2): the software used for acquisition in Shimadzu spectrophotometer is UVProbe version 3.1.

Data analysis

Data analysis, graph display and statistics were performed with GraphPad software and RStudio.
All microscopy images were analysed using the ImageJ (1.49m) software, except for Fig. 5e, 5f and Supplementary Fig. 20, 21, which were analysed with custom scripts and the CellProfiler image analysis software. In the ImageJ software, determinations of R values were corrected for background emission intensity by the "Otsu algorithm threshold ". To produce a ratio image on a pixel by pixel basis the command "Image Expression Parser" was used. Data obtained from ImageJ were imported, concatenated and prepared for analysis in RStudio (RStudio: Integrated Development for R, http://www.rstudio.com/). Data obtained from CellProfiler were further processed using the KNIME data analysis platform and RStudio.
Analysis pipelines for both software packages and Macro and R scripts used for analyzing microscopy images in ImageJ or CellProfiler are available in a GitHub repository. The DOI for this Github repository can be found in the following target URL: "NCOMMS-22-05375-T [https://zenodo.org/badge/latestdoi/538954649]".
The composition of microscopy figures was performed using ImageJ and Omero.
The analysis of DNA sequences and the construction of plasmid maps were performed with Benchling.

For manuscripts utilizing custom algorithms or software that are central to the research but not yet described in published literature, software must be made available to editors and reviewers. We strongly encourage code deposition in a community repository (e.g. GitHub). See the Nature Portfolio guidelines for submitting code & software for further information.

Data

Policy information about availability of data

All manuscripts must include a data availability statement. This statement should provide the following information, where applicable:

- Accession codes, unique identifiers, or web links for publicly available datasets
- A description of any restrictions on data availability
- For clinical datasets or third party data, please ensure that the statement adheres to our policy

All data associated with this study are available within the article and its Supplementary Information files. Source data are provided with this paper.

Research involving human participants, their data, or biological material

Policy information about studies with human participants or human data. See also policy information about sex, gender (identity/presentation), and sexual orientation and race, ethnicity and racism.

Reporting on sex and gender	The research reported in this article does not involve human participants, their data, or biological material. Therefore it does not include reporting on sex, gender, race, ethnicity, or other socially relevant grouping.
Reporting on race, ethnicity, or other socially relevant groupings	It doesn’t apply.
Population characteristics	It doesn’t apply.
Recruitment	It doesn’t apply.
Ethics oversight	It doesn’t apply.

Note that full information on the approval of the study protocol must also be provided in the manuscript.

Field-specific reporting

Please select the one below that is the best fit for your research. If you are not sure, read the appropriate sections before making your selection.

☒ Life sciences ☐ Behavioural & social sciences ☐ Ecological, evolutionary & environmental sciences

For a reference copy of the document with all sections, see nature.com/documents/nr-reporting-summary-flat.pdf

Life sciences study design

All studies must disclose on these points even when the disclosure is negative.

Sample size

Sample sizes were chosen according to the state of the art for experimental setups comprising genetically encoded biosensors in bacteria, animal and plant cells, and these numbers are according to our experience adequate for the kind of experiments and measurements performed:

Experiments in which fluorescence was measured in purified NERNST samples (Fig. 1b, 1c, 1d, 1e, 1f; Supplementary Fig. 4, 5, 6, 7, 8, 9): three independent determinations were made. In Supplementary Fig. 2, 3, representative spectra are shown.

NERNST fluorescence microscopy determinations in E. coli (Fig. 2a, 2b; and Supplementary Fig. 10): three to six independent images from different biological replicates were taken with a Zeiss 8000 confocal microscope for each condition. Representative images are shown.

NADP/H quantification by redox cycling: Fig. 2b, two independent determinations were made. In Supplementary Fig. 16, three independent determinations were made.

NERNST fluorescence microscopy determinations in Arabidopsis leaves, Nicotiana tabacum leaves or Nicotiana roots (Fig. 3; and Supplementary Fig. 15 a,b,c,d,e,g): three to six images were taken from two or three different plants with a Zeiss 8000 confocal microscope for each condition and each plant line. The resulting R of individual cells was measured with ImageJ. Representative images are shown.

NERNST fluorescence microscopy determinations in Arabidopsis and Nicotiana tabacum protoplasts (Fig. 4; and Supplementary Fig. 17): six to eleven images were taken from a protoplast suspension with a Nikon Eclipse Ti C2 Plus laser scanning confocal microscope for each condition and each plant line. The resulting R of individual protoplasts was determined with ImageJ. Representative images are shown, from to to three independent experiments.

NERNST/roGFP2/mCherry fluorescence microscopy determinations in HEK-293T or HeLa cells: five to eleven images from three different biological replicates were taken with a Nikon Eclipse Ti C2 Plus laser scanning confocal microscope for each condition in Fig. 5c, 5d; and Supplementary Fig. 18a, 19. The resulting R of individual cells was determined with ImageJ. For experiments carried out over time with Nikon Eclipse Ti C2 Plus confocal microscope (Fig. 5b; Supplementary Fig. 18 b,c), five to eleven individual cells were followed.
In experiments carried out with PerkinElmer Operetta CLS confocal microscope (Fig. 5e, 4f; Supplementary Fig. 20, 21), three images from three individual wells were taken at each time for all conditions. Representative images are shown.

NERNST/HyPerRed fluorescence microscopy determination in zebrafish (Fig. 6b, 6c, 6d): three independent images with four to six embryos each were taken. Representative images are shown.

Data exclusions

No outliers were excluded from the analysis.

Replication

All experimental data shown were produced experimentally in at least two independent experiments (different biological material, different batches of plants and/or protoplasts, different culture of bacteria and mammalian cells, and different zebrafish embryos). Data shown are representative, the total amount of experiments performed are:

Fig. 1 and Supplementary Fig. 4, 5, 6, 7, 8, 9: two to three independent experiments.
Fig. 2 and Supplementary Fig. 10: two to three independent experiments.
Fig. 3 and Supplementary Fig. 15, 16: two to three independent experiments.
Fig. 4 and Supplementary Fig. 17: two to three independent experiments.
Fig. 5 and Supplementary Fig. 18, 19, 20, 21: two to three independent experiments.
Fig. 6: three independent experiments.

Randomization

Not relevant to our study as the operator cannot influence the outcome of the measurement.

Blinding

In general, different conditions, times, transformation batches, etc. are and must be known by the operator and cannot be blinded. In any case, the operator cannot influence the outcome of the measurement.

Reporting for specific materials, systems and methods

We require information from authors about some types of materials, experimental systems and methods used in many studies. Here, indicate whether each material, system or method listed is relevant to your study. If you are not sure if a list item applies to your research, read the appropriate section before selecting a response.

Materials & experimental systems

n/a	Involved in the study
☐	☒ Antibodies
☐	☒ Eukaryotic cell lines
☒	☐ Palaeontology and archaeology
☐	☒ Animals and other organisms
☒	☐ Clinical data
☒	☐ Dual use research of concern
☐	☒ Plants

Methods

n/a	Involved in the study
☒	☐ ChIP-seq
☒	☐ Flow cytometry
☒	☐ MRI-based neuroimaging

Antibodies

Antibodies used

Anti-GFP (FL) (Santa Cruz Biotechnology, Inc., TX; https://www.scbt.com; https://www.citeab.com/antibodies/795996-sc-8334-gfp-antibody-fl), polyclonal in rabbits, unmodified, unconjugated, sc-8334, # H2014.

Validation

The GFP Antibody (FL) sc-8334 were purchased from Santa Cruz Biotechnology, Inc., as in indicated in Methods. This particular product is currently discontinued by the company and replaced by GFP (B-2) sc-9996, that was validated as follows: https://www.scbt.com/p/gfp-antibody-b-2; https://datasheets.scbt.com/sc-9996.pdf

Eukaryotic cell lines

Policy information about cell lines and Sex and Gender in Research

Cell line source(s)	HEK-293T (DMSZ no.: ACC635), HeLa (DSMZ no.: ACC57)
Authentication	HEK-293T and HELA cells fingerprint: STR analysis according to the global standard ANSI/ATCC resulted in an authentic STR profile of the reference STR database.
Mycoplasma contamination	The cells are tested for mycoplasma at the DSMZ: HEK-293T cells negative in microbiological culture, PCR assays. HELA cells negative in DAPI, microbiological culture, RNA hybridization, PCR assays.
Commonly misidentified lines (See ICLAC register)	None.

Animals and other research organisms

Policy information about studies involving animals; ARRIVE guidelines recommended for reporting animal research, and Sex and Gender in Research

Laboratory animals	The study involved a zebrafish AB line provided by the Acuario IBR-CONICET®, Rosario, Argentina.
Wild animals	The study did not involve wild animals.
Reporting on sex	To obtain embryos, male and female specimens were mated in a 3:4 ratio.
Field-collected samples	The study did not involve samples collected from the field.
Ethics oversight	Animals were handled in compliance with relevant international guidelines (Policy on Humane Care and Use of Laboratory Animals and American Veterinary Medical Association, https://zfin.org/). The CICUAL (Comité Institucional para el Cuidado y Uso de Animales de Laboratorio; Institutional Committee for the Care and Use of Laboratory Animals) from the University of Rosario, Argentina (Facultad de Ciencias Bioquímicas y Farmacéuticas – Universidad Nacional de Rosario – UNR), approved and monitored the research protocol involving zebrafish (Res. Nº 207/2018).

Note that full information on the approval of the study protocol must also be provided in the manuscript.
